# Ternary Complexes of BiI_3_/CuI and SbI_3_/CuI with Tetrahydrothiophene

**DOI:** 10.1021/acs.inorgchem.4c01147

**Published:** 2024-06-08

**Authors:** James H. Ballenger, Katherine S. Giunta, Ruby Carlson, Aaron D. Nicholas, Lucas C. Ducati, Marcos O. Oliveira de Brito, Matthias Zeller, Robert D. Pike

**Affiliations:** † Department of Chemistry, William & Mary, Williamsburg ,Virginia 23187, United States; ‡ National Security Directorate, Pacific Northwest National Laboratory, Richland ,Washington 99354,United States; § Institute of Chemistry, Universidade São Paulo, São Paulo ,SP 05508-220, Brazil; ∥ Department of Chemistry, Purdue University, West Lafayette ,Indiana 47907-2084, United States

## Abstract

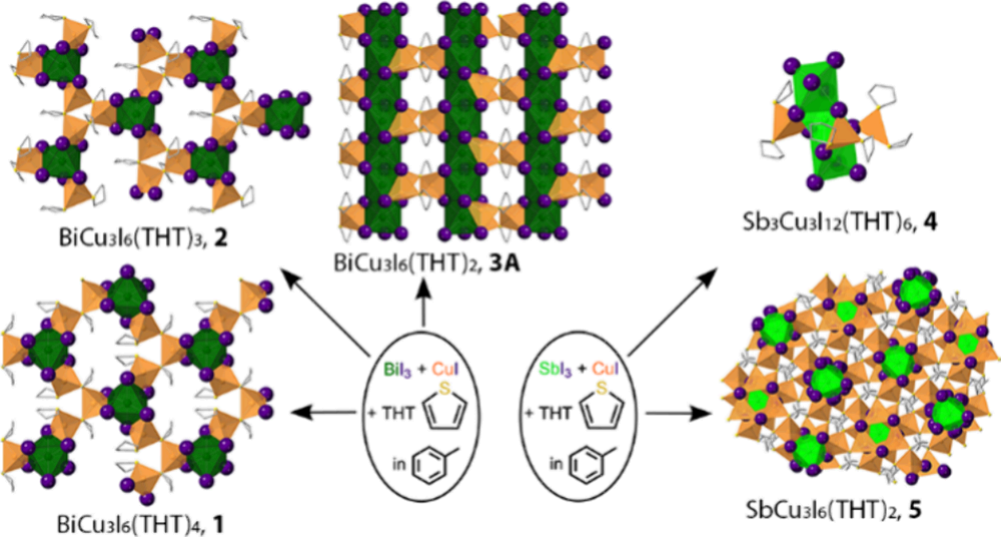

Reactions of BiI_3_/CuI mixtures with tetrahydrothiophene (THT) in toluene produce 2-D sheet networks BiCu_3_I_6_(THT)_*n*_ (*n* = 2, 3, or 4), depending on reaction conditions. All three structures are based on BiI_6_ octahedra, which share pairs of (μ_2_-I)_2_ with Cu_3_(THT)_*n*_ units. BiCu_3_I_6_(THT)_2_ features Cu_2_(μ_2_-I)_2_ rhombs with close Cu···Cu interactions and is accompanied by formation of the very complex HBi_3_Cu_12_I_22_(THT)_8_. Reactions of SbI_3_/CuI with THT in toluene produced a SbCu_3_I_6_(THT)_2_ network shows Cu_3_(μ_2_-THT)_2_ units, like its Bi congener, but Cu_6_(μ_2_-I)_6_ barrels rather than rhombs. Isolated SbI_3_ units are stacked above the Cu_6_I_6_ barrels. A molecular compound, Sb_3_Cu_3_I_12_(THT)_6_ consists of a face-sharing Sb_3_I_12_ stack, in which the Cu-THT units are bonded in asymmetric fashion about the central SbI_6_. Metal-halide bonds were investigated via QTAIM and NLMO analyses, demonstrating that these bonds are largely ionic and occur between the Bi/Sb and I p orbitals. Hirshfeld analysis shows significant H···H and H···I interactions. Diffuse reflectance spectroscopy (DRS) reveals band edges for the Bi species of 1.71–1.82 eV, while those for the neutral Sb complexes are in the range of 1.94–2.06 eV. Mapping of the electronic structure via density of state calculations indicates population of antibonding Bi/Sb–I orbitals in the excited state.

## Abstract 2

Neutral ternary networks BiCu_3_I_6_(THT)_*n*_ (THT = tetrahydrothiophene, *n* = 2, 3, 4) as well as network SbCu_3_I_6_(THT)_2_ and molecular Sb_3_Cu_3_I_12_(THT)_6_ are reported. All compounds show I-bridging between metals and THT bonded only to Cu(I). Crystal structures and Hirshfeld analysis reveals 2D sheets with exposed THT ligands. Diffuse reflectance measurements reveal bandgaps of 1.71−2.06 eV. DOS and MO calculations and frontier molecular orbitals show transitions to excited states featuring Bi/Sb−I antibonding orbitals.

## 1 Introduction

Halometallates of the p-block metals represent a structurally diverse and relatively underexplored class of compounds.^1−6^ In recent years, there has been great interest in mixed metal halide compounds. Much of the impetus behind this interest is the remarkable photovoltaic efficiencies and other optoelectronic capabilities of lead halides, such as (CH_3_NH_3_)PbX_3_ and CsPbX_3_ (X = Cl, Br, I).^7−10^ These perovskite-like semiconductor materials have demonstrated conversion efficiencies surpassing 25%; however, they suffer from significant problems, notably toxicity and poor stability. Thus, there has been increasing push toward lead-free photovoltaic materials.^11^ The group 15 metal Bi(III) and Sb(III) halides can be combined with various M(I) or alkylammonium ions to form complex perovskites, such as Cs_3_Bi_2_X_9_^12−14^ and “elpasolites,” such as Cs_2_AgBiX_6_.^15−19^ Metals such as Ag(I) and Cu(I) have been shown to reduce bandgaps.^13,15,16,20−23^ In fact, the simple double salts AgBiI_4_, AgSbI_4_, and CuBiI_4_ are themselves modest bandgap semiconductors.^24−26^

**Figure 1:**
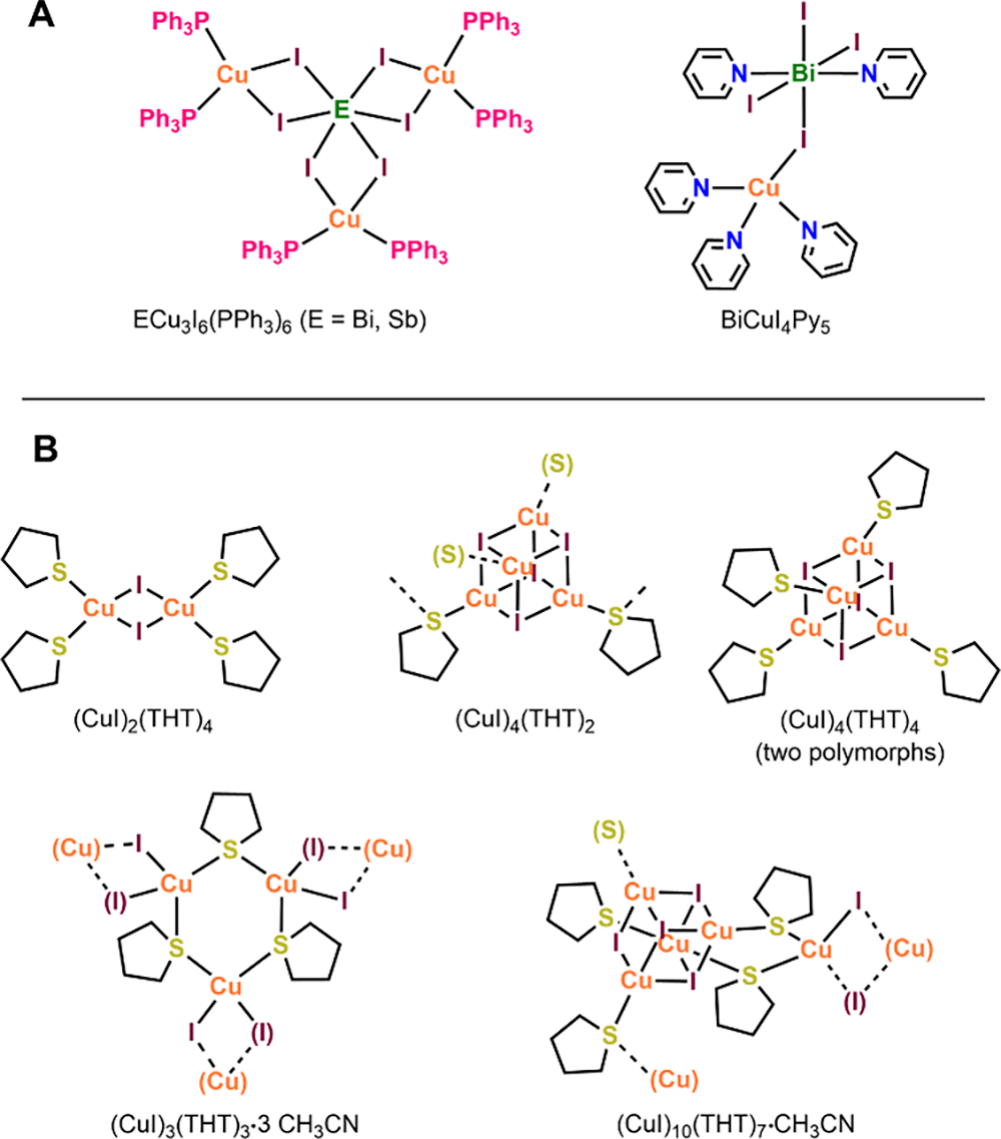
(A) Neutral ligand-supported BiI_3_/CuI and SbI_3_/CuI complexes.^47−49^ (B) CuI–THT complexes.^50^ Color key for all figures: dark green = Bi, light green = Sb, orange = Cu, purple = I, gold = S, gray = C.

With increasing recognition that cubic perovskite structures are not necessarily required for photovoltaic behavior, there is much interest in mixed halometallates of the post-transition metals. We are interested in the CuX-EX_3_ (E = Bi, Sb) as a substitute for PbX_2_ system. A good deal of literature exists for charge-separated halobismuthate(III) and haloantimonate(III) salts,^1−5,23,27−35^ much of it with alkali or organic ammonium cations. Additionally, there are multiple reports of halobismuthate and haloantimonate anions containing Cu(I) or Ag(I), such as Bi_2_Cu_2_I_10_^2–^, or paired with Cu(I) or Ag(I) cations,^23,33−46^ many of which show relatively small band gaps. However, neutral ligand-supported iodobismuthate/cuprate and iodoantimonate/cuprate compounds remain extraordinarily rare. We reported the first such compound, [BiCu_3_I_6_(PPh_3_)_6_], see Figure 1A.^47^ Thereafter, Heine and Möbs expanded the series by replacing Ag(I) for Cu(I), Sb(III) for Bi(III), and Br^–^ for I^–^.^48^ They reported a bandgap of 1.62 eV for [BiCu_3_I_6_(PPh_3_)_6_]. Heine’s group also reported the neutral Cl-bridged [Bi_2_Cu_2_Cl_8_(PPh_3_)_4_(acetone)_2_] in the same paper.^48^ We recently identified the very weakly bridged BiCuI_4_Py_5_ (Py = pyridine), Figure 1A.^49^ This simple compound which, having a long Cu–I bond, lies on the cusp of being considered an ion pair, i.e., [CuPy_3_]^+^[BiI_4_Py_2_]^−^. Diffuse reflectance spectroscopy showed a strong absorption band for BiCuI_4_Py_5_ with an optical bandgap energy of 1.94 eV. This compound is additionally interesting insofar as Py coordinates both metals. In the previous PPh_3_ complexes, the supporting ligand coordinated only the Cu(I) center with Bi/Sb coordinated strictly by halide centers.

**Figure 2:**
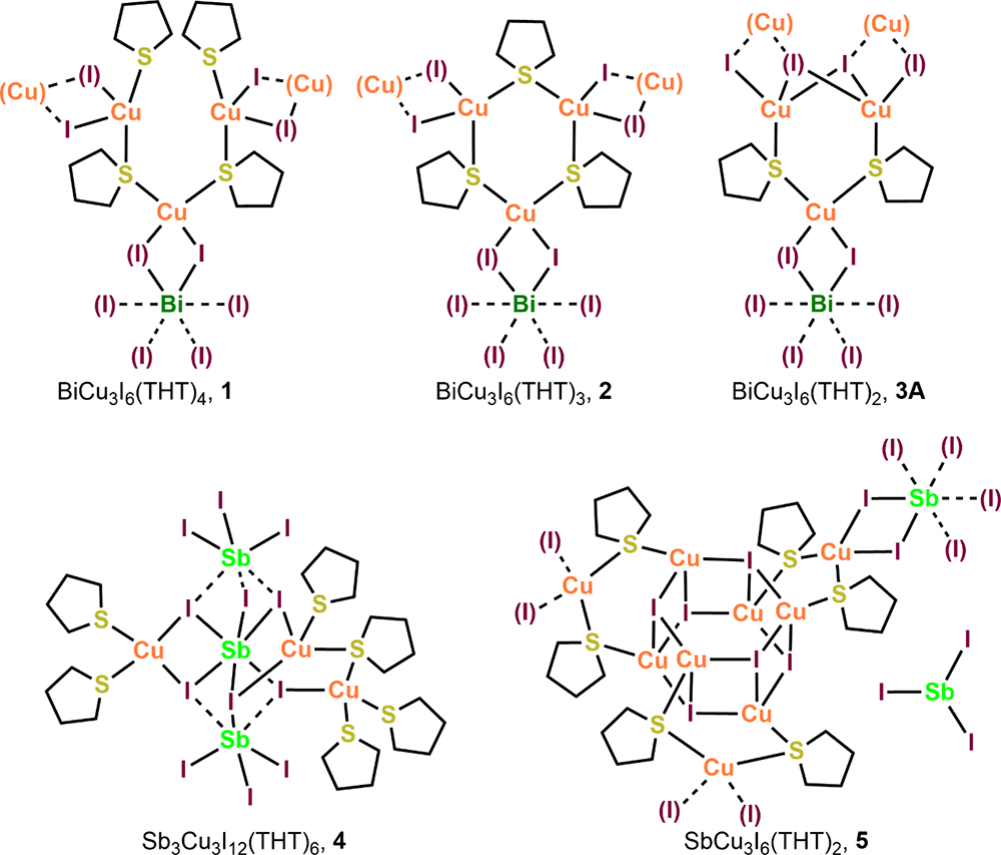
Neutral THT-supported complexes of BiI_3_/CuI and SbI_3_/CuI reported herein.

For the current study, we chose tetrahydrothiophene (THT), which is a soft ligand, like PPh_3_, and thus favors coordination to the soft Cu(I). However, unlike PPh_3_, THT tends to bridge metal centers and, along with bridging iodide, is apt to produce networks. We previously demonstrated the ability of THT to produce a diverse array of CuI products (see Figure 1B).^50^ These include a rhomboid dimer, (CuI)_2_(THT)_4_, two cubane tetramer isomers, (CuI)_4_(THT)_4_, a network based on trios of THT-bridged dimers, (CuI)_3_(THT)_3_, and a THT-bridged cubane network (CuI)_4_(THT)_2_. Herein, we report a new class of neutral iodobismuthate/cuprates and iodoantimonate/cuprates, using the THT ligand, see Figure 2.

## 2 Experimental Section

### 2.1 General

Safety precaution: THT is toxic, an irritant, and has a stench. It should be handled only with gloves and in an approved fume hood.

All reagents were purchased from Aldrich or Acros. SbI_3_ was prepared from Sb and I_2_ in toluene.^51^ CuI was purified by washing with dilute Et_3_N in ethyl ether and then vacuum drying. Toluene was dried by distilling from Na/benzophenone ketyl. Other reagents and solvents were used as received. IR spectra were collected on a Shimadzu IRTracer-100 instrument using a diamond ATR probe. Thermogravimetric analyses (TGA) were conducted using a TA Instruments Q500 in the dynamic (variable temp.) mode with a maximum heating rate of 50 °C/min to 800 °C under 40 mL/min N_2_ flow. See the SI for IR (S19–S23) and TGA (S24–S28) traces. Elemental analyses were performed by Atlantic Microlabs, Inc. of Norcross, GA.

### 2.2 Syntheses of **1**–**5**

#### BiCu_3_I_6_(THT)_4_ (**1**)

BiI_3_ (50.0 mg, 0.085 mmol), CuI (48.7 mg, 0.256 mmol), and THT (few drops, excess) were added to 5 mL of toluene in a 2-dram vial, forming a faint red solution with most reactants remaining undissolved. The vial was capped, and the mixture was heated in an oil bath, stirring at 75 °C for 1 h. The product, now a dark-red solution, was then filtered and either crystallized via layering with pentane or collected as a powder via addition to pentane with scratching, washed with pentane, and dried overnight under vacuum (104.0 mg, 0.069 mmol, 81.0%). IR: 2932, 2851, 1435, 1427, 1306, 1254, 1128, 1074, 1034, 957, 881, 812, 664 cm^–1^. Anal. calcd for C_16_H_32_BiCu_3_I_6_S_4_: C, 12.70; H, 2.13. Found: C, 12.71; H, 1.96. TGA calcd for BiCu_3_I_6_: 76.7. Found: 77.1 (60–160 °C). Calcd for 3CuI: 37.7. Found: 39.3 (265–420 °C).

#### BiCu_3_I_6_(THT)_3_ (**2**)

BiI_3_ (54.3 mg, 0.092 mmol), CuI (53.6 mg, 0.281 mmol), and THT (24.0 mg, 0.272 mmol) were added to 5 mL of toluene in a 2-dram vial, forming a faint red solution with most reactants remaining undissolved. The vial was capped, and the mixture was stirred unheated for 2 days. The product, now a bright-red powder suspended in a light-red solution, was collected via decantation, washed with pentane, and dried overnight under vacuum (90.3 mg, 0.063 mmol, 68.8%). IR: 2947, 2926, 2851, 1429, 1314, 1249, 1194, 1130, 955, 880, 806, 667 cm^–1^. Anal. calcd for C_12_H_24_BiCu_3_I_6_S_3_: C, 10.11; H, 1.70. Found: C, 10.21; H, 1.60. TGA calcd for BiCu_3_I_6_: 81.4. Found: 81.7 (80–155 °C). Calcd for 3CuI: 40.1. Found: 47.1 (255–400 °C).

#### BiCu_3_I_6_(THT)_2_ and HBi_3_Cu_12_I_22_(THT)_8_ (**3A**/**3B**)

BiI_3_ (77.3 mg, 0.131 mmol), CuI (75.0 mg, 0.394 mmol), and THT (35.4 mg, 0.397 mmol) were added to 5 mL of toluene in a 2-dram vial, forming a faint red solution with most reactants remaining undissolved. The vial was capped, and the mixture was heated in an oil bath, stirring at 75 °C for 3 days. The product, now a blood-red powder suspended in a pale-red solution, was collected via decantation, washed with pentane, and dried overnight under vacuum (152.7 mg, based on **3A**: 0.114 mmol, 86.8%). IR: 2953, 2940, 1425, 1304, 1254, 952, 881, 810, 665 cm^–1^. Anal. calcd for C_8_H_16_BiCu_3_I_6_S_2_ (**3A**) C, 7.18; H, 1.21. Found: C, 6.88; H, 1.00. TGA calcd for BiCu_3_I_6_: 86.8. Found: 87.1 (80–130 °C). Calcd for 3CuI: 42.7. Found: 48.9 (250–340 °C).

#### Sb_3_Cu_3_I_12_(THT)_6_ (**4**)

SbI_3_ (85.3 mg, 0.170 mmol), CuI (32.8 mg, 0.172 mmol), and THT (few drops, excess) were added to 5 mL of toluene in a 2-dram vial, forming a faint red solution with most reactants remaining undissolved. The vial was capped, and the mixture was heated in an oil bath, stirring at 85 °C for 2 h. The product, now a dark-orange solution, was then filtered and precipitated via addition of pentane. The orange powder product was washed with pentane, and dried overnight under vacuum (91.2 mg, 0.035 mmol, 61.8%). IR: 2928, 2849, 1429, 1252, 1196, 1069, 655, 878, 806, 667 cm^–1^. Anal. calcd for C_24_H_48_Cu_3_I_12_S_6_Sb_3_: C, 11.05; H, 1.86. Found: C, 11.08; H, 1.71. TGA calcd for Sb_3_Cu_3_I_12_: 79.7. Found: 81.5 (45–155 °C). Calcd for 3CuI: 21.9. Found: 30.0 (150–325 °C).

#### SbCu_3_I_6_(THT)_2_ (**5**)

SbI_3_ (41.5 mg, 0.083 mmol), CuI (47.0 mg, 0.247 mmol), and THT (43.5 mg, 0.493 mmol) were added to 5 mL of toluene in a 2-dram vial, forming a faint red solution with most reactants remaining undissolved. The vial was capped, and the mixture was stirred unheated for 1 day. The product, now a light-orange powder suspended in an orange solution, was collected via decantation, washed with hot pentane, and dried overnight under vacuum. (94.5 mg, 0.076 mmol, 91.5%). IR: 2949, 2930, 2849, 1427, 1304, 1250, 1206, 1157, 1128, 1070, 1032, 953, 883, 810, 665 cm^–1^. Anal. calcd for C_8_H_16_Cu_3_I_6_S_2_Sb: C, 7.69; H, 1.29. Found: C, 7.74; H, 1.15. TGA calcd for SbCu_3_I_6_: 85.9. Found: 85.8 (70–135 °C). Calcd for 3CuI: 45.7. Found: 52.1 (135–275 °C).

### 2.3 X-ray Crystallography

Crystals were grown either by carrying out the reactions described above without stirring or agitation or by layering the resulting toluene solutions with pentane. Crystals were mounted on glass fibers or on MiTeGen Micro Mounts. All measurements were made using Mo Kα radiation on a Bruker-AXS Apex three-circle diffractometer, equipped with a fine-focus sealed tube and a CCD detector (**1**, **2**, **4**), or on a Bruker-AXS D8 Venture four-circle diffractometer, equipped with a microfocus tube and a Photon 3 CPAD detector (**3A**, **3B**, **5**). Initial space group determination was based on a matrix consisting of 36 or fast scan with 180 frames. The data were reduced using SAINT+,^52^ and empirical absorption correction applied using SADABS.^53^ Structures were solved using intrinsic phasing. Least-squares refinement for all structures was carried out on *F*^*2*^. The non-hydrogen atoms were refined anisotropically. Hydrogen atoms were placed in riding positions and refined isotropically. Structure solution, refinement, and the calculation of derived results were performed using the SHELXTL package of computer programs^54^ and ShelXle.^55^ Compound **5** proved to be a pseudomerohedral twin and was solved using the twin law: [−1 0 0, 1 1 0, 0 0 –1]. Compound **3B** suffered from multiple problems: a long axis (61 Å), pseudomerohedral twinning with twin law = [−1 0 0, 0 –1 0, 0 0 1], near-whole molecule disorder (ca. 80:20), and 45 independent atoms heavier than carbon. The carbon atoms (and accompanying H atoms) were not assigned for the minor position. Four unassigned electron density peaks of 4–7 e^–^/Å^3^ are present near the half-independent Bi1 and Bi4. An H^+^ that is thought to be present to balance charge was not located. Powder diffraction (PXRD) data were collected from Parabar oil mulls of the samples on the D8 Venture instrument and were compared to calculated patterns from the single crystal results, see Figures S29–S33.

### 2.4 Photophysical Measurements

Diffuse reflectance measurements were collected at 298 K on microcrystalline powders between 350 and 950 nm using a Mikropack DH-2000-BAL deuterium and halogen light source coupled with an Ocean Optics Flame detector. Scattered light was collected with a fiberoptic cable. Spectra were referenced with BaSO_4_. Data were processed using OceanView spectroscopy software (V.2.0.8.). Raw spectra were converted to Tauc plots via Kubelka–Munk for determination of optical band gaps (see the SI).

### 2.5 Molecular Modeling and Hirschfeld Analysis

Inner sphere bonding was investigated using the QTAIM and Natural Bond Orbital (NBO)-based Natural Localized Molecular Orbital (NLMO) methods to understand and rationalize atomic orbital hybridization and involvement. Models were built from crystallographic subunits in their experimental structures and used without optimization. Rendered models can be found in the SI. Calculations were performed within the Amsterdam Density Functional (ADF) program^56^ using the scalar relativistic zeroth-order regular approximation Hamiltonian, the M06-2X DFT-KS functional, and an all-electron triple-ζ with a polarization (TZP) Slater-type basis set. The NLMO analysis was obtained with the NBO program v6.0 included in the ADF package.^57^ QTAIM utilized the corresponding module implemented in ADF. Bonds analyzed were restricted to those not adjacent to a truncated atom to reduce artifacts associated with model truncation. Three-dimensional Hirshfeld surfaces were generated with an isovalue of 0.5 au using CrystalExplorer 21.5 as implemented in the software.^58^ Surfaces were rendered as 2D fingerprint plots and delineated by atom···atom type.

## 3 Results and Discussion

### 3.1 BiI_3_/CuI/THT Synthesis

The known neutral, ligand-supported complexes of BiI_3_/CuI (Figure 1A) have been prepared by codissolving the starting salts in polar, aprotic solvents, such as tetrahydrofuran, acetone, or CH_3_CN, in which the chosen ligand is also dissolved.^47,48^ In the case of BiCuI_4_Py_5_, pyridine (Py) itself served as the solvent.^49^ Copper(I) iodide is the more difficult salt to dissolve and thus can serve as an indicator that a reaction is occurring. In the present case, the use of polar solvents led to the omission of Bi(III) from the products, with the formation of known binary products of CuI and THT.^50^ Noting that BiI_3_ shows some solubility in toluene, we switched to this solvent. Extensive testing of various BiI_3_:CuI:THT ratio and temperature combinations led to the isolation of three products, two of which were insoluble in toluene, while the other was soluble. All of these products proved to be 1:3 BiI_3_:CuI compounds, varying only in the number of supporting THT ligands: BiCu_3_I_6_(THT)_*n*_, *n* = 4 (**1** in Figure 2), *n* = 3 (**2**), and *n* = 2 (**3A**). Reaction conditions were subsequently optimized for each product. Toluene-soluble and THT-rich **1** was best prepared by heating a 1:3:excess mole ratio of BiI_3_:CuI:THT for about an hour at 75 °C and then crystallizing the product by adding alkane solvent. Toluene-insoluble, middle-THT **2** was prepared by stirring an unheated 1:3:6 mol ratio mixture of BiI_3_:CuI:THT for 2 days. Toluene-insoluble, THT-poor **3** (which proved to be a mixture of **3A** and **3B**, see below) was prepared by heating a stirred 1:3:3 mol ratio mixture of BiI_3_:CuI:THT at 75 °C for 3 days. All reactions were found to be replicable under the conditions described above:
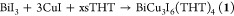

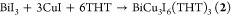

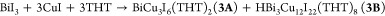


### 3.2 SbI_3_/CuI/THT Synthesis

In contrast to BiI_3_, SbI_3_ has little to no toluene solubility. Two neutral SbI_3_-CuI-THT products were identified in preliminary tests. While one of these, toluene-insoluble SbCu_3_I_6_(THT)_2_ (**5**), showed a stoichiometry analogous to Bi product **3A**, the other, toluene-soluble Sb_3_Cu_3_I_12_(THT)_6_ (**4**), proved an exception to the usual 1:3 metal ratio. Optimized reaction conditions for **4** included a 1:1:excess BiI_3_:CuI:THT ratio and heating in toluene at 85 °C for 1 day. This produced an orange solution, from which **4** was isolated upon addition of alkane solvent. Insoluble product **5** was best made by stirring a 1:3:6 SbI_3_:CuI:THT mixture in toluene at room temp. for 1 day. As with the Bi products, all Sb reactions were found to be replicable under the conditions described above:
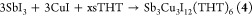

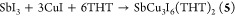


### 3.3 X-ray Crystallography

Crystals suitable for X-ray diffraction were obtained by layering toluene solutions with pentane for **1** and **4**, or, in the case of toluene-insoluble products **2**, **3A**/**3B**, and **5**, from unstirred heated reactions. Structures were solved for all compounds. The structure solution data and selected structural parameters for all compounds are presented in Tables 1 and 2, respectively. ORTEP, polyhedral, and packing diagrams are given in Figures S1–S18.

**Table 1:** Crystal and Structure Refinement Data

	**1**	**2**	**3A**	**3B**	**4**	**5**
CCDC deposit no.	2338691	2338693	2338692	2338695	2338696	2338694
color and habit	red plate	red block	red prism	red needle	orange block	orange block
size, mm	0.45 × 0.26 × 0.07	0.12 × 0.10 × 0.08	0.16 × 0.09 × 0.06	0.12 × 0.02 × 0.02	0.25 × 0.19 × 0.14	0.15 × 0.11 × 0.08
formula	C_16_H_32_BiCu_3_I_6_S_4_	C_12_H_24_BiCu_3_I_6_S_3_	C_8_H_16_BiCu_3_I_6_S_2_	C_32_H_65_Bi_3_Cu_12_I_22_S_8_	C_24_H_48_Cu_3_I_12_S_6_Sb_3_	C_8_H_16_Cu_3_I_6_S_2_Sb
formula weight	1513.65	1425.49	1337.33	4887.77	2607.65	1250.10
space group	*Pbcn*	*Cc*	*Pbcn*	*P*2	*P*2_1_/*n*	*R*3
*a*, Å	11.5774(6)	16.7943(12)	7.4460(3)	7.5659(12)	21.0600(11)	16.4016(6)
*b*, Å	17.2488(9)	16.4401(12)	18.6481(8)	10.0796(16)	11.5041(6)	16.4016(6)
*c*, Å	17.1365(9)	10.8000(8)	17.1974(7)	61.369(10)	23.8948(13)	23.0719(9)
α, deg	90	90	90	90	90	90
β, deg	90	90.3970(10)	90	90.0600(10)	101.5930(10)	90
γ, deg	90	90	90	90	90	120
volume, Å^3^	3422.1(3)	2981.8(4)	2387.92(17)	4680.1(13)	5671.0(5)	5375.1(4)
*Z*	4	4	4	2	4	9
ρ_calc_, g cm^–3^	2.938	3.175	3.720	3.395	3.054	3.476
*F*_000_	2720	2528	2336	4177	4656	4968
μ(Mo Kα), mm^–1^	12.630	14.417	17.904	15.734	9.286	11.671
temp., K	100	100	100	298	100	100
residuals: R; R_w_	0.0155, 0.0389	0.0269, 0.0616	0.0281, 0.0632	0.0851, 0.2783	0.0201, 0.0394	0.0190, 0.0400
goodness of fit	1.094	1.156	1.077	1.118	1.151	1.052
peak and hole, eÅ^–3^	0.759, –0.709	1.348, –1.137	1.375, –1.902	6.863, −5.078	0.709, –0.744	1.030, –0.717

**Table 2:** Selected Bond Lengths and Angles for **1**–**5**^a^

	**E–I**^b^	**Cu–I**	**Cu–S**	**Cu···Cu**	**I–E–I**^b^**^,^**^c^	**I–Cu–I**	**S–Cu–I**	**S–Cu–S**
**1**	3.0636(2),	2.6309(4),	2.2956(9),	–	87.134(6)–93.245(6)	106.403(15),	104.20(2)–112.46(3)	110.09(3),
	3.0803(3),	2.6394(4),	2.3071(8),			110.76(2)		112.46(3),
	3.0842(2)	2.6572(5)	2.3333(8)					115.87(4)
**2**	3.0457(13)–3.1010(11)	2.578(2)–2.648(2)	2.277(4)–2.319(4)	–	85.33(3)–95.59(4)	105.28(8),	99.03(12)–121.61(12)	101.14(17),
						106.70(9),		105.97(18),
						107.27(8)		108.63(16)
**3A**	2.572(19)–3.206(15)	2.5582(10),	2.3003(18),	2.6772(17)	84.06(3)–99.1(6)	107.63(5),	93.45(5)–124.15(5)	110.99(10)
		2.6287(8),	2.314(2)			108.65(3),		
		2.6599(9),				110.50(3),		
		2.7089(9)				120.18(3)		
**3B**	2.986(4)–3.254(4)	2.511(9)–2.822(11)	2.28(2)–2.380(19)	2.659(13)–3.035(13)	83.51(12)–101.0(3)	102.6(3)–123.(4)	94.0(5)–127.9(7)	108.1(7)–122.6(6)
**4**	2.7603(4)–3.0473(4)	2.5898(6)–2.7148(6)	2.2701(11)–2.2917(11)	–	87.903(10)–98.555(12)	100.992(18),	93.09(3)–109.54(3)	100.99(4)–124.26(4)
						111.33(2)		
**5**	2.9496(10), 3.0905(10)	2.5619(15)–2.7655(16)	2.311(3)– 2.319(2)	2.6258(16)	88.871(17), 88.99(3)	106.00(5)–122.22(5)	96.47(8)–123.50(8)	105.46(8)


a Major atom positions only in cases of disorder.

b E = Bi or Sb.

c Cis angles only.

**Figure 3:**
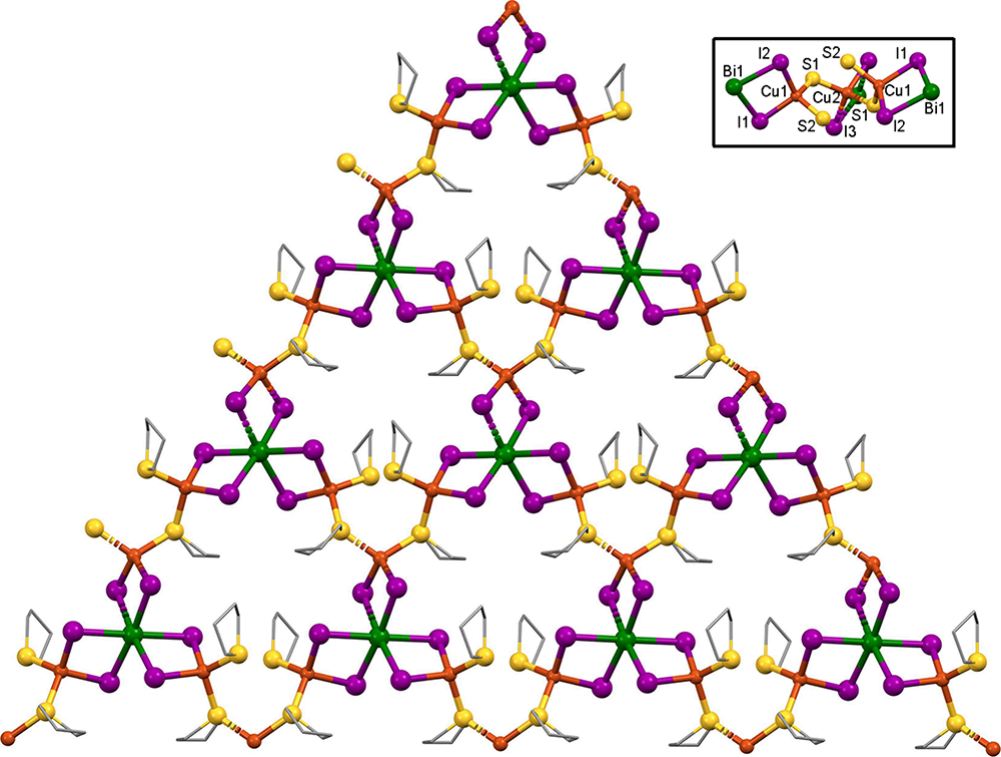
X-ray structure of compound **1** viewed along the *c*-axis. Hydrogen atoms omitted for clarity. Inset: core viewed along the *a*-axis. Carbon and hydrogen atoms omitted for clarity.

Compound **1** consisted of red plates crystallized from toluene/pentane. It solved in orthorhombic space group *Pbcn*, as half-independent. All Bi–I, Cu–I, and Cu–S bond lengths were within conventional ranges.^32−50^ Bond angles around Bi were within 3° of 90° octahedral (O_h_) and around Cu within 6.5° of 109.5° tetrahedral (*T*_d_). The structure is composed of O_h_ BiI_6_ units that are bridged to *T*_d_ CuI_2_(THT)_2_ units via pairs of μ_2_-I, forming via Cu(μ_2_-I)_2_Bi rhombs, see Figure 3. The single Bi atom and one of the two Cu atoms are half-independent, lying on a glide plane running parallel to the *ab*-plane. The Cu *T*_d_ are of two types. Cu1 is bound to one terminal THT and one bridging THT molecule, while both THT ligands attached to Cu2 are bridging. This arrangement produces Cu_3_ arcs: (THT)Cu(μ_2_-THT)Cu(μ_2_-THT)Cu-(THT) with the central Cu2 lying on the glide plane. Trios of these arcs are linked by trios of Bi centers, forming macrocycles. Compound **1** differs from **2** (see below) insofar as the Cu_3_ units are open arcs with four THT ligands, rather than closed rings with three THTs. Thus, in **1**, there are two terminal THT ligands (S2) that lie in close proximity where the arc is nearly closed. The resulting steric effect produces a twist in the Cu_3_S_4_ arc (see Figure 3 inset). The overall structure forms a 2-D planar network running parallel to the *ab*-plane. The sheet core comprises the BiCu_3_I_6_ moiety, with Bi1 and Cu2 being coplanar along the central glide plane, making this Bi(μ_2_-I)_2_Cu rhomb fully planar. Cu1 is displaced from the glide plane by 0.363 Å and from the three independent I atoms by 1.483–1.952 Å. Bi1 is nearly coplanar with the CuI_2_ in the rhomb, being displaced by 0.139 Å. Because the THT rings fold away from the Cu atoms, the hydrocarbon portion of these ligands lies on the exterior of the sheets, participating in the intersheet contacts (see Hirshfeld analysis results below). The farthest carbon atom from the central plane (C7) lies 3.827 Å away from it.

**Figure 4:**
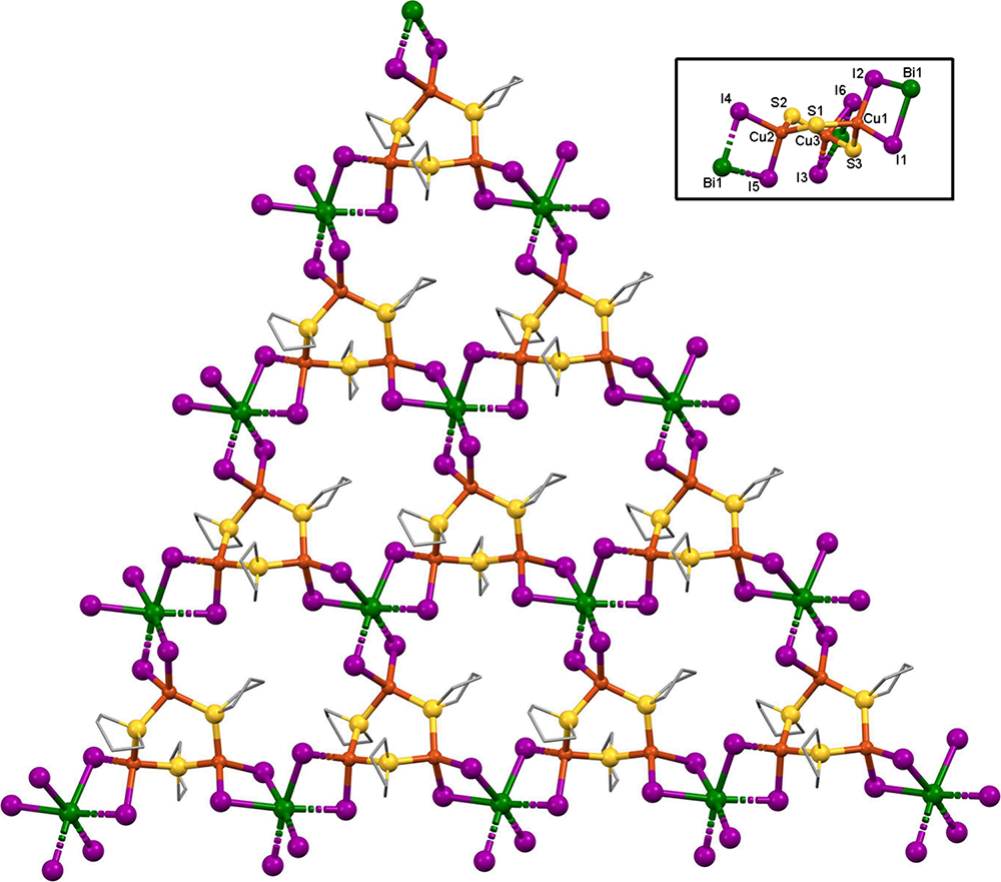
X-ray structure of compound **2** viewed along the *c*-axis. Hydrogen atoms omitted for clarity. Inset: core viewed along the *a*-axis. Carbon and hydrogen atoms omitted for clarity.

Compound **2** formed red blocks from hot toluene that solved in monoclinic space group *Cc*. All atoms are crystallographically independent. All heavy-atom bonds are of typical lengths. Bond angles around Bi were within 6° of O_h_ and around Cu within 12° of *T*_d_. The structure is nearly identical to **1**, but has one less THT ligand per formula unit, see Figure 4. This results in closure of the Cu_3_(THT)_4_ arcs seen in **1** to Cu_3_(THT)_3_ rings in **2**. These 6-membered rings adopt a twist-boat conformation and are structurally very similar to those previously found in (CuI)_3_(THT)_3_·3CH_3_CN (Figure 1B).^50^ The Bi(μ_2_-I)_2_Cu rhombs in **2** are slightly nonplanar, with Bi1 lying at distances of 0.517, 0.817, and 0.945 Å from the planes defined by CuI_2_. As is the case with **1**, compound **2** forms 2-D sheets, in this case running parallel to the *ac*-plane. Also as with **1**, the outermost atoms in the sheets are hydrogens in the THT ligands. This arrangement results in C–H···H–C and C–H···I contacts between sheets (see Hirshfeld analysis below).

**Figure 5:**
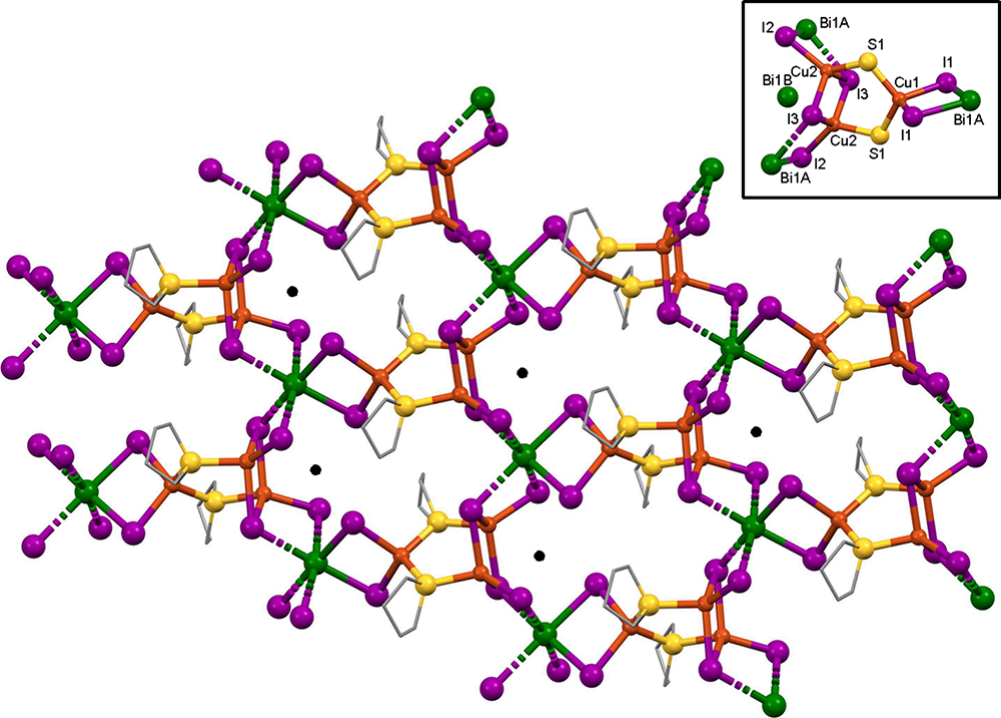
X-ray structure of compound **3A** viewed along the *c*-axis. Hydrogen atoms omitted for clarity. Location of the minor Bi1 position indicated by black dots. Inset: core viewed along the *a*-axis. Carbon and hydrogen atoms omitted for clarity.

When prepared in unstirred toluene, compound **3** yielded a combination of red blocks and needles. These habits corresponded to two closely related compounds. The blocks solved as BiCu_3_I_6_(THT)_2_, **3A**, in orthorhombic space group *Pbcn*. The half-independent structure is centered on a 2-fold rotation axis. The connectivity again shares much in common with **1** and **2**. The overall structure is a 2-D sheet. The Cu_3_S_3_ rings seen in **2** have collapsed further in **3**, replacing the lost THT bridge with a pair of iodides that are shared between Bi1 and a pair of Cu2 atoms as a Cu_2_(μ_3_-I)_2_ rhomb, see Figure 5. There is a short (2.6772 Å) bond distance imposed between these Cu2 atoms, which represents a cuprophilic interaction, being shorter than the 2.8 Å sum of the van der Waal radii for Cu.^59^ The metal bond lengths to the μ_3_-I3 atom (Bi1–I3 = 3.1885(5), Cu2–I3 = 2.6599(9), and 2.7089(9) Å) are somewhat longer than those for μ_2_-I1 and I2 atoms, but by no more than 0.15 Å. The resulting Cu_3_ unit, including the rhomb, has the formula Cu_3_S_2_I_6_. A similar secondary building unit (SBU) was observed for (CuI)_10_(THT)_7_·CH_3_CN, where it linked Cu–I cubanes to dimers.^50^ The bond angles about Bi are within 3.5° of O_h_, and those about the nondimer Cu1 atom are within 2° of *T*_d_. However, the constraints of the Cu_2_I_2_ rhombic dimer and Cu_3_S_2_ ring produce angles around Cu2 of 93.46–124.14°. The angles within the 5-membered Cu_3_S_2_ ring are within 5° of the 108° for a regular pentagon. The ring is nearly planar with atom deviations from the least-squares plane of no more than 0.074 Å.

The bridging of BiI_6_ O_h_ by the Cu_2_I_2_ rhombs within the sheets in **3A** creates O_h_ I_6_-coordinated vacancies between them (indicated by dots in Figure 5). This alternate O_h_ is an equally felicitous environment for Bi, and as a result, the Bi1 atom is disordered into this position. The main position (Bi1A) represents 98.4% of the occupancy, while the minor (Bi1B) represents only 1.6%, albeit still a meaningful amount of electron density due to the heaviness of Bi.

**Figure 6:**
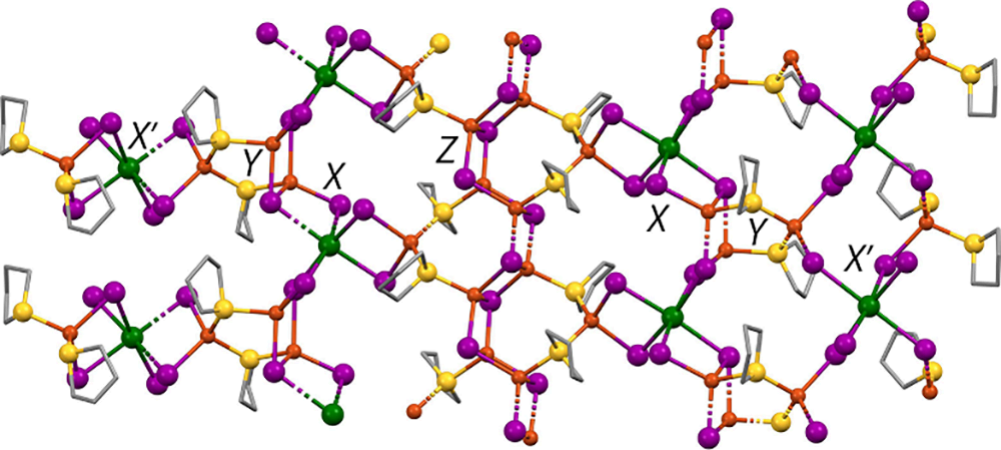
X-ray structure of compound **3B** viewed along the *b*-axis. Hydrogen atoms and near-whole-molecule disorder omitted for clarity. *X* and *X′* = BiI_3_ stacks, *Y* = Cu_3_S_2_ rings, and *Z* = (Cu_2_I_2_)_∞_ ladder.

The needles in the compound **3** mixture (**3B**) represented a major crystallographic challenge. Solution of the structure yielded a probable formula of HBi_3_Cu_12_I_22_(THT)_8_, which is nearly a tetramer of **3A**. Among the challenges associated with structure **3B** were a long axis (61 Å), pseudomerohedral twinning of the monoclinic *P*2 cell about a nearly 90° *b*-axis, nearly whole molecule disorder, and 45 independent atoms heavier than carbon. A top-down view of the sheet structure for **3B** is shown in Figure 6, showing only the major of the disordered positions. There are four structural motifs present: structural features *X* and *X′* represent face-sharing octahedral BiI_3_ stacks, with *X* being fully and *X′* half independent. The heavy atoms located in the structure yield the empirical formula of Bi_3_Cu_12_I_22_(THT)_8_, which has 22– charges, but only 21+ charges. Therefore, we speculate that a protonated site exists somewhere in the large crystallographically independent unit. As is the case for **3A**, all BiI_3_ stacks show alternating disordered Bi positions, which anchor the disordered substructures. Also, as is the case with **3A**, **3B** contains the 5-membered Cu_3_S_2_ rings (*Y*) that bridge between the *X′* and *X* features. The *Z* feature is the well-known (Cu_2_I_2_)_∞_ ladder. By way of contrast, the simpler **3A** structure shows only *X* and *Y* features. The sheets in **3B** show a zigzag ripple, bending sharply at the *X′* positions. Even though their Bi:Cu ratios are not quite identical, the chemical similarity of **3A** and **3B** rendered their independent synthesis highly unlikely and their chemical makeup nearly indistinguishable.

An overview of the four BiCu_3_I_6_(THT)_*n*_ structures, presented in Figures 3–6, shows similarities among them and to the known series of CuI-THT compounds.^50^ In all cases, Cu(I) and Bi(III) adopt T_d_ and O_h_ coordination environments, respectively. In all cases, THT is bonded only to the soft Cu(I) centers, while iodide centers form bridges between Bi and Cu. The principal SBU appears to be the THT-bridged three-copper cluster. In **1** and **2**, this takes the form of Cu_3_S_4_I_6_ and Cu_3_S_3_I_6_, respectively, while in **3A** (and **3B**), the Cu_3_S_2_I_6_ SBU requires μ_3_-iodides to fill in for the missing THT.

**Figure 7:**
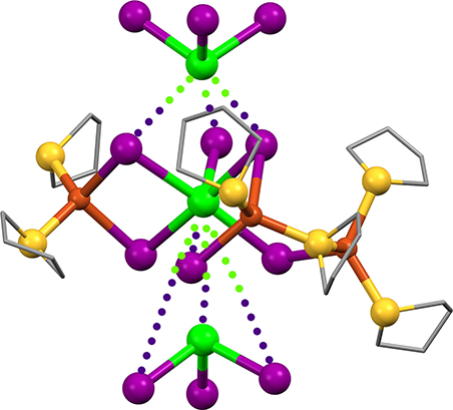
X-ray structure of compound **4**. Hydrogen atoms and disordered atoms omitted for clarity. Long interactions shown with dotted lines.

Moving to the Sb complexes, we see rather different structural forms. Compound **4** readily crystallizes as large red blocks from toluene/pentane and solves in monoclinic space group *P*2_1_/*n* as the only molecular species found in this study. All atoms are crystallographically independent. As shown in Figure 7, the molecule consists of a central Sb_3_I_12_ core that is decorated with Cu-THT about the central Sb via bridging iodides. The outer SbI_3_ units show very long Sb···I distances to the core iodides. The bond distances between the outer Sb atoms and their terminal iodides (Sb2 to I7, I8, I9; Sb3 to I10, I11, I12) range from 2.7607 to 2.8114 Å. Distances between these outer Sb atoms and the inner I1–I6 are much larger, ranging from 3.356 to 3.616 Å. Thus, the outer Sb2 and Sb3 form nearly independent SbI_3_ units, as indicated by the dotted connections in Figure 7. The Sb–I bond distances within the central core (Sb1 to I1–I6) are quite variable, ranging from 2.850 to 3.259 Å. The long Sb···I bonds result in some unusual bond angles. The angles about central Sb1 are within 4.5° of O_h_. In contrast, for Sb2 and Sb3 the I–Sb–I bond angles in the SbI_3_ unit are in the range of 93.14–98.55°, while the I···Sb···I angles associated with the I atoms that bridge to the central core are 72.63–79.37°. This constriction is presumably the result of the outer SbI_3_ units pulling away from the central O_h_. The Cu T_d_ are more distorted as well. The two S–Cu–S angles involving terminal THT ligands are 117.23 and 124.26°, being large presumably because no ring structure constrains them.

Given the formula of **4**, Sb_3_Cu_3_I_12_(THT)_6_, it would be reasonable to expect that there would be three CuI_2_(THT)_2_ units symmetrically distributed about the central core, with each sharing a pair of iodide atoms with the central SbI_6_ unit and all THT ligands terminal. However, this is decidedly not the case. Instead, only a single CuI_2_(THT)_2_ unit (Cu3) behaves this way, while Cu2 forms a CuI_2_(THT)_2_ unit with a terminal and a bridging THT. The latter bridges to Cu1, which participates in a CuI(THT)_3_ unit. The other two THTs on Cu1 are terminal. Since Cu1 binds only one iodide, core I6 does not bond to any Cu atom, but rather bonds only to Sb1, plus shows a long-range interaction with Sb3. Two-position disorder is present in a single THT methylene unit.

**Figure 8:**
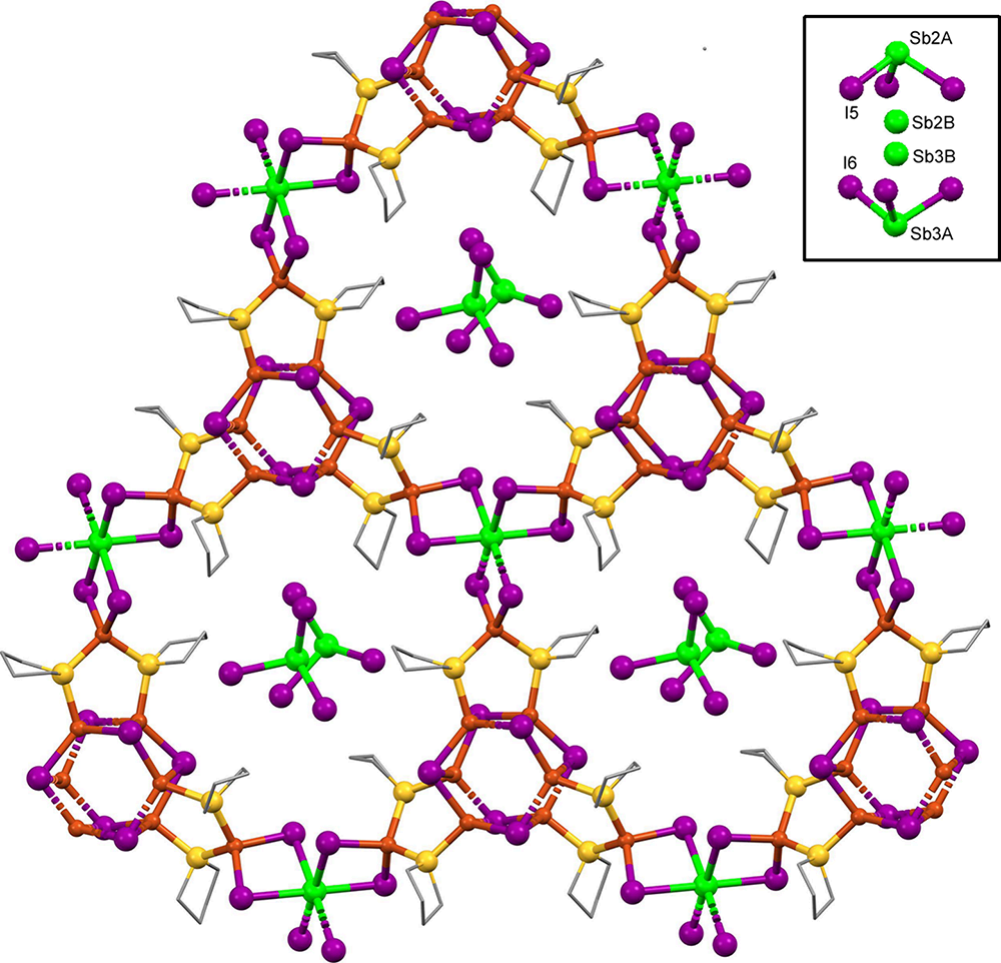
X-ray structure of compound **5**, viewed nearly along the *c*-axis. Hydrogen atoms and disordered atoms omitted for clarity. Inset: SbI_3_ stack showing disorder.

Orange prismatic crystals of **5** grown from hot toluene solved in the trigonal *R*3 space group with pseudomerohedral twinning present. The formula of compound **5**, SbCu_3_I_6_(THT)_2_, suggests an Sb congener of **3A**. However, while the two have some SBU aspects in common, **5** displays differences from Bi-containing **3A**. The structure of **5**, shown in Figure 8, is quite complex. As with **3A**, the 2-D sheet structure of **5** shows the Cu_3_S_2_I_6_ SBU that incorporates a Cu_2_I_2_ rhomb instead of a third bridging THT. However, symmetry-equivalent trios of these rhombs link together, forming Cu_6_I_6_ barrel-like clusters around a 3-fold center of symmetry. The barrels comprise two staggered Cu_3_S_3_ chair rings stacked along the *c*-axis, or alternatively can be seen as six edge-sharing Cu_2_I_2_ rhombs perpendicular to the *c*-axis. This cluster, albeit uncommon, has been seen previously.^60−62^ The barrels result in two different Cu_2_I_2_ rhombs, both comprising Cu2, Cu3, I3, and I4. One of these is nearly planar with deviations from least-squares plane of <0.05 Å, and a cuprophilic Cu2···Cu3 interaction of 2.626 Å. The other is bent with deviations from planarity around 0.17 Å and a longer nonbonding Cu2···Cu3 distance of 3.141 Å. As with **3A** and **3B**, the Cu atom not participating in the dimer (Cu1) coordinates two μ_2_-THT and two μ_2_-I that form a rhombic bridge to the 1/3-independent Sb1 atom. All the Cu_2_I_2_ and CuI_2_Sb rhombs are nearly planar. Bond angles around Sb1 are within 2.5° of O_h_, and those around nonbarrel Cu1 are within 4.5° of T_d_. Predictably, the barrel cluster produces more distortion in the Cu2 and Cu3 angles, with a range of 96.49–123.49°. The internal angles of the 5-membered Cu_3_S_2_ ring are 103.76, 110.41, and 111.00°.

Comparing **5** to **3A**, the additional CuI clustering into barrels in **5** produces a 67% deficiency of SbI_3_. Two other 1/3-independent SbI_3_ units (Sb2 and Sb3) lie inside the pores of the honeycomb formed by 3-barrel/3-SbI_6_ macrocycles (see Figure 8). Interestingly, these pyramidal SbI_3_ units are not directly bonded to the honeycomb. Instead, they lie in the pores within the sheets and above/below the barrels on alternate sheets, sharing the 3-fold crystallographic axis with these barrels. As is the case with **4**, I–Sb–I angles in these isolated SbI_3_ groups are in the 95–97° range, while the elongated interactions between the SbI_3_ and the barrel I atoms produce I···Sb···I angles of 75.96 and 77.10°. The positions of both Sb2 and Sb3 are disordered in roughly 1:1 fashion over two positions that represent an umbrella-flip of the SbI_3_ trigonal pyramid. The SbI_3_ units do not strongly link into face-sharing O_h_ stacks since the Sb–I distances within the SbI_3_ units are 2.763–2.779 Å, while the Sb–I distances between them are 3.594 and 3.619 Å. One THT molecule shows envelope-flip disorder.

As noted above, the neutral Sb–Cu compounds show structural differences from those of Bi–Cu. The Cu(I) centers present in all structures impose Cu_3_I_6_(THT)_*n*_ SBUs that are familiar from our previously reported study of CuI-THT compounds.^50^ The obvious structural differences are associated with the pnictogen atoms. In **1**–**3**, the bismuth(III) iodide forms only BiI_6_ O_h_ units. Additionally, BiI_3_ shows no tendency to segregate from the main network. On the other hand in both **4** and **5**, SbI_6_ O_h_ are accompanied by largely independent SbI_3_ units. These units show little to no interactions with one another.

### 3.4 Hirshfeld Analysis

**Figure 9:**
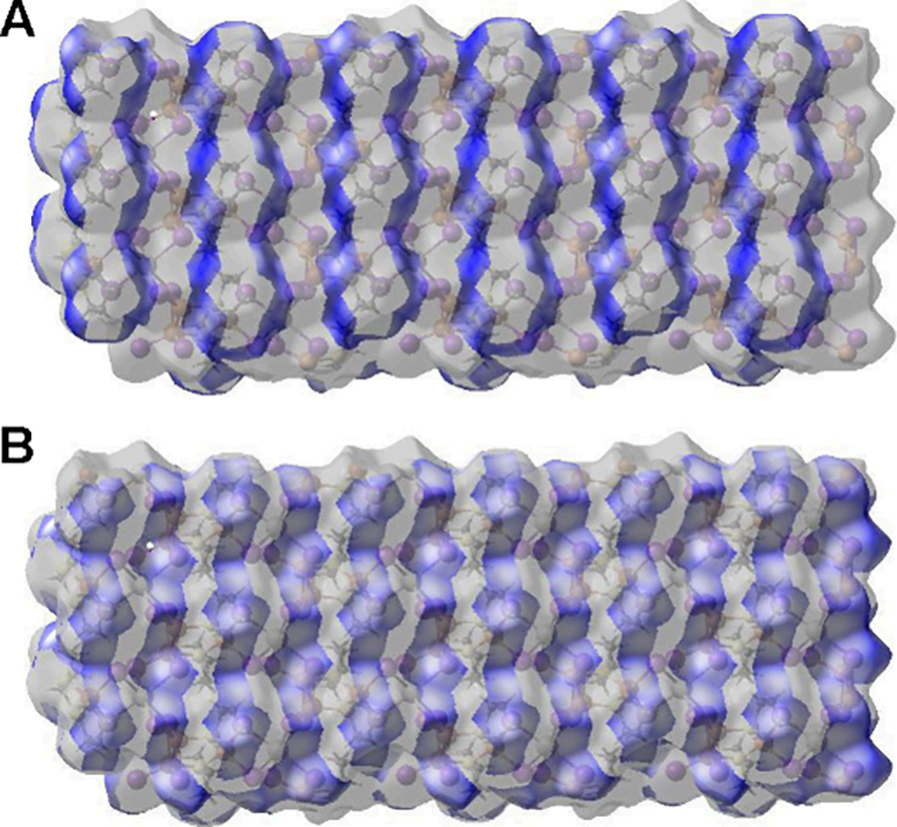
Hirshfeld surfaces for 2-D sheet in **3A**, viewed roughly down the *c*-axis with surface transparency. (A) H···H interactions. (B) H···I interactions.

The interactions between the 2-D sheets in **1**, **2**, **3A**, and **5** and molecular complex **4** were examined by Hirshfeld analysis, using the CrystalExplorer software.^58^ Finite truncation of sheet structures was carried out to ensure neutrality. In each case, crystallographic results show that the exteriors of the sheets in **1**, **2**, and **3A** are covered by hydrocarbon portions of THT. Thus, hydrogen atoms are the most surface-exposed atoms. Consistent with this observation, Hirshfeld analyses indicate the H atoms are involved in nearly all surface–surface interactions, interacting with other H or I atoms, see Table 3. Sheet truncation resulted in small amounts of invalid exposed sheet edges. These produced a collection of minor interactions (notably Bi···I, Cu···H, Cu···I, and Cu···S) that are artifacts of the modeling approach. These artificially imposed interactions are collected as “other” in Table 3. Careful analysis of the results shows that only H···H and I···H interactions are meaningful for **1**, **2**, and **3A**. The crystal structures reflect this behavior, revealing intersheet interactions in **1** (I2···H4A 3.057 Å, I3···H2B 3.292 Å, H2B···H7B 2.879 Å, H2B···H6A 2.452 Å, H2B···H7A 3.020 Å), **2** (I3···H10A 3.174 Å, H6B···H8B 2.272 Å, I5···H10B 3.042 Å, I2···H8A 3.280 Å), and **3A** (I2···H3A 3.585 Å, I4···H1B 3.524 Å, H4A···H4A 2.615 Å). Hirshfeld surfaces for these interactions in **3A** are shown in Figure 9. Bands of H···H and I···H interactions can be seen running along the *a*-axis and alternating along the *b*-axis. These bands reflect the alternating rows of THT and BiI_6_ units in the structure, see Figure 5. Other interaction surfaces and fingerprint plots can be found in the SI (Figures S34–S38).

**Table 3:** Selected Close Atom–Atom Contacts with Corresponding Contribution (%)^a^

	**1**	**2**	**3A**	**4**	**5**
I···H	30.6	40.8	55.4	49.7	50.9
H···H	58.4	46.5	31.4	39.8	32.4
other^a^	11.0	12.4	13.2	10.7	16.7

a Other interactions for sheets **1**, **2**, **3A**, and **5** include Bi/Sb···I Cu···H, Cu···I, and Cu···S that are artifacts of imposed sheet edges. For molecular **4**, all interactions are valid and other includes 4.3% S···H, 3.2% S···I, and 2.5% I···I. For **5**, valid Sb···I interactions linking the sheets amount to 4.0%.

Turning to the Sb complexes, molecular compound **4**, shows similar intermolecular noncovalent interactions to those in **1**–**3A**, i.e., dominated by H···H and I···H interactions, but also shows other minor interactions that, by necessity, are not artifacts of sheet truncation. Compound **5** shows intersheet interactions like those in the Bi sheets (I2···H3A 3.676 Å, I2···H3B 3.481 Å, I2···H4A 3.184 Å, H6A2···H4A 2.524 Å, H6A2···H4B 2.612 Å). However, it is distinguished from **1**–**3A** by having semi-independent SbI_3_ groups. These show their own I···Sb interactions (I5···H7A2 3.098 Å, I6···H8A2 3.148 Å) as well as interaction with the iodine atoms in the Cu_6_I_6_ barrels (Sb2A···I1 3.519 Å, Sb3A···I4 3.468 Å). These Sb···I interaction account for 4.0% of the total in the Hirshfeld surface in **5**.

### 3.5 Metal–Ligand Bonding

A QTAIM analysis was performed on **1**, **2**, **3A**, **4**, and **5** to determine the nature of the metal-halide bonding and to compare that of the Bi compounds to the lighter Sb analogs. Tables of QTAIM parameters can be found in the SI. Taking the electron density (ρ) as the bond “strength” the metal-halide bonds generally weaken with increasing row Cu > Sb > Bi and with increased bridging μ_1_-I (terminal) > μ_2_-I > μ_3_-I > μ_4_-I. Metal-halide bonding can be considered mostly ionic with values of ρ < 1 and the Laplacian (∇^2^ρ) > 0.^63^ The ionic nature is most pronounced in the Sb containing compounds **4** and **5** where the Sb–I bonds have the smallest ∇^2^ρ values, typically half the value of the Bi compounds **1**–**3A**. Notably, the potential and kinetic ratios (|V|/G) of the Sb–I bonds (1.09 to 1.75) are generally larger and have greater variability compared to the Bi–I bonds (1.23 to 1.34). These larger |V|/G values point to increased Sb/I orbital mixing compared to Bi/I. Overall, the metal-halide bonding is mostly ionic in all cases and only minor variations are realized between Sb and Bi analogs.

**Figure 10:**
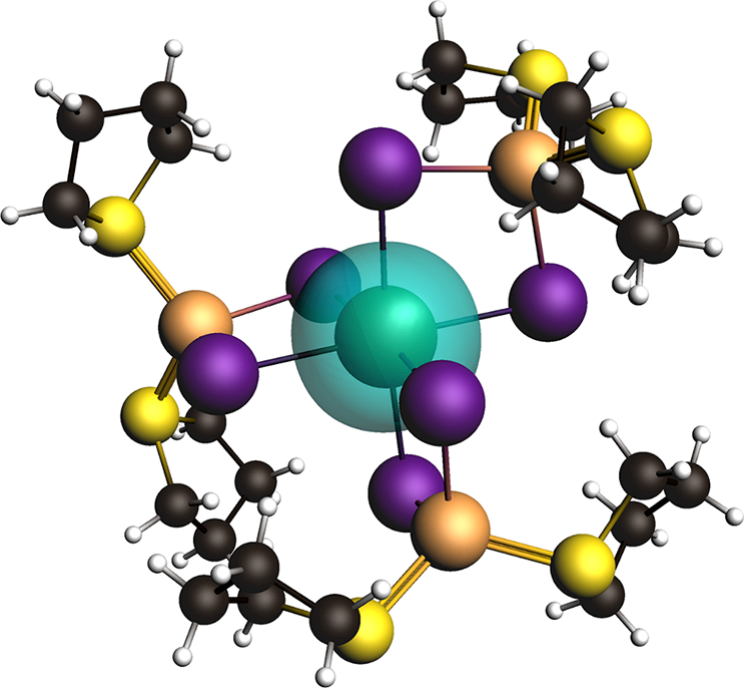
Representative NLMO (±0.03 isosurfaces) calculated for **1** showing the stereochemical inactive Bi 6s orbital.

**Figure 11:**
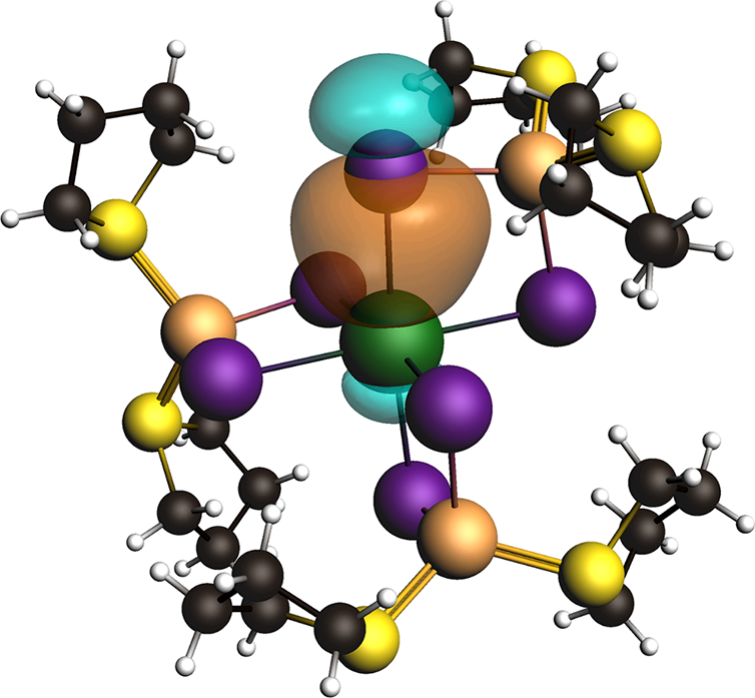
Representative NLMO (±0.03 isosurfaces) calculated for **1** showing the bonding Bi–I σ via interaction of Bi 6p and I 5p orbitals.

An NLMO analysis was performed to further understand the metal-halide bonding and orbitals involved. As indicated by the structural analysis and illustrated for **1** in Figure 10, the Bi and Sb s orbitals are stereochemically inactive in all compounds and do not interact with adjacent I orbitals. For the Bi-based compounds, there is little variation in the Bi–I bonding. Here, the Bi 6p orbitals participate in bonding with those of the I 5s and 5p orbitals, which are illustrated in Figure 11. The bridging halide atoms in **1**–**3A** themselves do not undergo significant hybridization, retaining a >70% s character and >90% p character. In the Sb compounds **4** and **5**, the terminal I and bridging μ_2_-I orbitals also remain unhybridized; however, this behavior deviates for some μ_3_-I and all μ_4_-I atoms. Using I74 in **4** as a representative example, this μ_3_ (Sb)_2_–I–Cu bridging halide features one lone electron pair within an sp orbital, an electron pair in a weakly bridging Sb–I–Sb sp^3^ orbital, and two electron pairs within two p orbitals that bond with the Cu and Sb metals. In all cases involving Sb bridging halides, an unhybridized p orbital is involved in the metal bond, leaving the lone pair electrons to occupy the remaining s and p orbitals. It is of note that this hybridization is unique to the Sb compounds reported herein and completely absent from the Bi compounds. Whether this behavior arises strictly from the bonding motifs unique to the Sb compounds or as a result from some direct subtle influence from the neighboring atom (e.g., heavy atom effect) is unclear. Finally, we point out evidence of weak delocalization of electrons across the metal-halide bonds. For example, in **1**, the Bi–I81 bond is delocalized with the *trans*-I78 atom, which contributes a modest 2.98% of the electron occupancy. This behavior is not unique to the Bi compounds as similar delocalization is seen in **5**, for example, across the I65–Sb89–I69 bonds.

### 3.6 Photophysical Behavior

**Figure 12:**
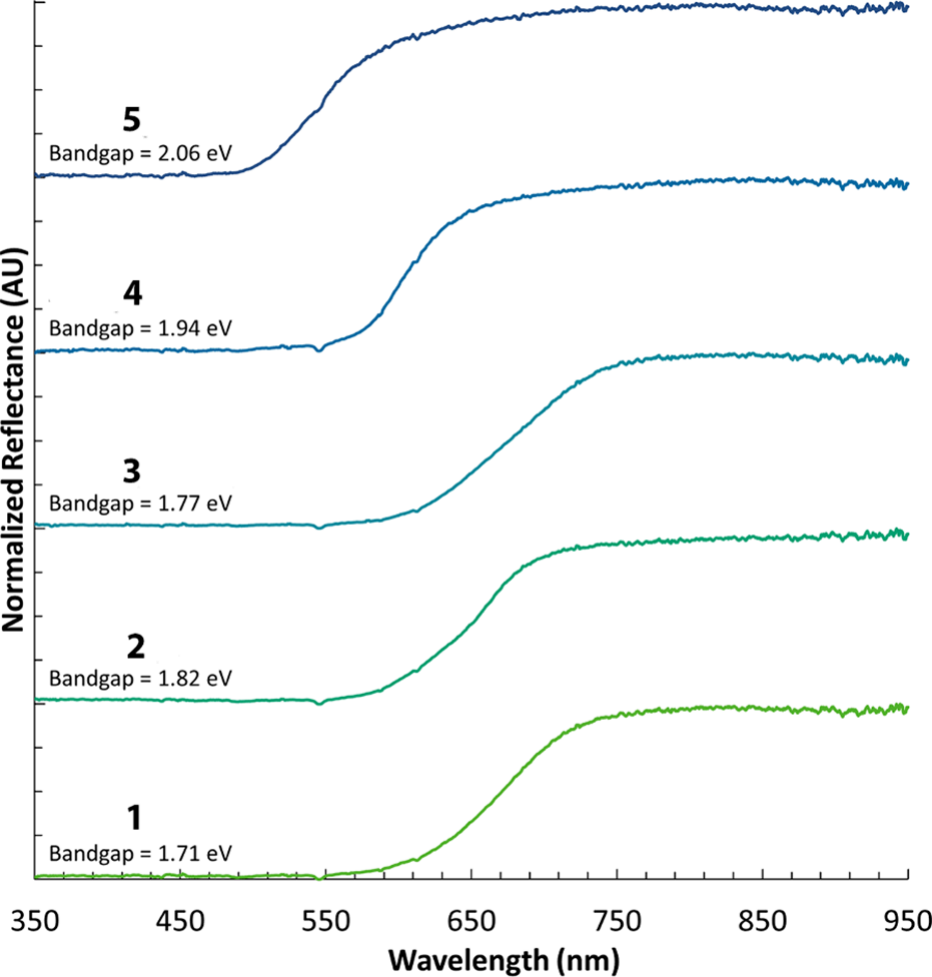
Diffuse reflectance spectra of **1**–**5** collected at 298 K.

Compounds **1**–**5** (**3** being a mixture of **3A** and **3B**) are powders with colors ranging from deep red (Bi) to orange (Sb). Diffuse reflectance measurements were performed to quantify the color differences and determine the optical properties. As shown in Figure 12, all compounds feature a broad absorption band that ranges from the UV to the lower energy visible range. In the Bi^3+^-based compounds **1**, **2**, and **3**, this band completely terminates between 660 and 750 nm. For the Sb^3+^ compounds **4** and **5**, this band only extends to 570 and 640 nm, respectively. Optical band gaps were determined showing semiconductive energy values ranging from 2.06 eV (**5**) to 1.71 eV (**1**). The origin of this band is difficult to determine given the presence of multiple potential electronic pathways, e.g., I → Bi/Sb, I → Cu, THT → Cu/I. For example, (CuI)_*x*_(THT)_*y*_ compounds are known to be emissive most likely owing to a cluster centered or ligand to cluster transition,^50^ while bismuth and antimony halometallates are known to undergo a halide to metal transition.^6,28−32^ The lack of π* acceptor orbitals in the THT ligand precludes the possibility of a metal or metal/halide to ligand charge transfer; however, the lone electron pair on the S permits a ligand to metal/halide transition.

**Figure 13:**
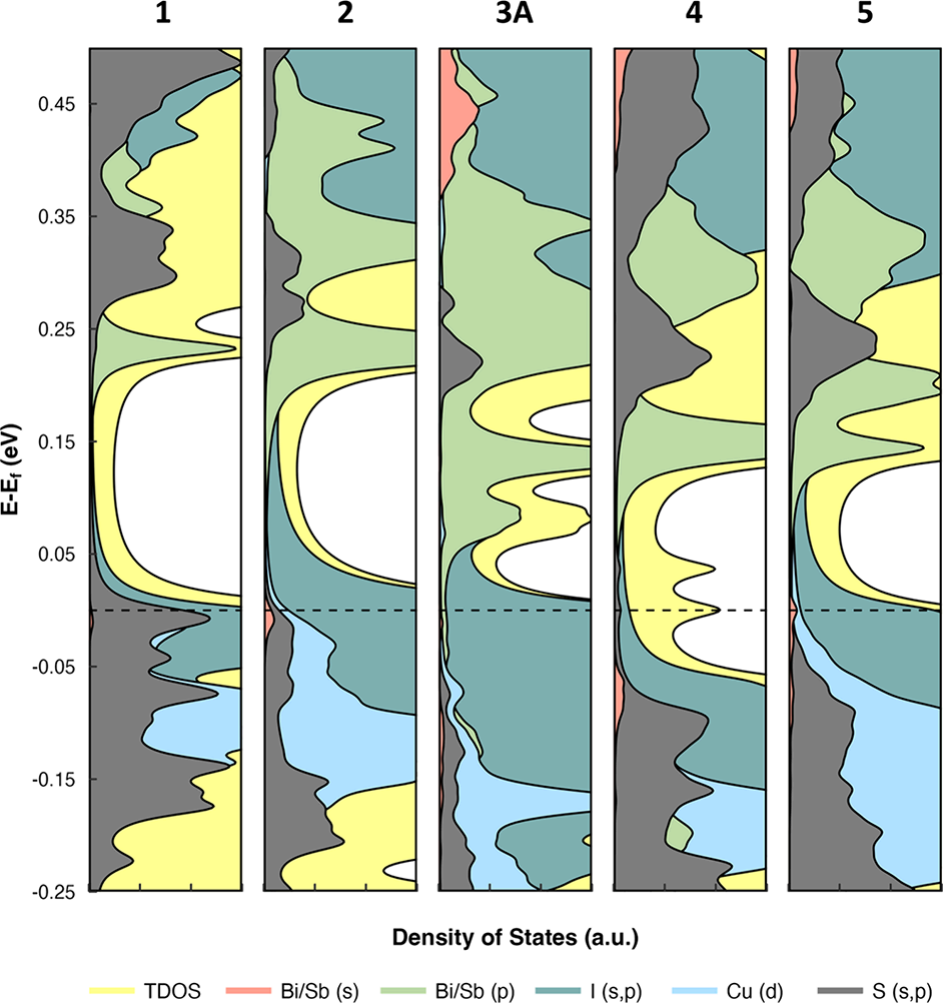
Total and partial density of states of **1**–**5** projected onto atomic orbitals of Sb/Bi, I, Cu, and S. The *E*_Fermi_ (*E*_f_, HOMO) is highlighted by a dashed line.

As such, to rationalize the optical properties observed herein, we have turned to DFT calculated partial density of states (DOS) to map the electronic structures of **1**, **2**, **3A**, **4**, and **5** (Figure 13) based on the models built from crystallographic subunits used for NLMO and QTAIM analyses. Generally, the lower lying empty molecular orbitals are primarily composed of the Bi/Sb p shells, energetically lying below the S s and p shells. Only compound **4** is an exception, showing energetically low empty S/C s and p shells (THT); however, the Sb p shells are energetically directly above and similar in energy to the other compounds. The higher lying filled molecular orbitals are more varied from compound to compound. In compounds **2**, **3A**, and **5** significant contributions by the I s and p shells are observed, while some Bi/Sb s character is present in **2** and **5**. For **1** and **4**, these high-lying filled molecular orbitals show significant I–Cu–THT and THT character, respectively.

**Figure 14:**
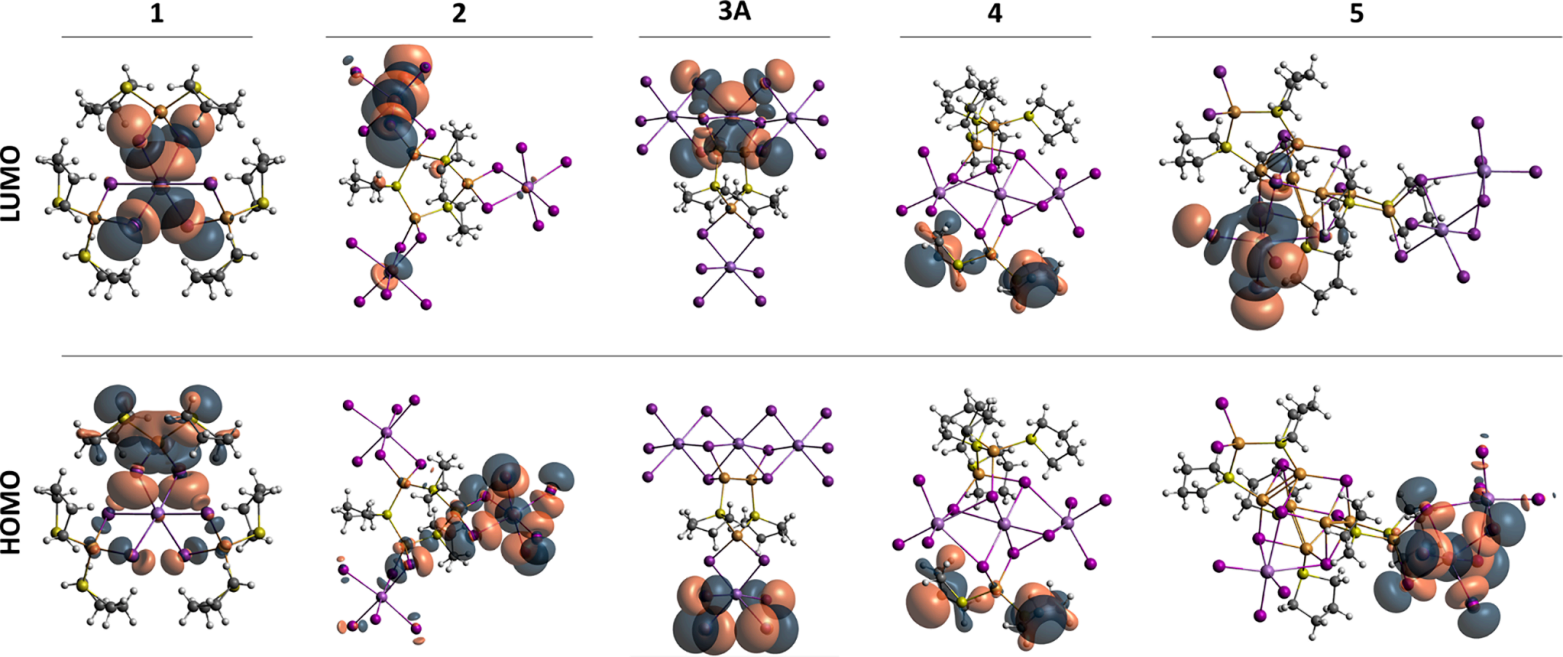
Frontier molecular orbitals (±0.02 isosurfaces) calculated for **1**–**5**.

The DOS findings are supported by renderings of the highest molecular orbital (HOMO) and the lowest molecular orbital (LUMO) in Figure 14. Here, **2**, **3A**, and **5** show Sb/Bi–I σ* orbital character in the HOMO and LUMO, while for **1** the HOMO is composed of Cu–THT σ and Cu–I σ orbitals and the LUMO is composed of Sb and I orbitals. We note that only compound **4** features predominant THT character for both the HOMO and LUMO. However, the overall intensity of these states within the total DOS is minor and indicates that this is a perhaps localized electronic substructure. It is not until HOMO–3 that the Sb s and I s and p shells are observed (see the SI). Overall, the DOS and MO findings generally indicate that the electronic transitions in **1**–**5** may be described as a type of halide to metal-halide rearrangement where electrons in the I s and p shells (or I–Cu-THT network) are populated into empty low lying Bi/Sb–I σ* orbitals. Interestingly, substitution of Bi for Sb does not appear to significantly change the orbital constructs. Given the population of Bi–I and Sb–I antibonding orbitals, we would expect a general destabilization of these bonds and thus weakening of the metal-halide network overall. This may have an important impact for compounds subjected to prolonged UV light exposure.

## 4 Conclusions

We have demonstrated that THT is an excellent ligand for producing ternary compounds of BiI_3_ and SbI_3_ with CuI. In all, six different compounds (**1**, **2**, **3A**, **3B**, **4**, and **5**) were produced, of which all but **4** are 2D sheet networks. A remarkable flexibility exists in the BiCu_3_I_6_(THT)_*n*_ system (**1**, **2**, **3A**), in which *n* values of 4, 3, or 2 are found. As the amount of THT is lessened in these compounds, the coordination number of the remaining THT and iodide ligands increases, but the structural motifs remain relatively consistent. The Sb species are quite distinct from those of Bi, despite corresponding formulas in the case of **3A** and **5**. The structures of both molecular **4** and networked **5** contain semi-independent SbI_3_ units. Hirshfeld analysis of **1**, **2**, **3A**, **4**, and **5** showed the preponderance of surface contacts to be either I···H or H···H in nature. Diffuse reflectance measurements suggest small band gaps in the range of 1.71–2.06 eV for these compounds, with the red Bi^3+^ species having lower values than those of Sb^3+^. Here, electronic mapping via DOS calculations and rendering of the frontier molecular orbitals indicates that the excited states involve Bi/Sb–I antibonding orbitals, although the origin of the transition varies between compounds. In general, the electronic transition is largely unaffected by the substitution of Bi for Sb, although more work needs to be performed. Additionally, we are very interested in pursuing additional Sb/I compounds given the unique hybridization observed in the NLMO calculations. We suspect that these changes in orbital construction may potentially be leveraged to give rise to unique chemical and electronic properties. It is clear, however, that Bi/I compounds are resistant to changes in orbital construct despite changes in bonding motifs.

## Supporting Information Available

Crystallographic renderings, IR traces, TGA traces, powder diffraction, and Hirshfeld fingerprints and surfaces (PDF)
