# Supplementary material for: Ternary Complexes of BiI3/CuI and SbI3/CuI with Tetrahydrothiophene
Source: Inorg Chem. 2024 Jun 8;63(25):11688–99. doi: 10.1021/acs.inorgchem.4c01147 (PMC11200257; doi:10.1021/acs.inorgchem.4c01147)

Supporting Material for

Ternary Complexes of BiI_3/CuI and SbI_3/CuI with Tetrahydrothiophene

James H. Ballenger,[a] Katherine S. Giunta,[a] Ruby Carlson,[a] Aaron D. Nicholas,[b] Lucas C. Ducati,[c] Marcos O. Oliveira de Brito,[c] Matthias Zeller,[d] Robert D. Pike[a]*

[a]*Department of Chemistry, College of William and Mary, Williamsburg, VA 23187-8795. Tel. 757-221-2555; E-mail: rdpike@wm.edu.*

[b] *National Security Directorate, Pacific Northwest National Laboratory, Richland, WA 99354. Tel: 509-375-2752; E-mail: aaron.nicholas@pnnl.gov.*

[c]*Institute of Chemistry, Universidade São Paulo, São Paulo, SP, 05508-220, Brazil.*

[d]*Department of Chemistry, Purdue University, West Lafayette, Indiana 47907-2084.*

Corresponding Author: Robert D. Pike

Department of Chemistry

College of William and Mary

Williamsburg, VA 23187-8795.

telephone: 757-221-2555

FAX: 757-221-2715

email: rdpike@ wm.edu

Contents

Figure S1. ORTEP diagram of the crystallographically independent unit for **1**………. 5

Figure S2. Polyhedral projection of **1**……………………………………..……….….. 6

Figure S3. Packing diagram for **1**………………………………….……..…………… 7

Figure S4. ORTEP diagram of the crystallographically independent unit for **2**………. 8

Figure S5. Polyhedral projection of **2**……………………………………..……….….. 9

Figure S6. Packing diagram for **2**………………………………….……..…………… 10

Figure S7. ORTEP diagram of the crystallographically independent unit for **3A**…….. 11

Figure S8. Polyhedral projection of **3A**…………………………………..……….….. 12

Figure S9. Packing diagram for **3A**……………………………….……..……….……. 13

Figure S10. ORTEP diagrams of the crystallographically independent unit for **3B**…... 14

Figure S11. Polyhedral projection of **3B**………………………………..……….……. 15

Figure S12. Packing diagram for **3B**……………………………….……..…….………16

Figure S13. ORTEP diagram of the crystallographically independent unit for **4**………17

Figure S14. Polyhedral projection of **4**…………………………………..……….…… 18

Figure S15. Packing diagram for **4**……………………………….……..……….……. 19

Figure S16. ORTEP diagram of the crystallographically independent unit for **5**………20

Figure S17. Polyhedral projection of **5**…………………………………..……….…… 21

Figure S18. Packing diagram for **5**……………………………….……..………….….. 22

Figure S19. IR Spectrum for **1**…………………………..……….……..……….……... 23

Figure S20. IR Spectrum for **2**…………………………..……….……..……….……... 24

Figure S21. IR Spectrum for **3**…………………………..……….……..……….……... 25

Figure S22. IR Spectrum for **4**…………………………..……….……..……….……... 26

Figure S23. IR Spectrum for **5**…………………………..……….……..…….….…... 27

Figure S24. Thermogravimetric analysis for **1** ..…………………………….……….….…. 28

Figure S25. Thermogravimetric analysis for **2** ..……………..…………….…….…..…... 29

Figure S26. Thermogravimetric analysis for **3** ..…………………………….……….……... 30

Figure S27. Thermogravimetric analysis for **4** ..………………………….…….………….. 31

Figure S28. Thermogravimetric analysis for **5** ..……………………….……….…….…….. 32

Figure S29. Powder diffraction for **1** ..…………………………………………….………… 33

Figure S30. Powder diffraction for **2** ..…………………………………………….………… 34

Figure S31. Powder diffraction for **3** ..…………………………………………….………… 35

Figure S32. Powder diffraction for **4** ..…………………………………………….………… 36

Figure S33. Powder diffraction for **5** ..…………………………………………….………… 37

Figure S34. Hirshfeld fingerprint graphs and surfaces for **1** ..…………………….………… 38

Figure S35. Hirshfeld fingerprint graphs and surfaces for **2** ..…………………….………… 39

Figure S36. Hirshfeld fingerprint graphs and surfaces for **3A** ………….….……….….. 40

Figure S37. Hirshfeld fingerprint graphs and surfaces for **4** ..…………………….…….……41

Figure S38. Hirshfeld fingerprint graphs and surfaces for **5** ..…………….………….…... 42

Figure S39. Truncated model of **1** used in DFT and QTAIM calculations.…………….... 43

Figure S40. Truncated model of **2** used in DFT and QTAIM calculations.……………. 44

Figure S41. Truncated model of **3A** used in DFT and QTAIM calculations.………….. 45

Figure S42. Truncated model of **4** used in DFT and QTAIM calculations.…………….... 46

Figure S43. Truncated model of **5** used in DFT and QTAIM calculations.……...…….. 47

Figure S44. Tauc Plots of compounds **1-5**…………………………………...……...…….. 48

Table S1. Summary of QTAIM parameters of **1-5**.……...…………………….…….…... 50

Figure 45. Rendering of the HOMO-3 for **4**..……………....……………………….….…..…... 51

For computational data for compounds **1**–**5** see https://zenodo.org/records/11177459.

Figure S1. ORTEP diagram of the crystallographically independent unit for **1**. Ellipsoids at 50%.

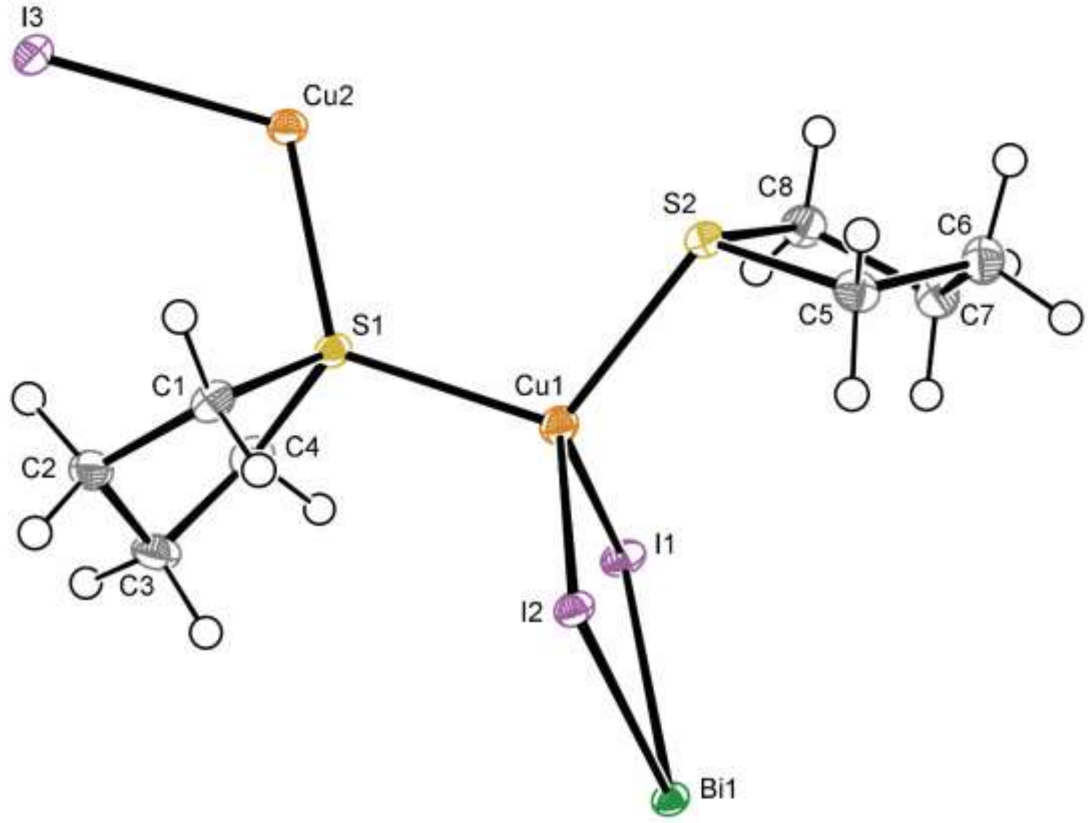

Figure S2. Polyhedral projection of **1**, hydrogen atoms omitted.

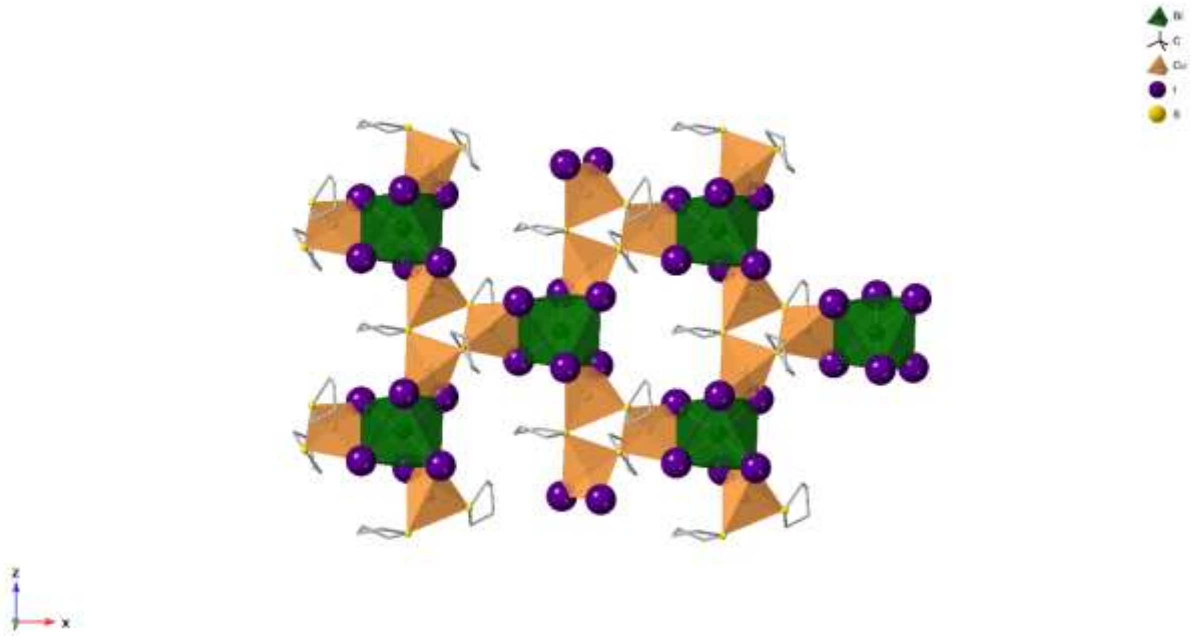

Figure S3. Packing diagram for **1**, viewed roughly along the *a*-axis. H atoms omitted.

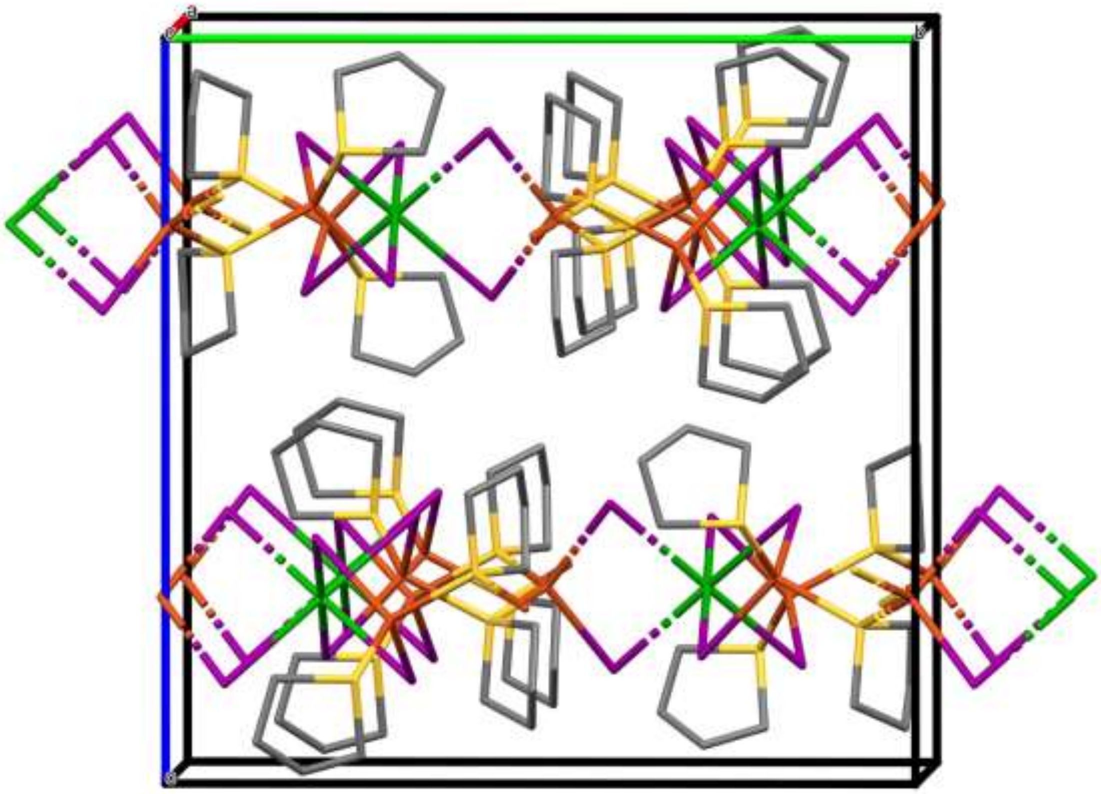

Figure S4. ORTEP diagram of the crystallographically independent unit for **2**. Ellipsoids at 50%.

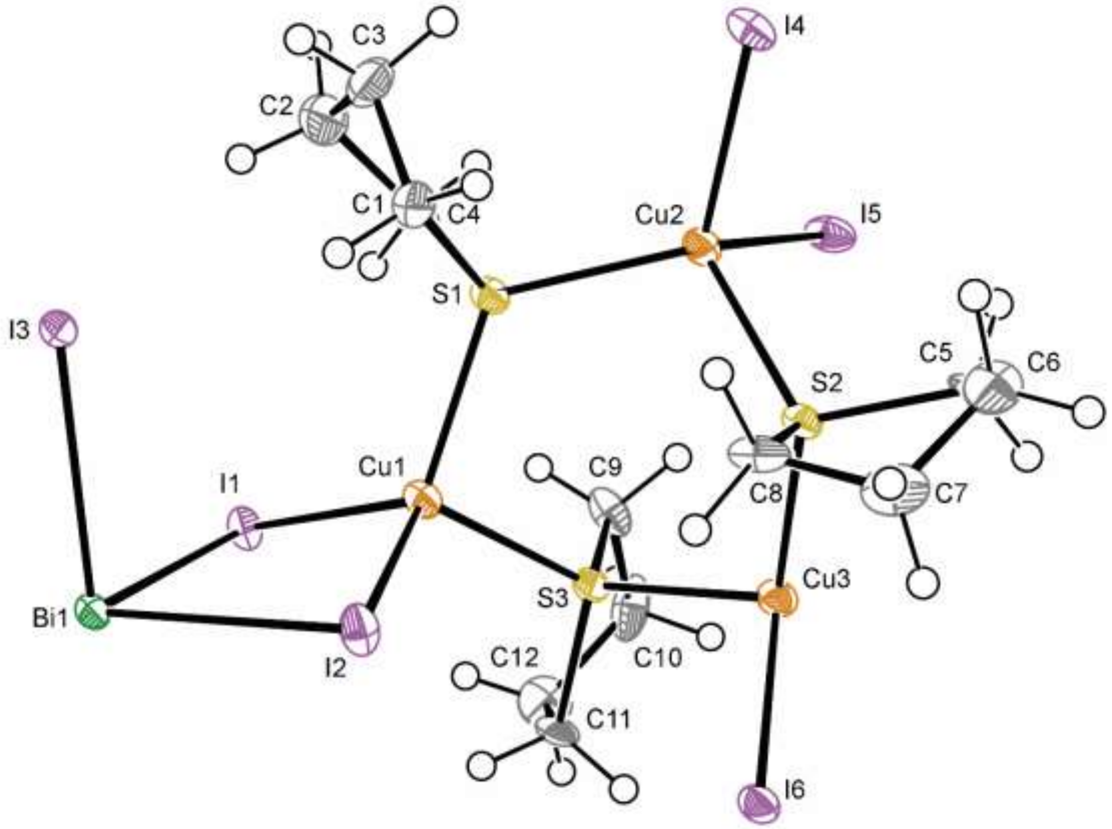

Figure S5. Polyhedral projection of **2**, hydrogen atoms omitted.

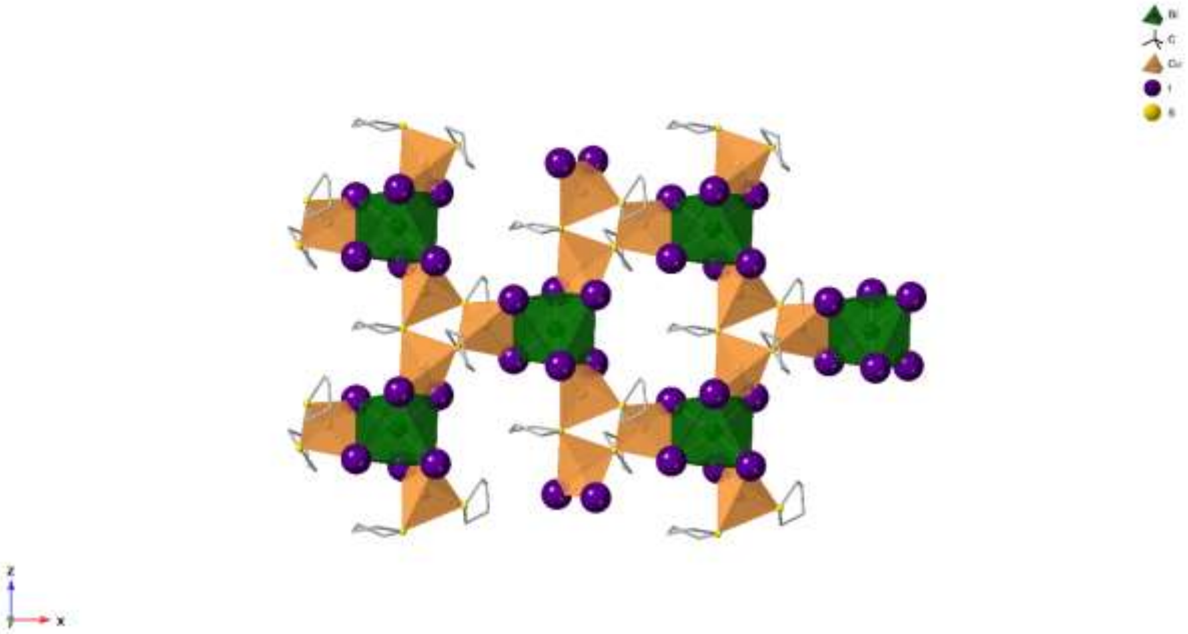

Figure S6. Packing diagram for **2**, viewed roughly along the *b*-axis. H atoms omitted.

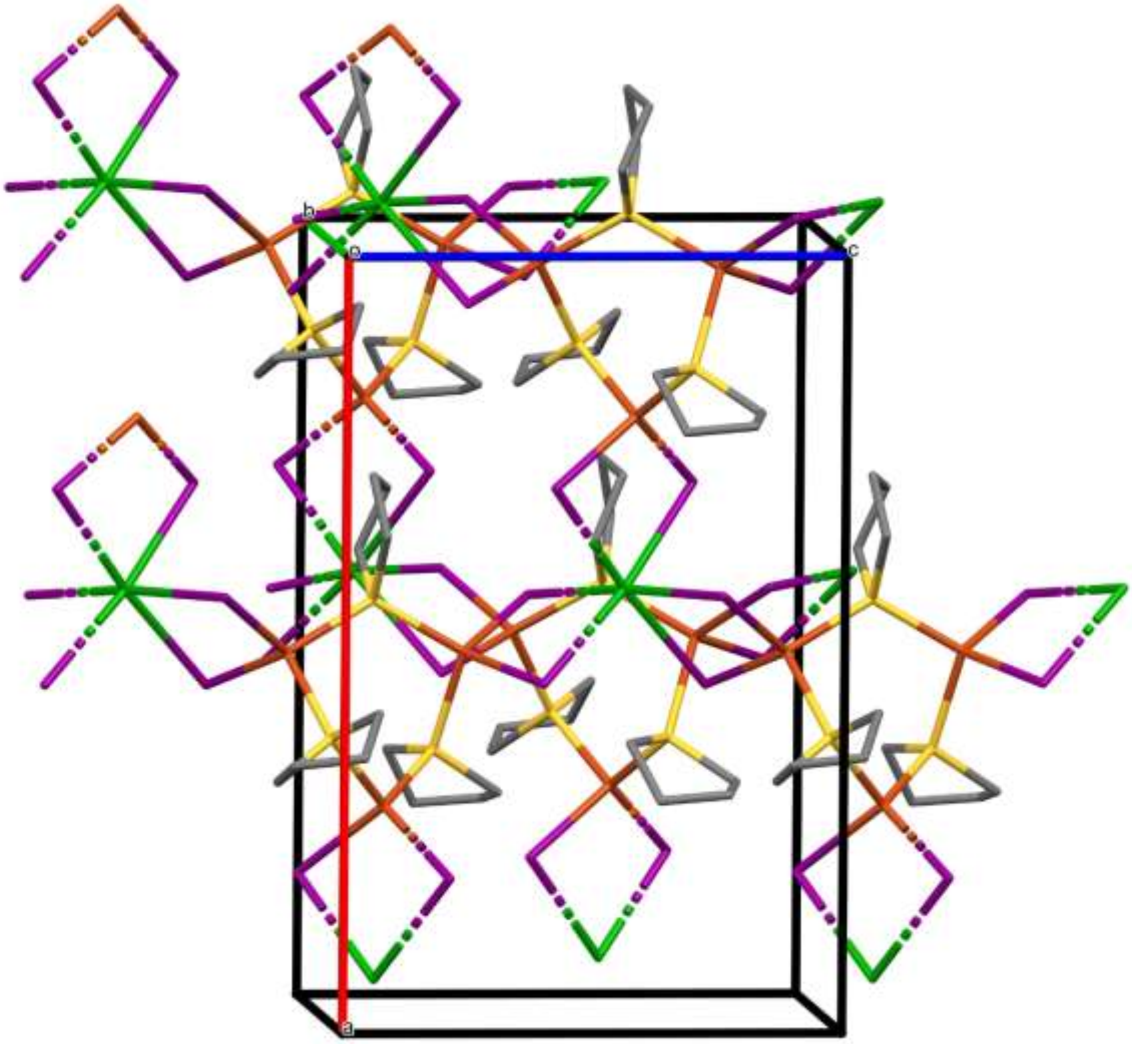

Figure S7. ORTEP diagram of crystallographically independent unit for **3A** showing Bi site disorder. Ellipsoids at 50%.

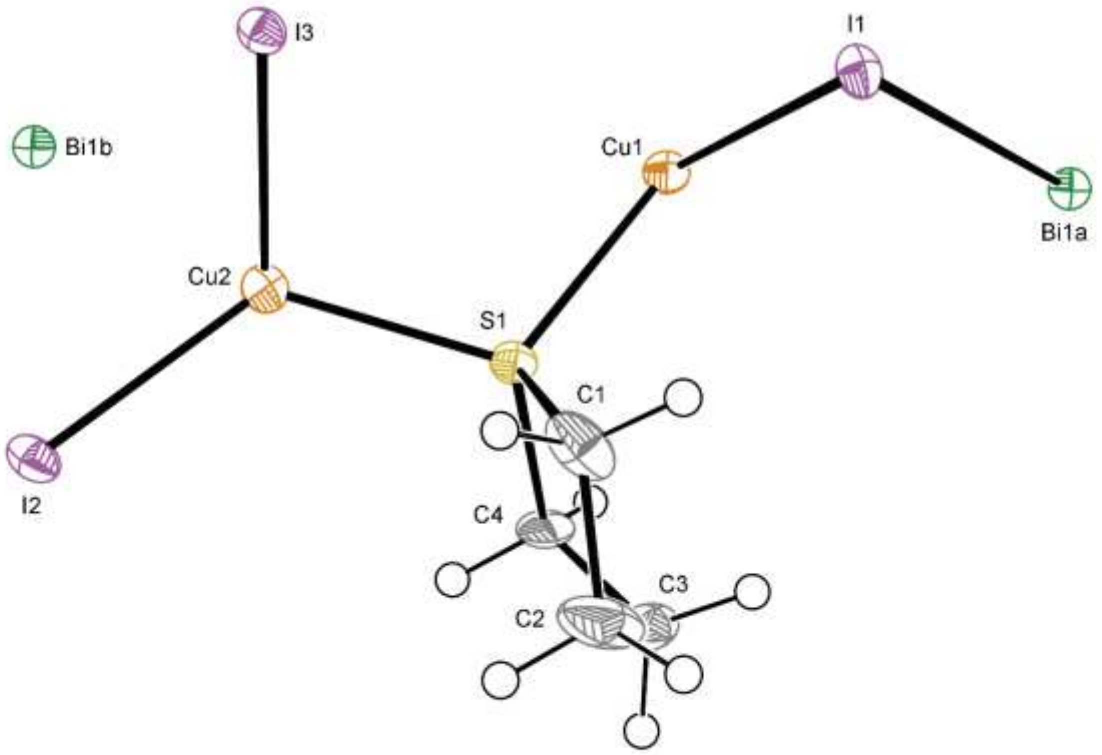

Figure S8. Polyhedral projection of **3A**, hydrogen atoms omitted.

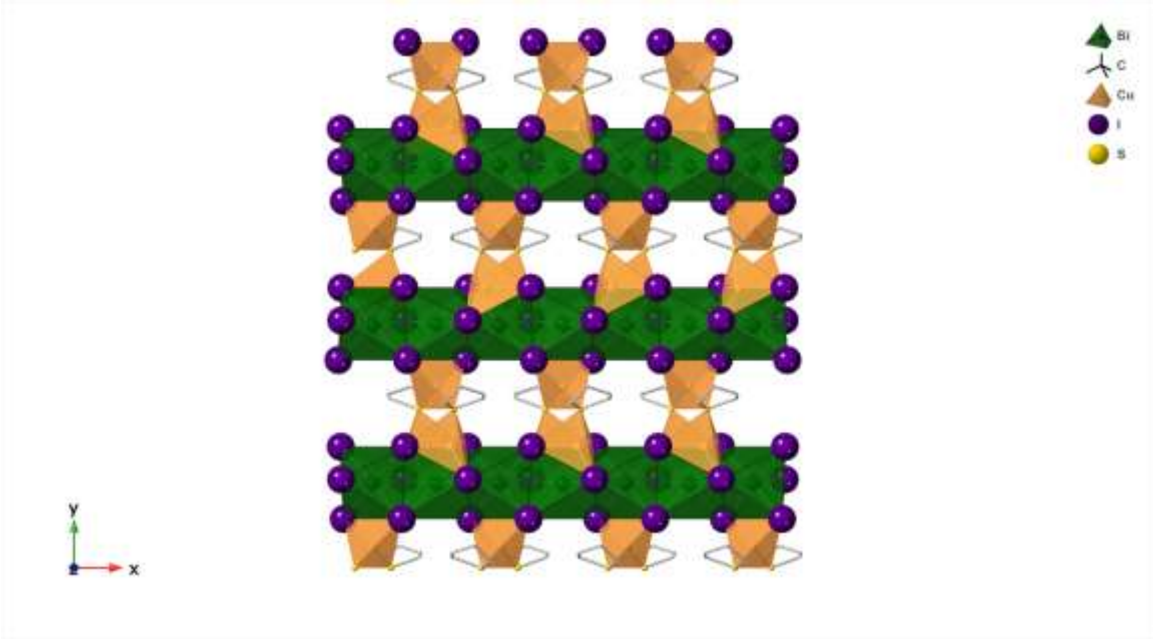

Figure S9. Packing diagram for **3A**, viewed roughly along the *a*-axis. Minor Bi and H atoms omitted.

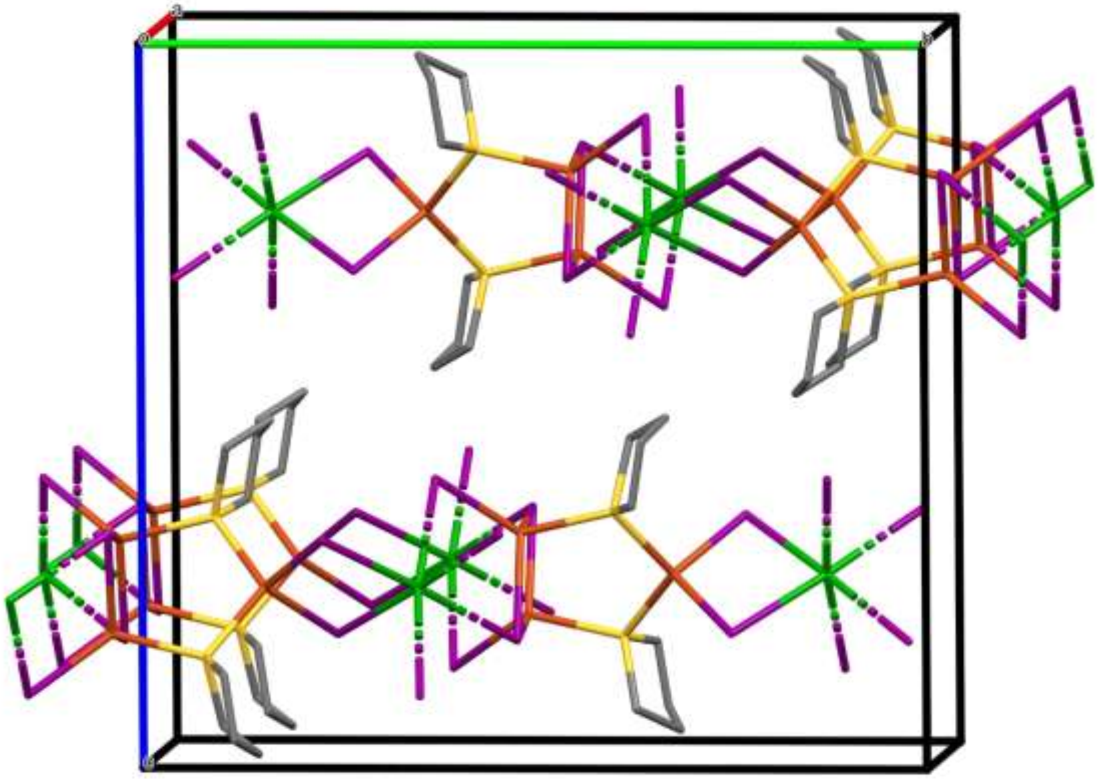

Figure S10. Top: ORTEP diagram of crystallographically independent unit for **3B** showing major disordered position only. H atoms omitted and carbons not labelled. Ellipsoids at 50%. Bottom: ORTEP diagram of crystallographically independent unit for **3B** showing disorder. Note that carbon and hydrogen atoms were not located for the minor position (shown in orange).

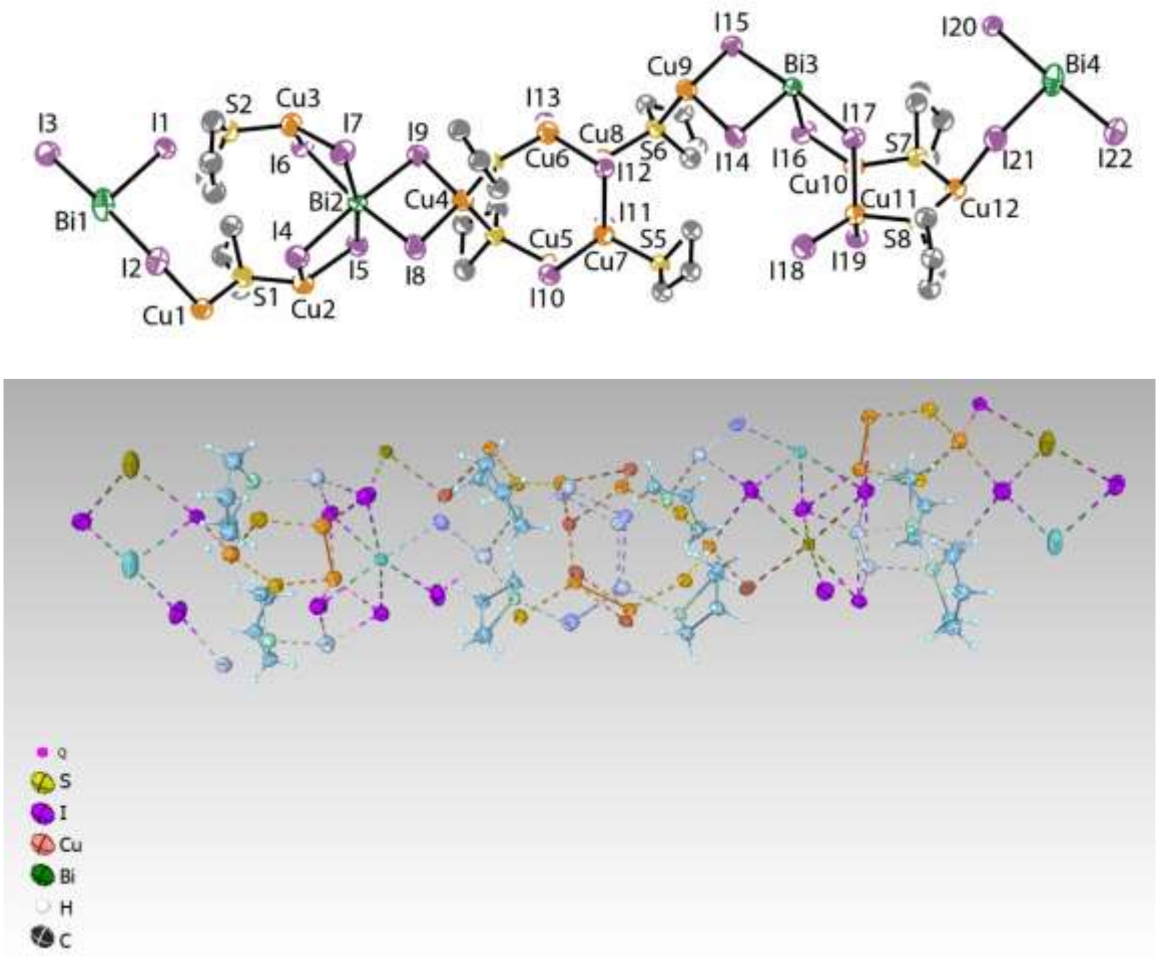

Figure S11. Polyhedral projection of **3B**, disordered atoms and hydrogen atoms omitted.

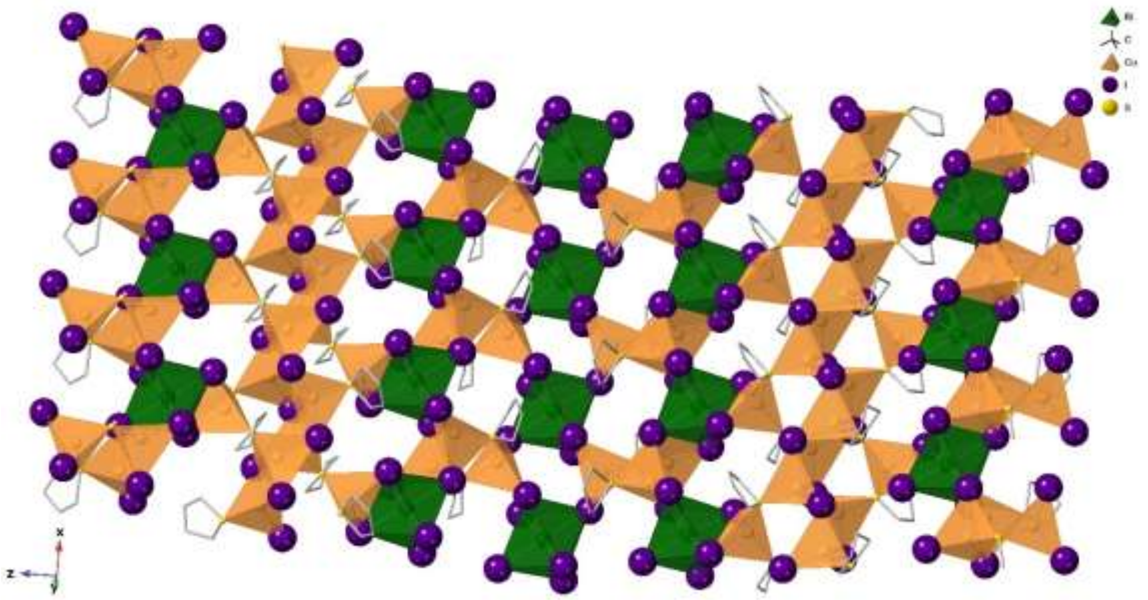

Figure S12. Packing diagram for **3B**, viewed roughly along the *a*-axis. H atoms and disordered atoms omitted.

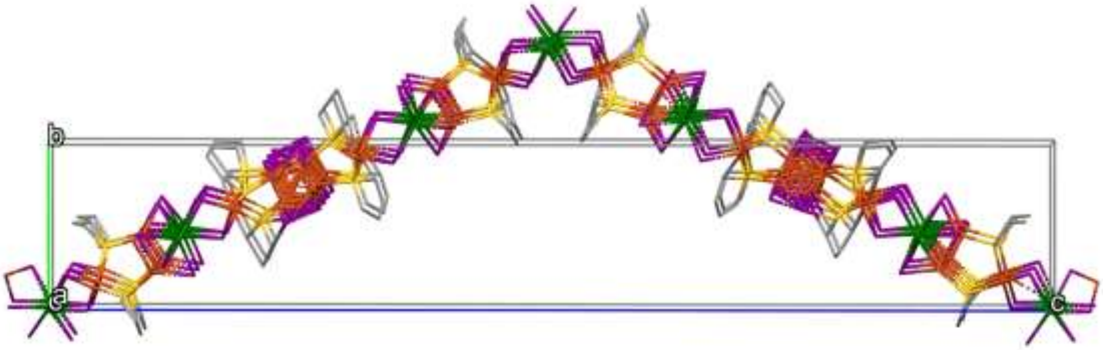

Figure S13. ORTEP diagram of crystallographically independent unit for **4** showing THT disorder. H atoms omitted. Ellipsoids at 50%.

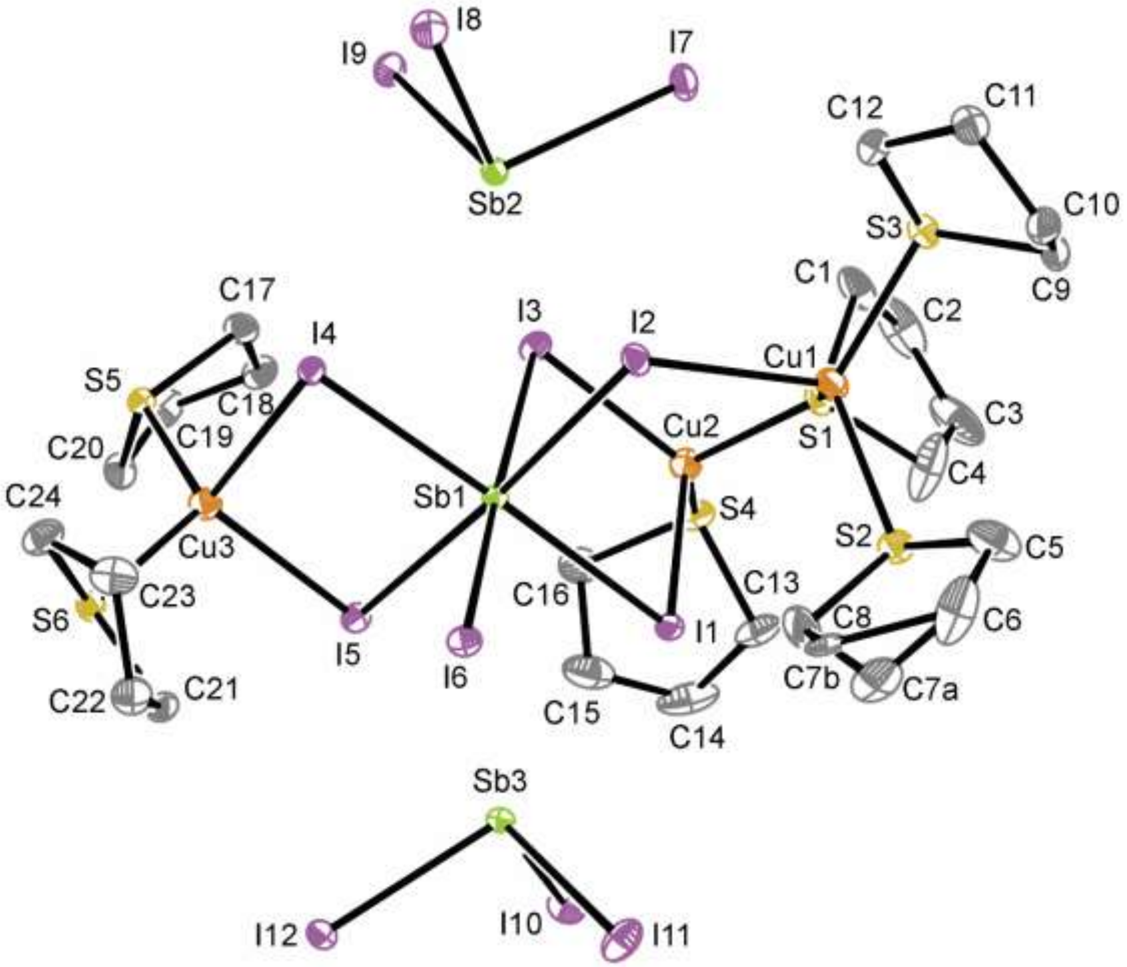

Figure S14. Polyhedral projection of **4**, hydrogen atoms omitted.

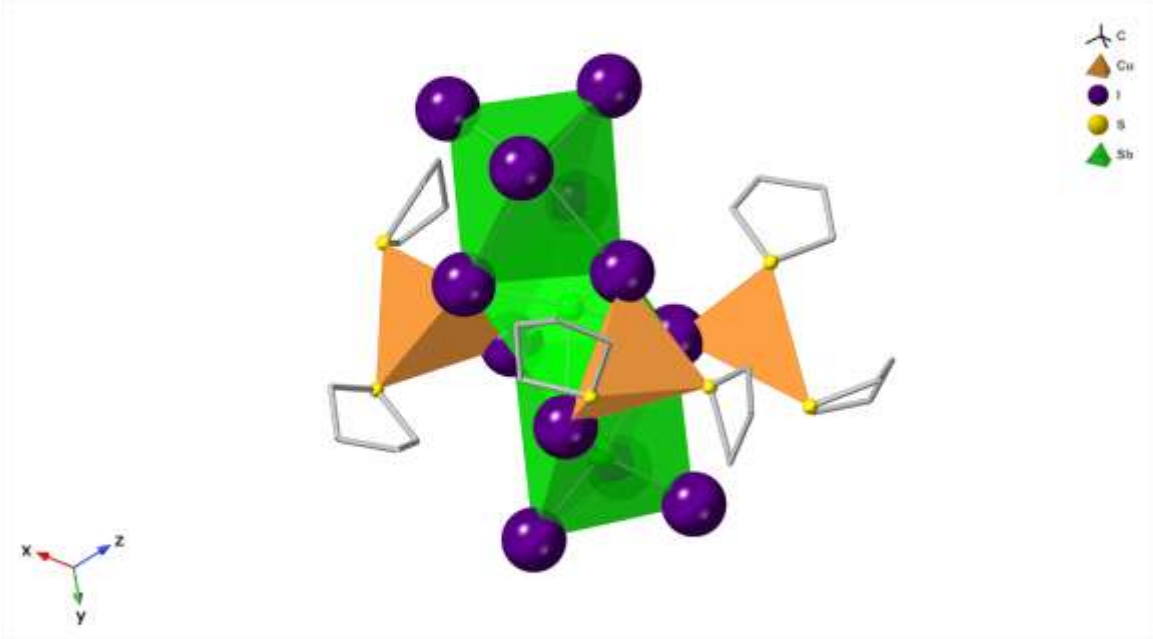

Figure S15. Packing diagram for **4**, viewed roughly along the *b*-axis. Disordered atoms and H atoms omitted.

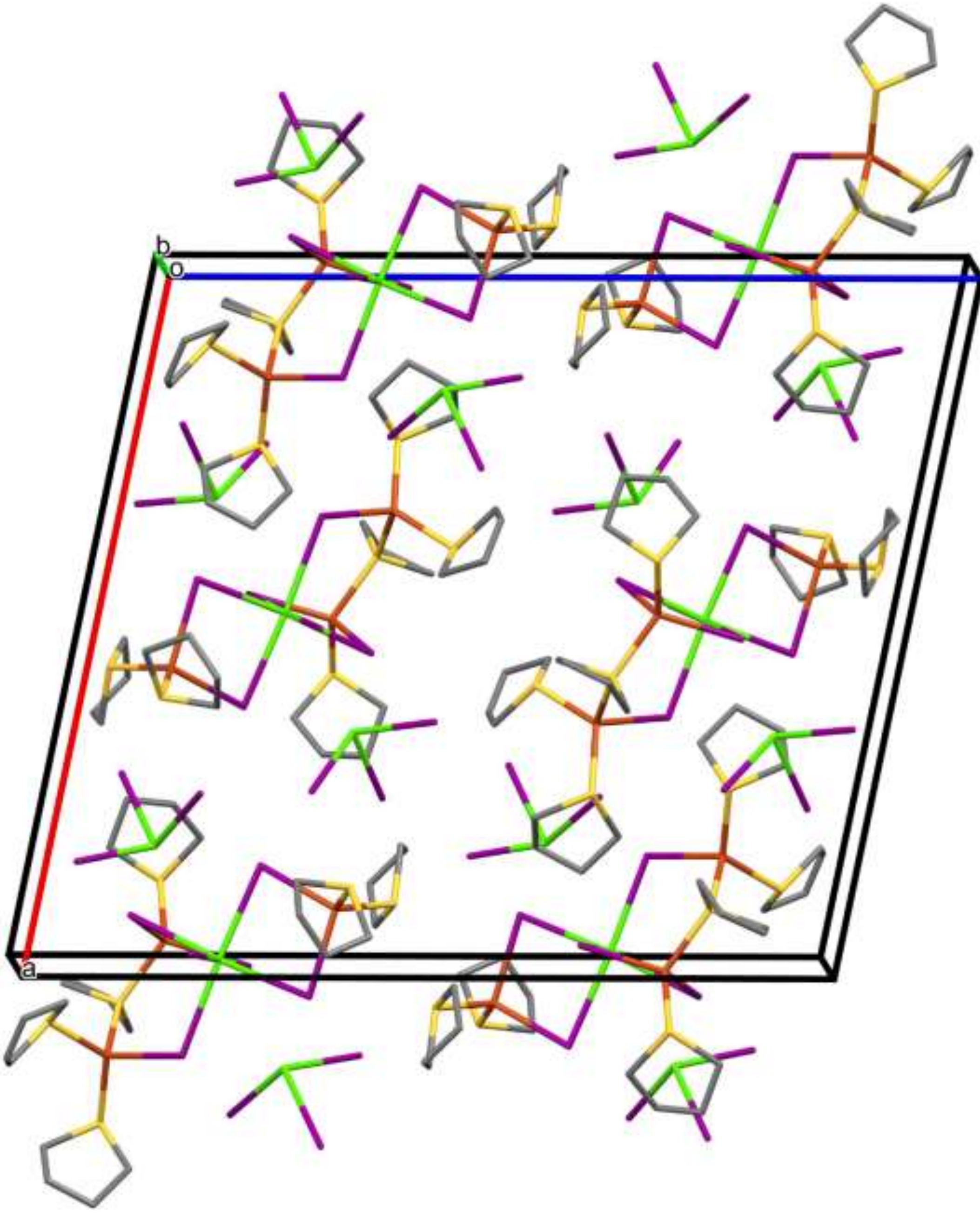

Figure S16. ORTEP diagram of crystallographically independent unit for **5** showing Bi stack and THT disorder. Ellipsoids at 50%.

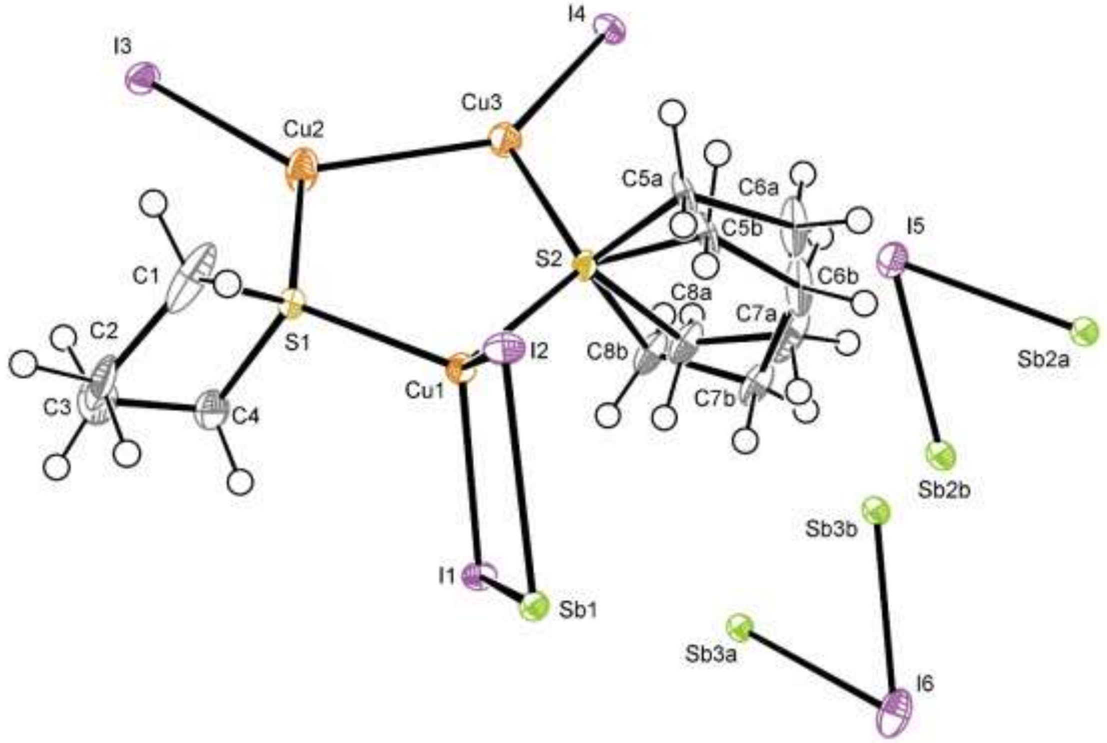

Figure S17. Polyhedral projection of **5**, two views, hydrogen atoms omitted.

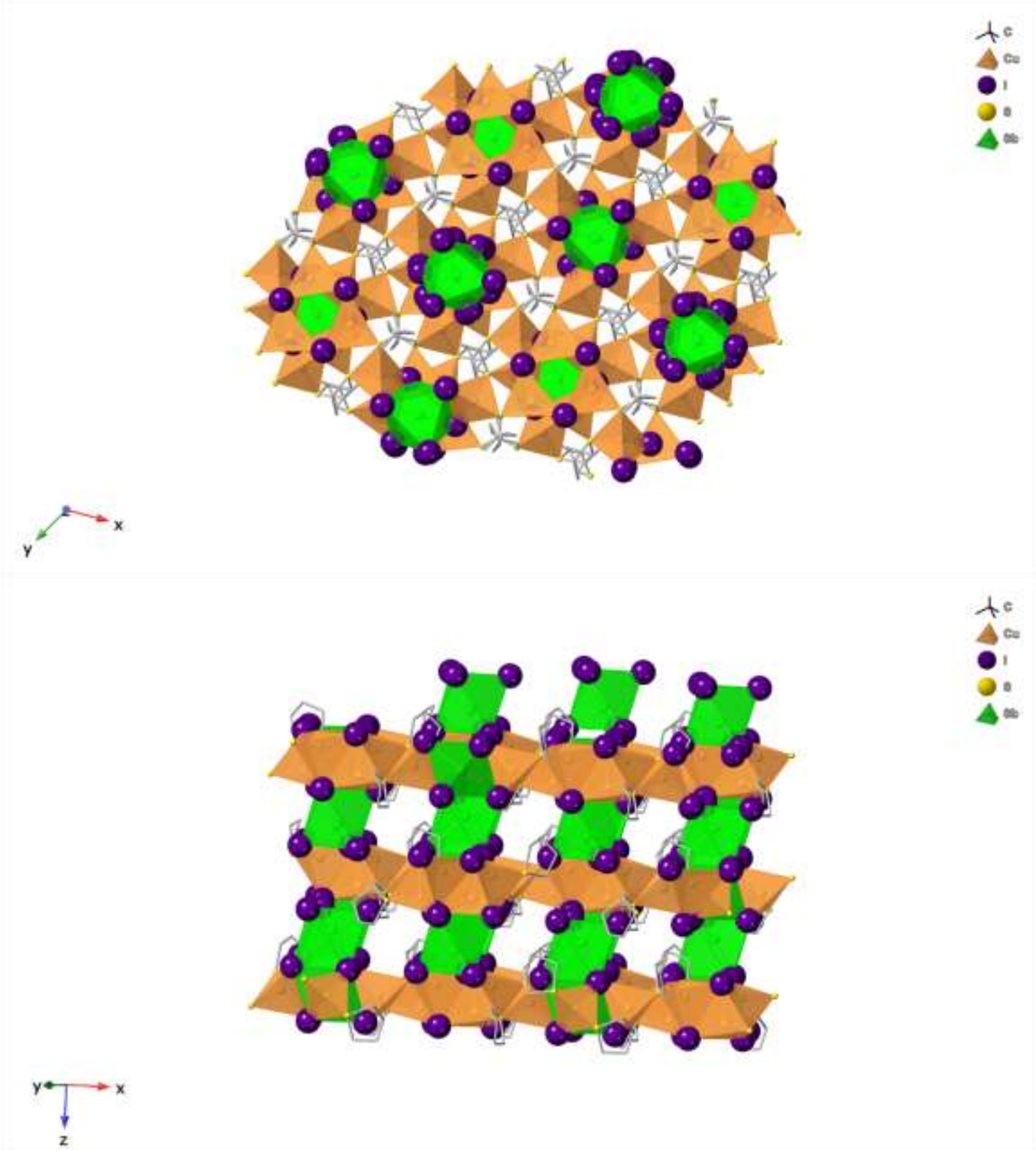

Figure S18. Packing diagram for **5**, viewed roughly along the *c*-axis. Disordered atoms and H atoms omitted.

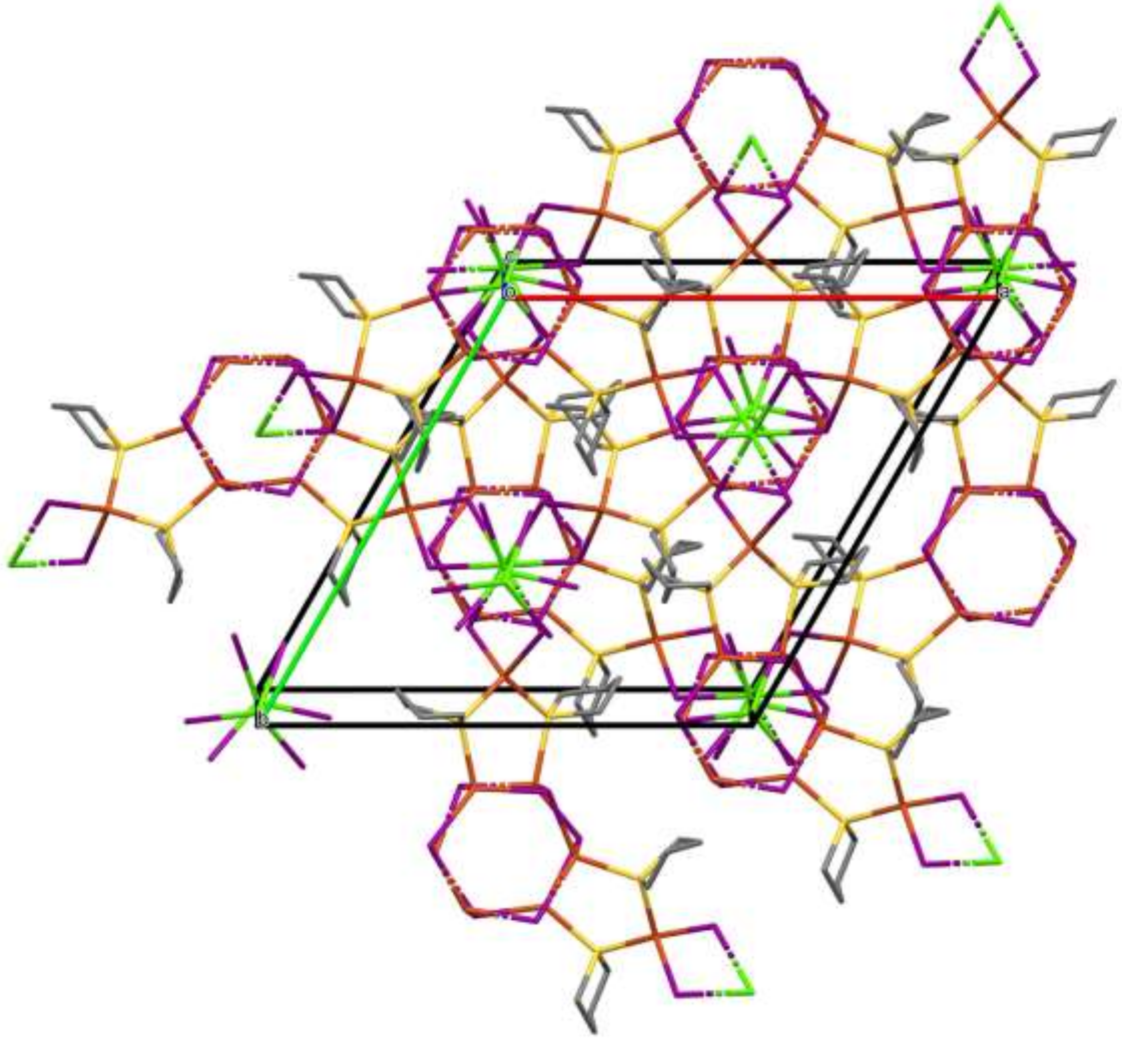

Figure S19. IR Spectrum of **1**.

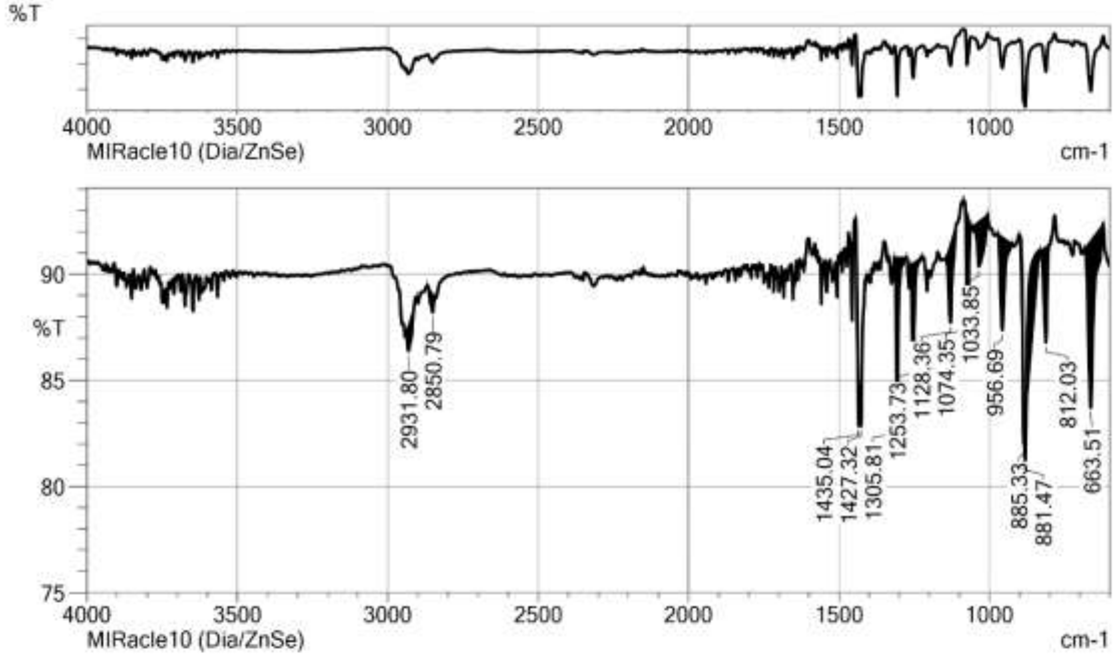

	Peak	Intensity	Corr. Intensity	Base (H)	Base (L)	Area	Corr. Area	Comment
1	663.51	83.70	7.89	684.73	621.08	696.005	173.352	
2	812.03	86.79	4.51	831.32	800.46	321.453	51.915	
3	881.47	81.23	1.35	883.40	831.32	569.667	-123.689	
4	885.33	82.03	1.29	900.76	883.40	203.240	-21.480	
5	956.69	87.37	4.41	972.12	923.90	472.776	72.256	
6	1033.85	90.36	2.15	1058.92	1004.91	459.566	56.152	
7	1074.35	87.85	5.14	1083.99	1058.92	223.275	43.786	
8	1128.36	87.73	3.79	1145.72	1087.85	515.967	57.212	
9	1253.73	85.81	4.72	1261.45	1222.87	408.563	47.760	
10	1305.81	82.97	7.84	1315.45	1274.95	437.560	65.321	
11	1427.32	82.82	2.35	1431.18	1408.04	297.993	-0.918	
12	1435.04	82.87	3.75	1444.68	1431.18	175.472	19.151	
13	2850.79	88.20	0.88	2868.15	2843.07	281.883	10.919	
14	2931.80	86.38	1.20	2937.59	2904.80	409.525	19.664	

Figure S20. IR Spectrum of **2**.

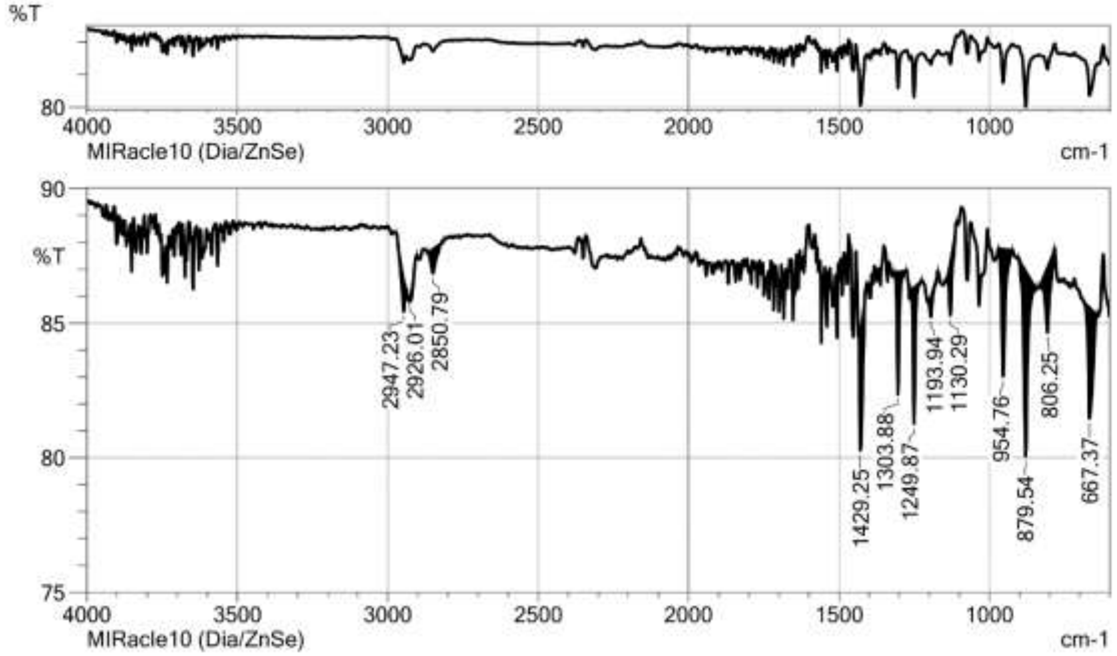

	Peak	Intensity	Corr. Intensity	Base (H)	Base (L)	Area	Corr. Area	Comment
1	667.37	81.47	4.16	688.59	642.30	737.124	70.953	
2	806.25	84.66	2.51	835.18	783.10	720.745	48.226	
3	879.54	80.06	6.95	902.69	842.89	891.189	107.268	
4	954.76	83.02	4.76	968.27	923.90	608.072	65.650	
5	1130.29	85.29	2.12	1141.86	1109.07	428.953	26.329	
6	1193.94	85.24	0.85	1201.65	1170.79	430.474	9.977	
7	1249.87	81.27	5.10	1259.52	1224.80	510.497	39.766	
8	1303.88	82.36	4.57	1315.45	1276.88	543.145	39.584	
9	1429.25	80.28	4.60	1433.11	1408.04	412.533	49.710	
10	2850.79	86.86	0.92	2866.22	2814.14	650.756	18.852	
11	2926.01	85.80	0.30	2929.87	2902.87	363.372	4.100	
12	2947.23	85.43	1.35	2972.31	2939.52	431.771	16.400	

Figure S21. IR Spectrum of **3**.

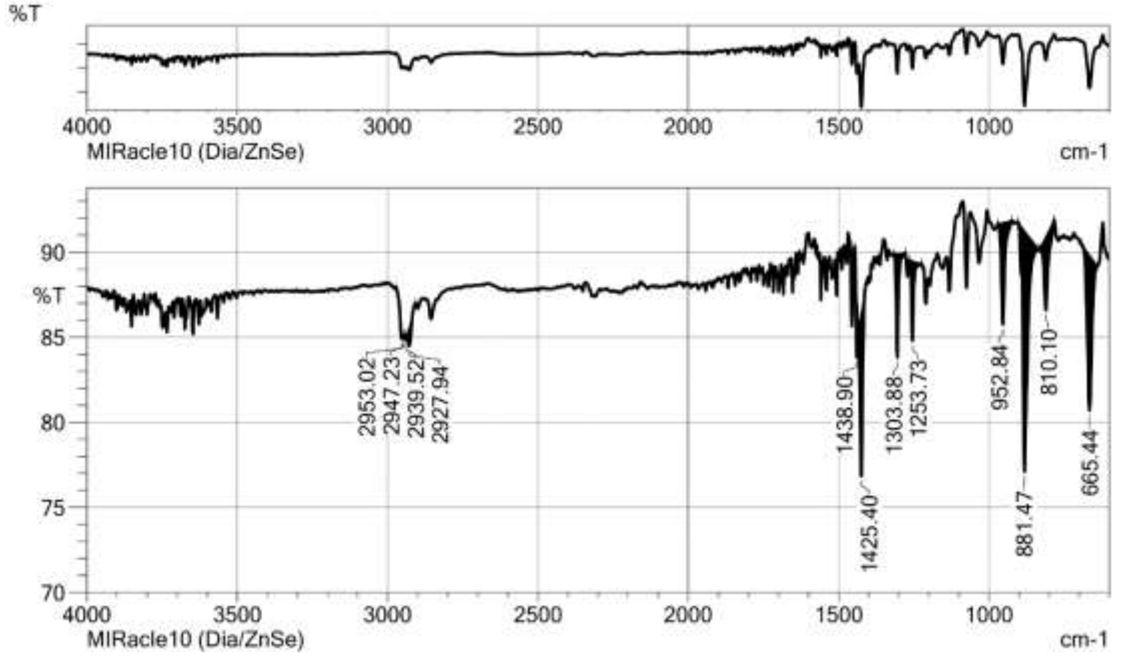

	Peak	Intensity	Corr. Intensity	Base (H)	Base (L)	Area	Corr. Area	Comment
1	665.44	80.70	9.16	692.44	638.44	703.296	155.393	
2	810.10	86.61	4.35	831.32	783.10	502.769	71.966	
3	881.47	77.10	14.21	902.69	835.18	861.744	254.991	
4	952.84	85.74	6.01	970.19	923.90	458.875	78.268	
5	1253.73	84.81	4.69	1263.37	1224.80	446.706	39.575	
6	1303.88	83.83	6.09	1315.45	1278.81	420.393	51.367	
7	1425.40	76.85	9.60	1433.11	1408.04	416.369	89.048	
8	1438.90	83.82	3.85	1446.61	1433.11	184.317	22.396	
9	2927.94	84.49	1.12	2935.66	2906.73	413.737	10.286	
10	2939.52	84.83	0.31	2943.37	2935.66	115.999	1.289	
11	2947.23	84.98	0.14	2951.09	2943.37	115.166	0.401	
12	2953.02	84.91	0.44	2972.31	2951.09	283.669	-2.510	

Figure S22. IR Spectrum of **4**.

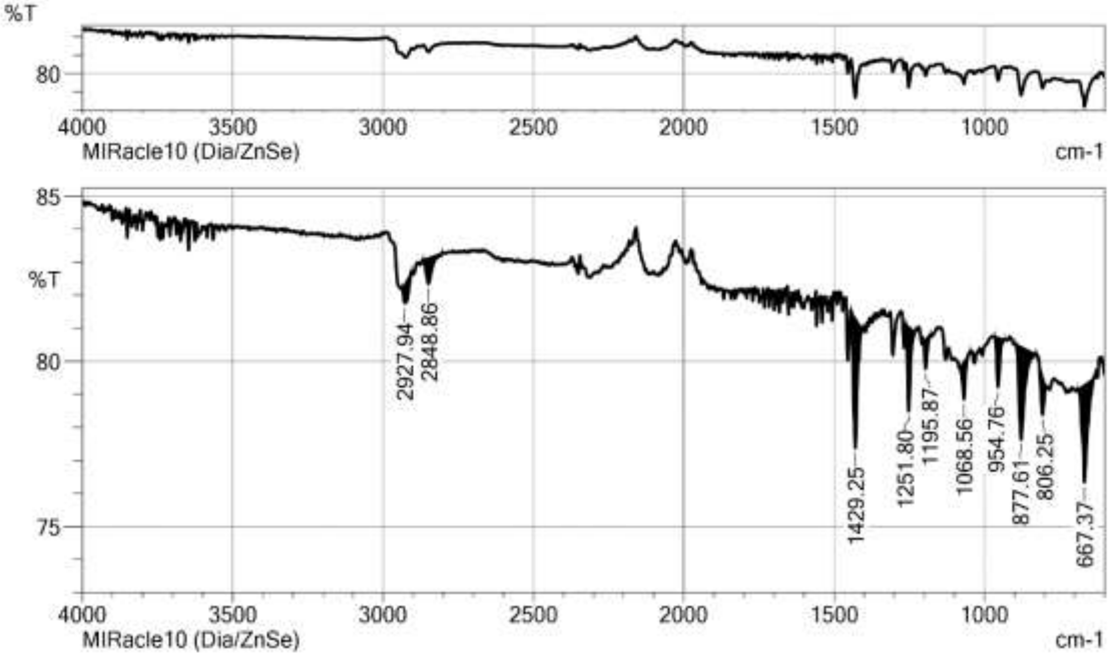

	Peak	Intensity	Corr. Intensity	Base (H)	Base (L)	Area	Corr. Area	Comment
1	667.37	76.35	2.95	688.59	636.51	1132.619	56.121	
2	806.25	78.39	1.20	825.53	794.67	639.688	13.582	
3	877.61	77.63	2.83	896.90	831.32	1346.215	60.449	
4	954.76	79.23	1.51	966.34	933.55	649.288	17.200	
5	1068.56	78.86	1.14	1082.07	1045.42	742.604	12.248	
6	1195.87	79.78	0.92	1203.58	1178.51	492.162	9.124	
7	1251.80	78.50	2.58	1261.45	1230.58	613.588	28.067	
8	1429.25	77.38	3.95	1444.68	1406.11	781.232	58.618	
9	2848.86	82.35	0.79	2866.22	2804.50	1055.139	17.624	
10	2927.94	81.77	0.60	2937.59	2904.80	586.842	12.299	

Figure S23. IR Spectrum of **5**.

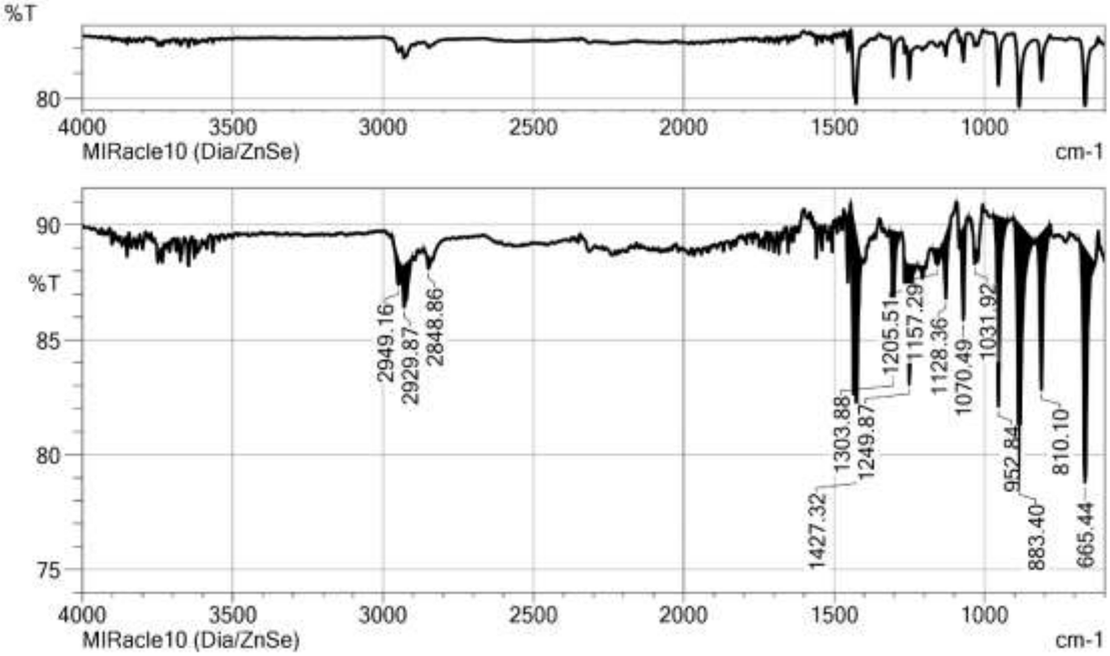

	Peak	Intensity	Corr. Intensity	Base (H)	Base (L)	Area	Corr. Area	Comment
1	665.44	78.81	10.18	686.66	632.65	753.122	152.485	
2	810.10	82.83	6.82	833.25	783.10	609.245	91.889	
3	883.40	78.68	11.37	902.69	833.25	907.057	200.874	
4	952.84	82.11	8.27	970.19	921.97	562.614	97.011	
5	1031.92	88.31	0.79	1053.13	1026.13	281.739	-0.321	
6	1070.49	85.87	4.03	1080.14	1053.13	302.923	32.946	
7	1128.36	86.80	2.81	1143.79	1093.64	535.497	34.029	
8	1157.29	88.27	0.72	1172.72	1143.79	330.024	11.241	
9	1205.51	87.66	0.55	1219.01	1197.79	254.739	4.705	
10	1249.87	83.06	5.24	1261.45	1224.80	474.905	45.732	
11	1303.88	83.41	6.34	1315.45	1274.95	468.852	52.590	
12	1427.32	79.18	10.55	1444.68	1408.04	554.255	175.359	
13	2848.86	88.10	0.52	2866.22	2841.15	287.495	4.632	
14	2929.87	86.42	1.85	2939.52	2902.87	458.506	32.426	
15	2949.16	87.40	1.16	2972.31	2939.52	380.386	14.838	

Figure S24. Thermogravimetric analysis trace for **1**.

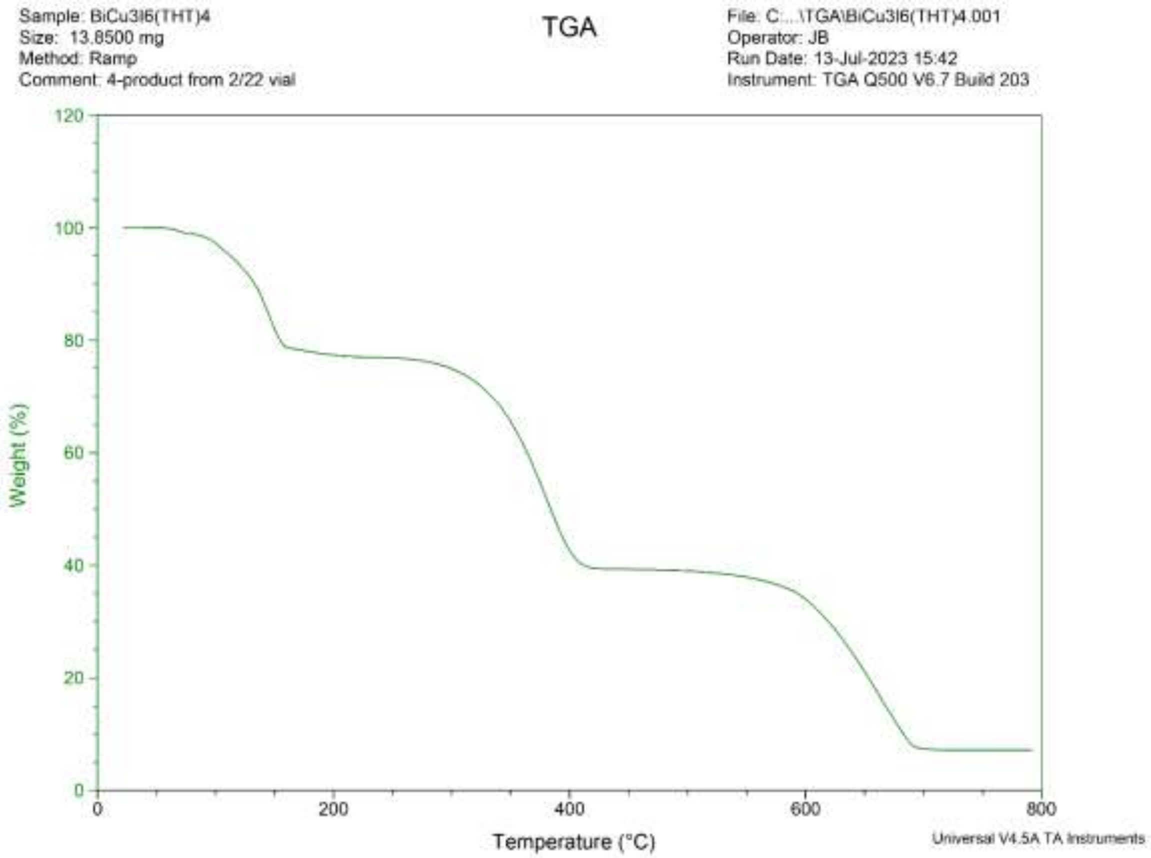

Figure S25. Thermogravimetric analysis trace for **2**.

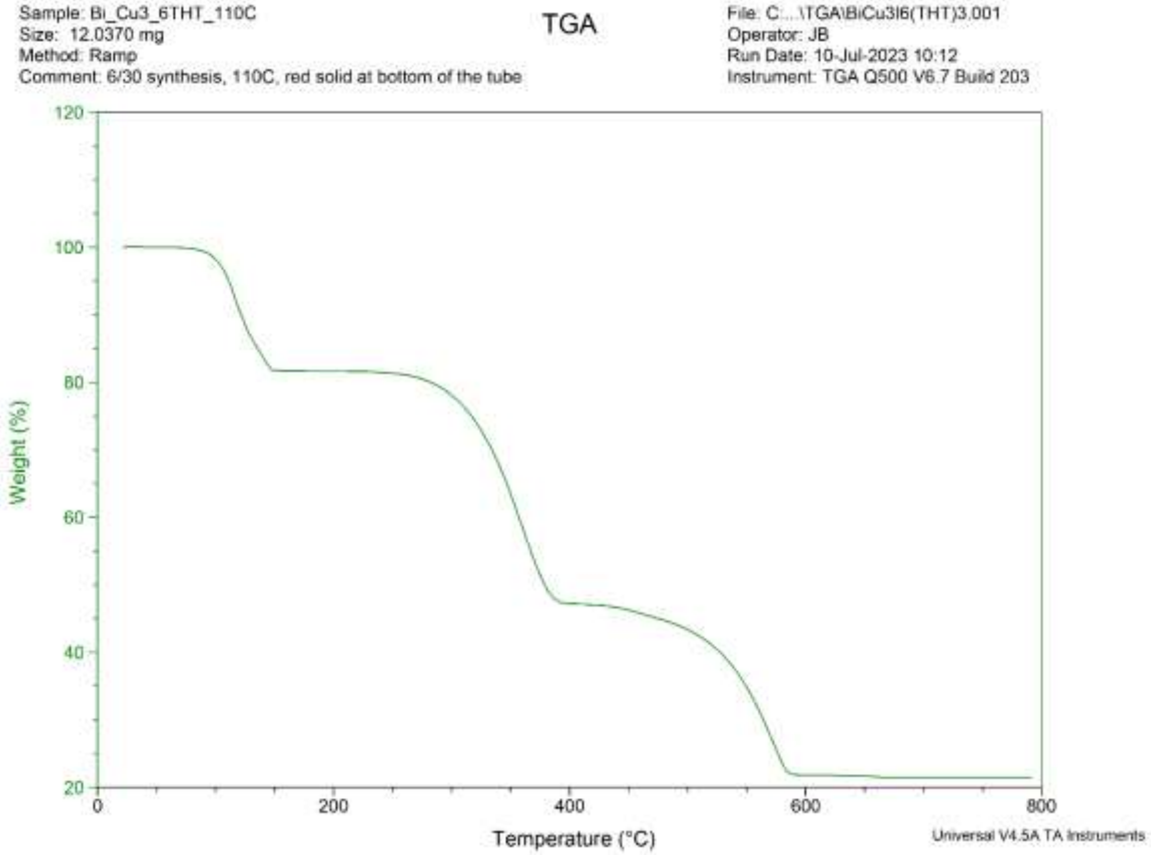

Figure S26. Thermogravimetric analysis trace for **3**.

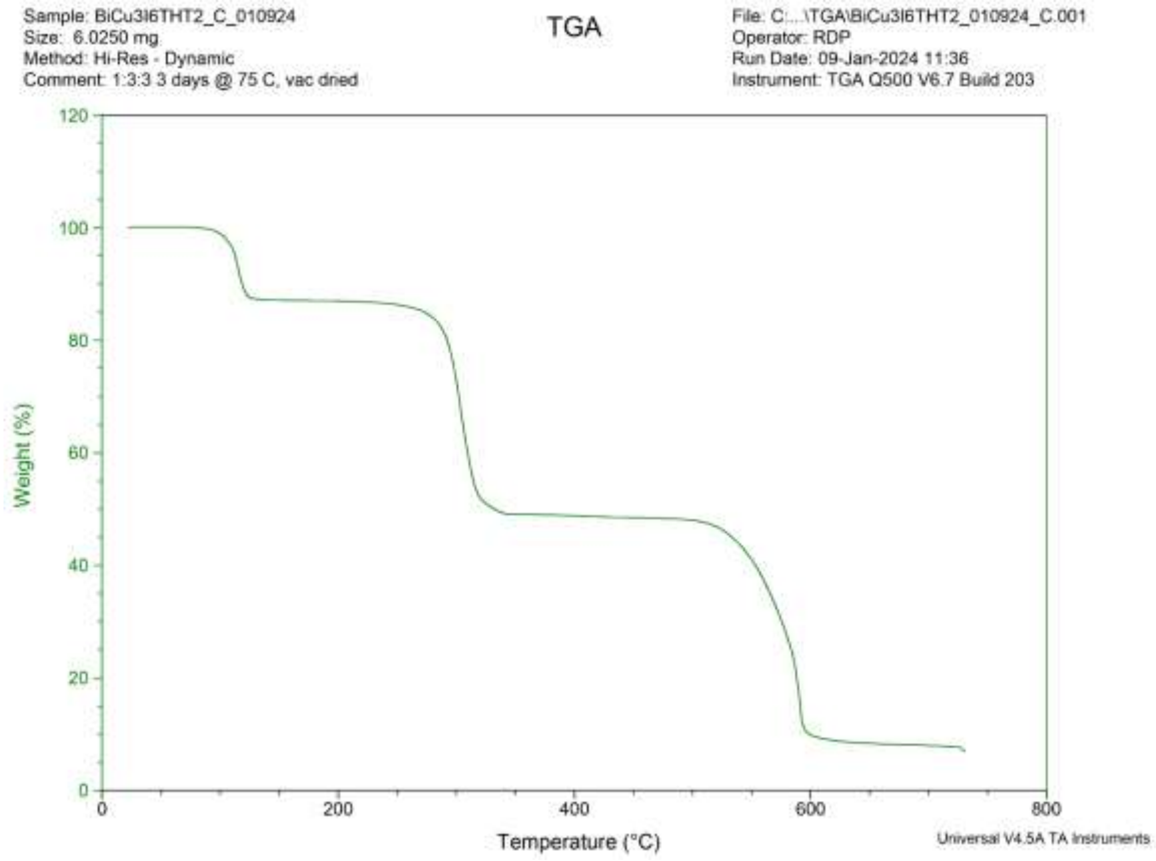

Figure S27. Thermogravimetric analysis trace for **4**.

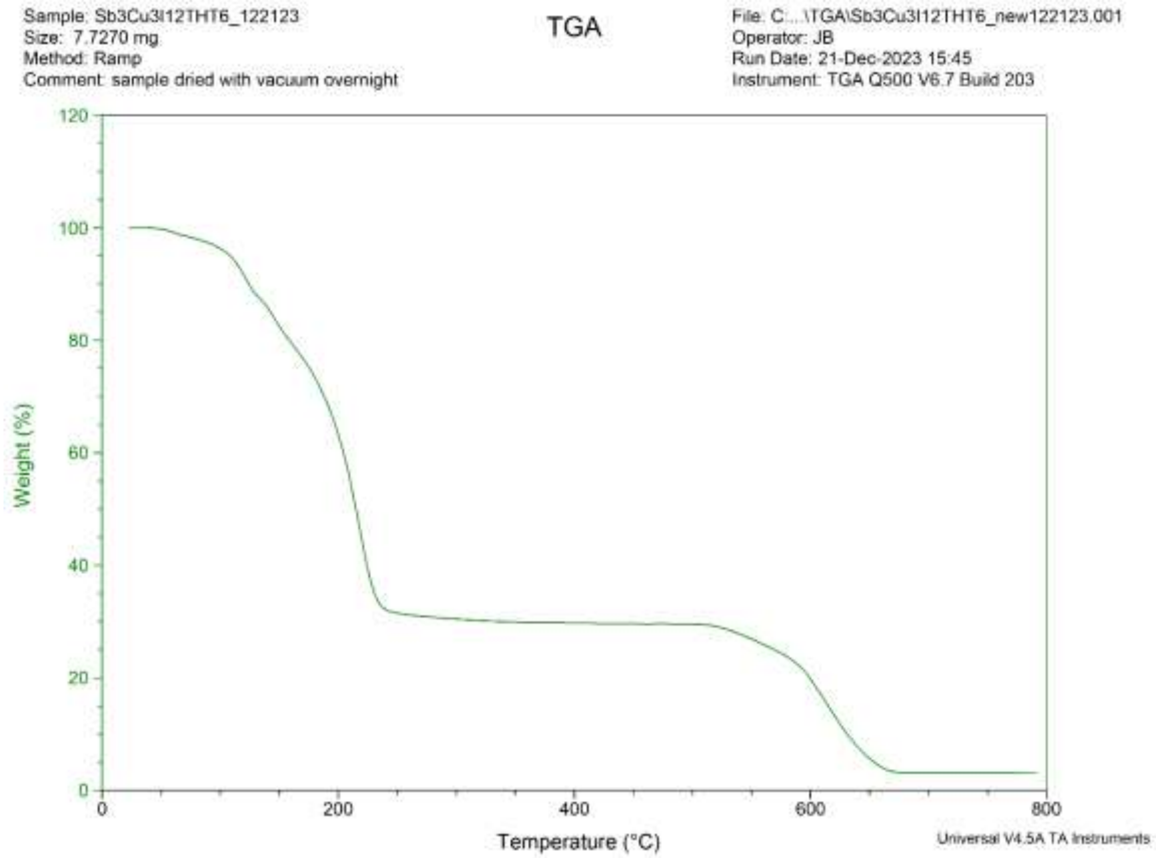

Figure S28. Thermogravimetric analysis trace for **5**.

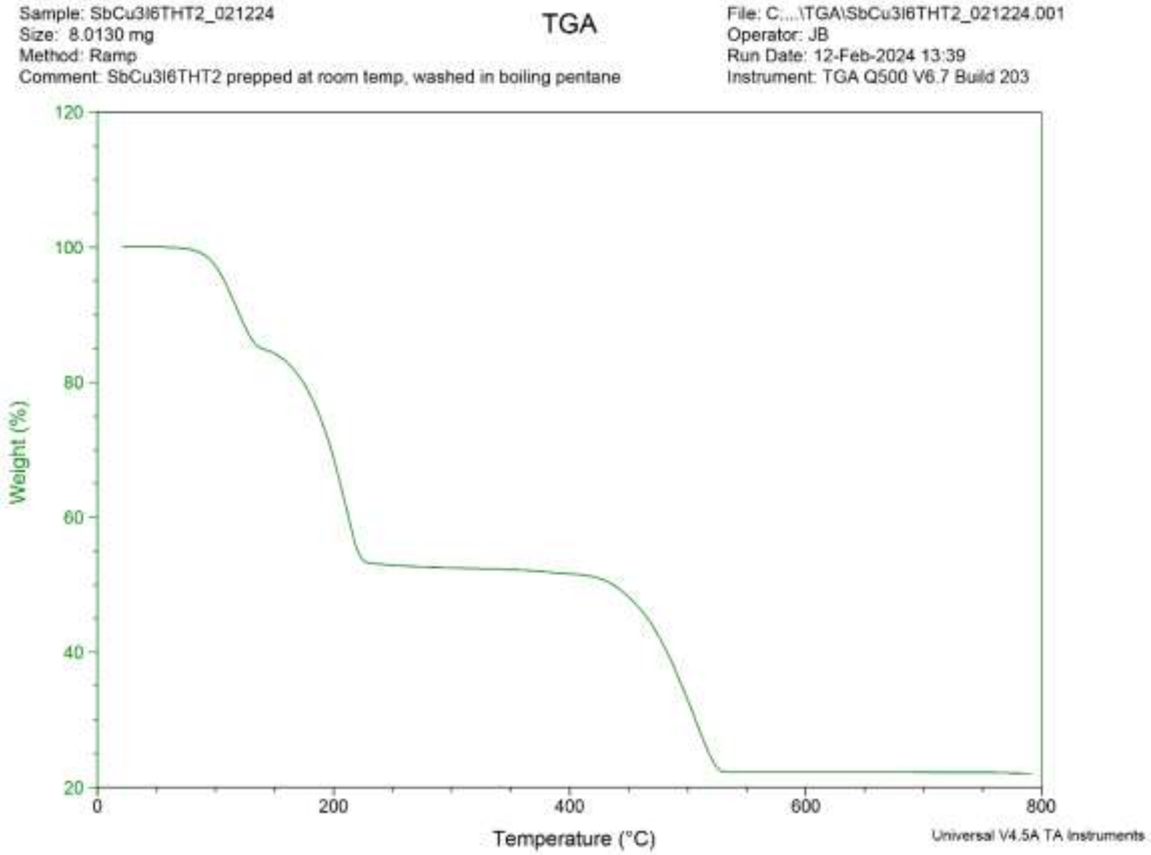

Figure S29. Powder diffraction for **1**.

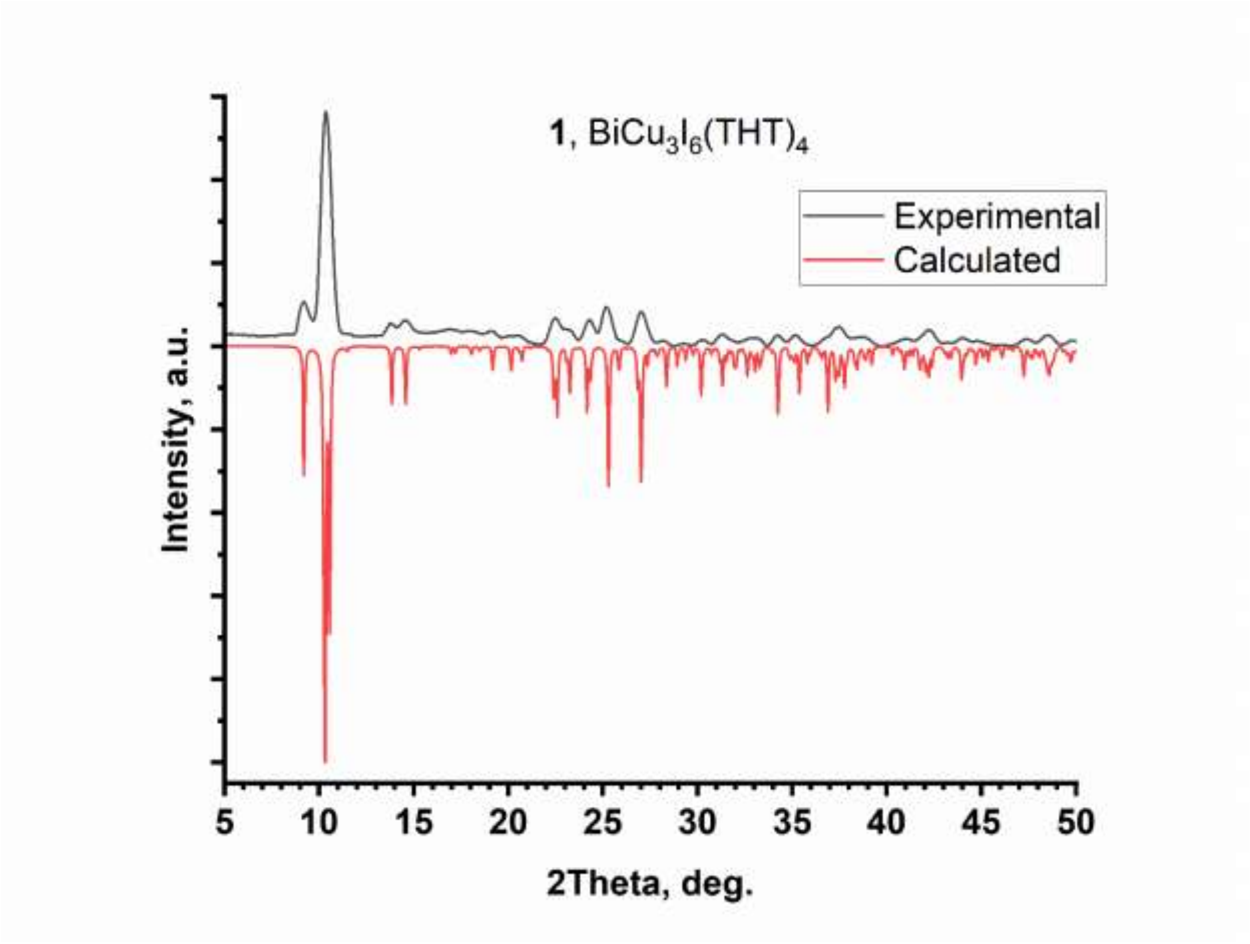

Figure S30. Powder diffraction for **2**.

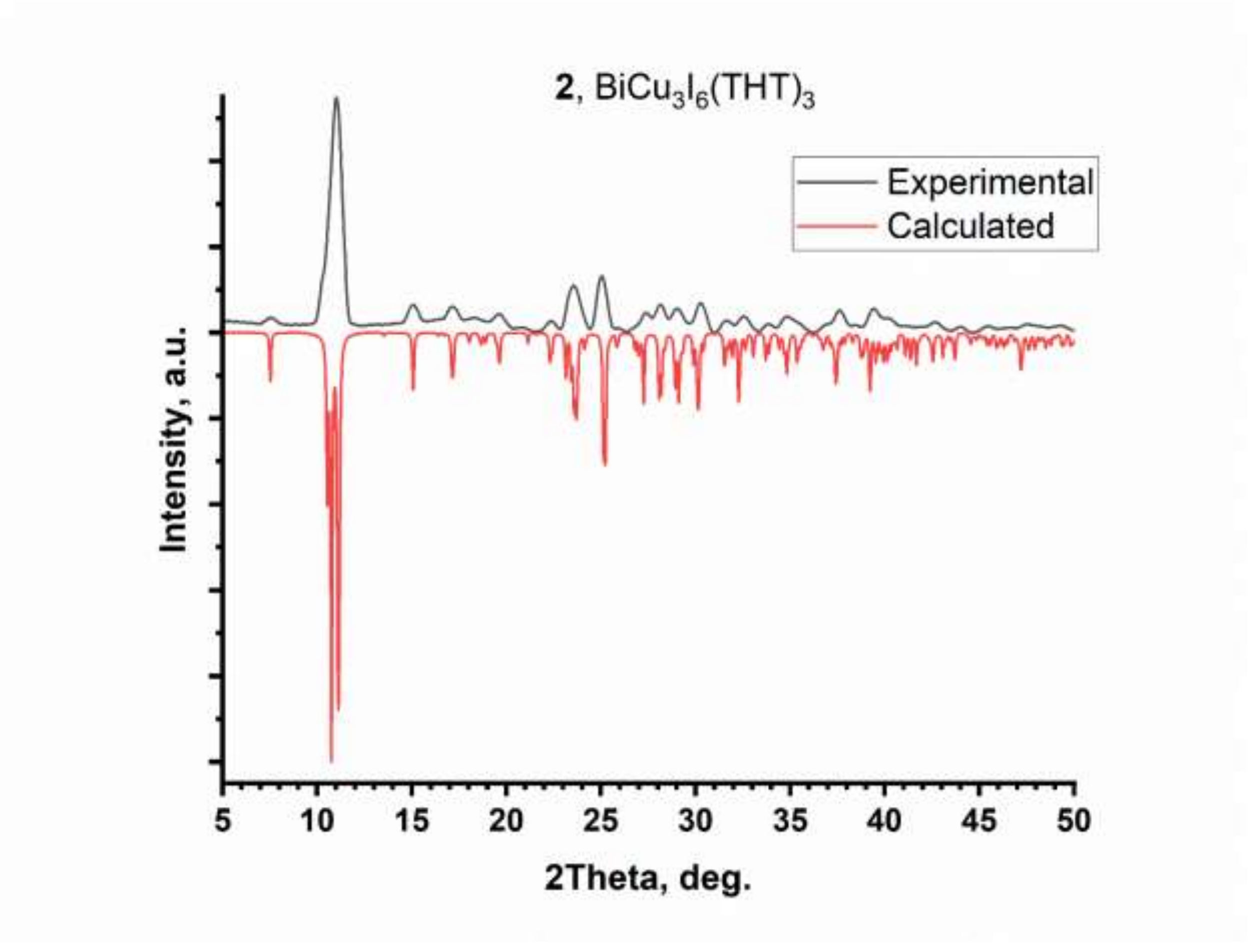

Figure S31. Powder diffraction for **3**. Since this sample is necessarily a mixture of **3A** and **3B**, calculated patterns for both compounds are shown.

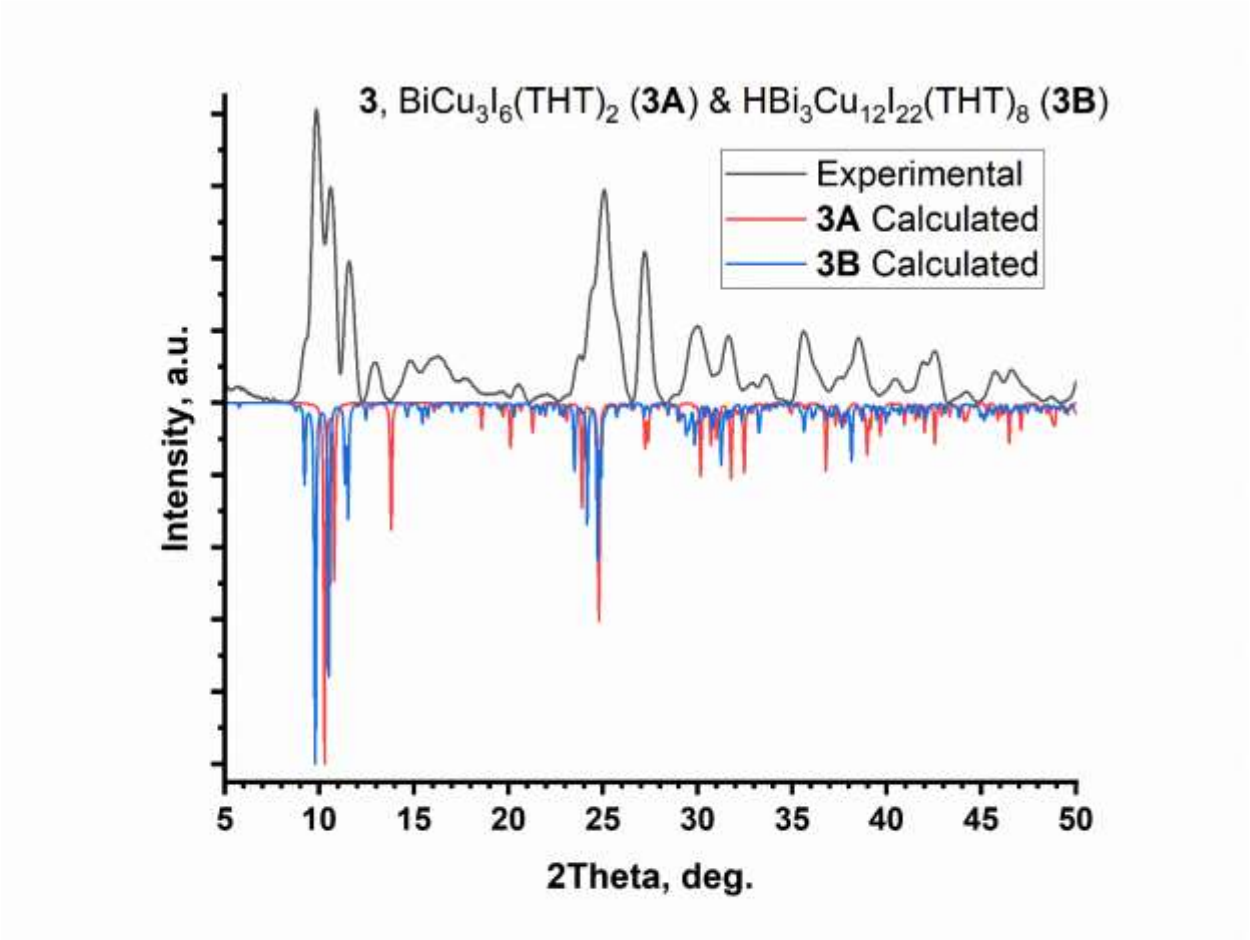

Figure S32. Powder diffraction for **4**.

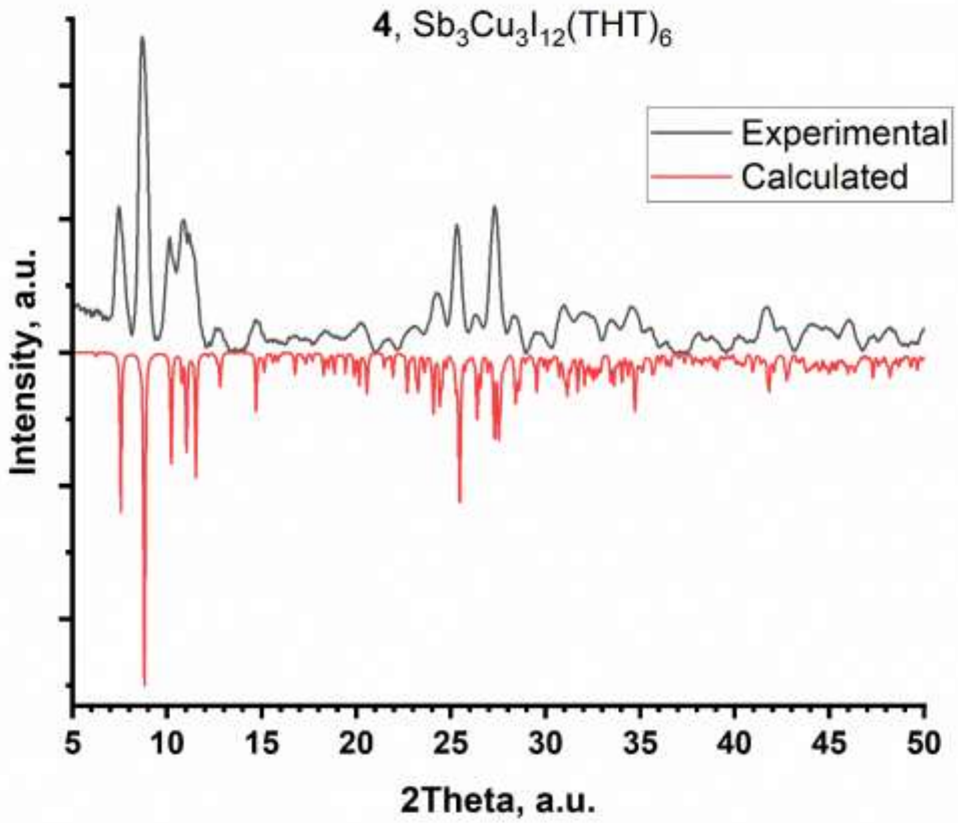

Figure S33. Powder diffraction for **5**.

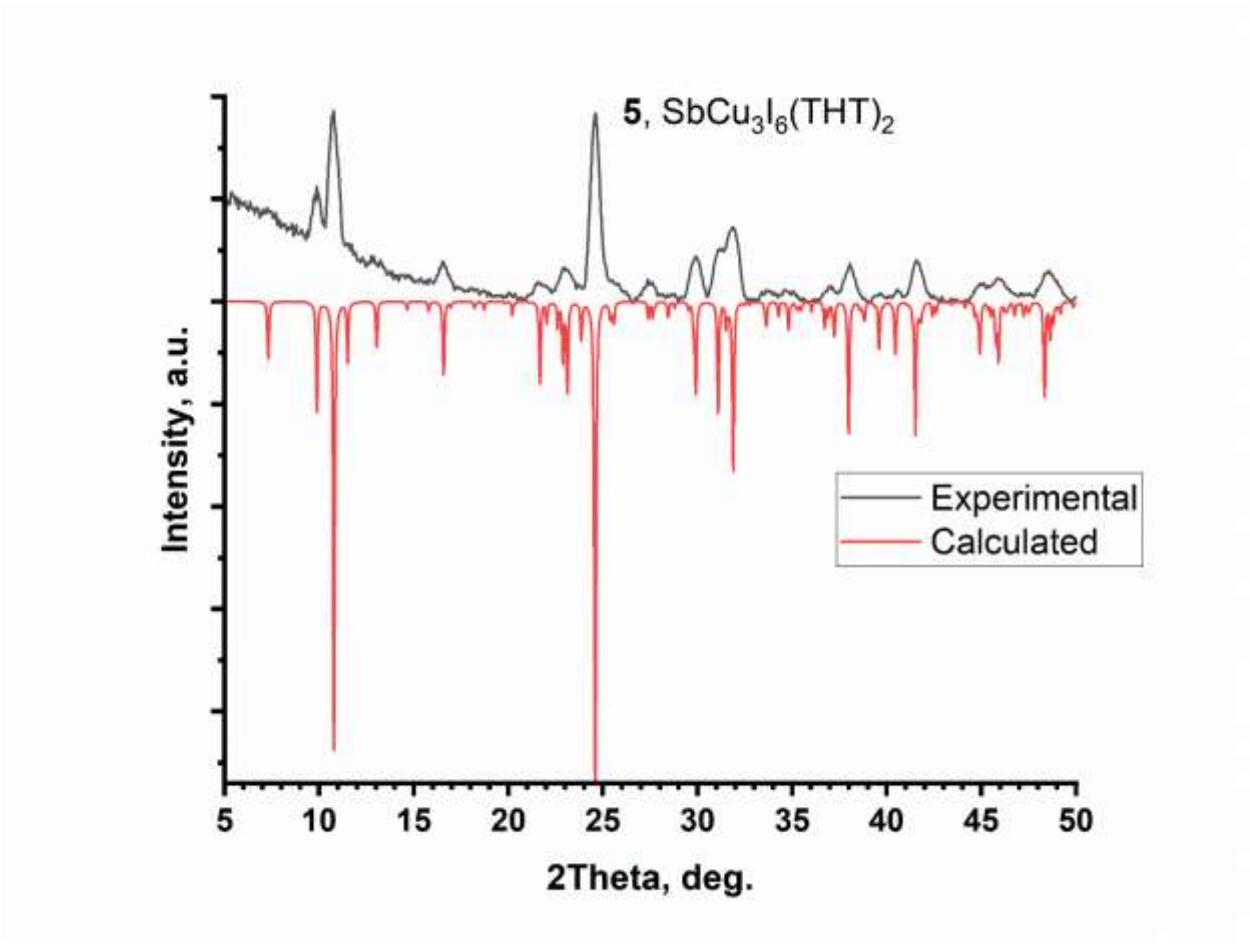

Figure S34. Hirshfeld fingerprint graphs and surfaces for **1**, from top: complete, H−H, and H−I.

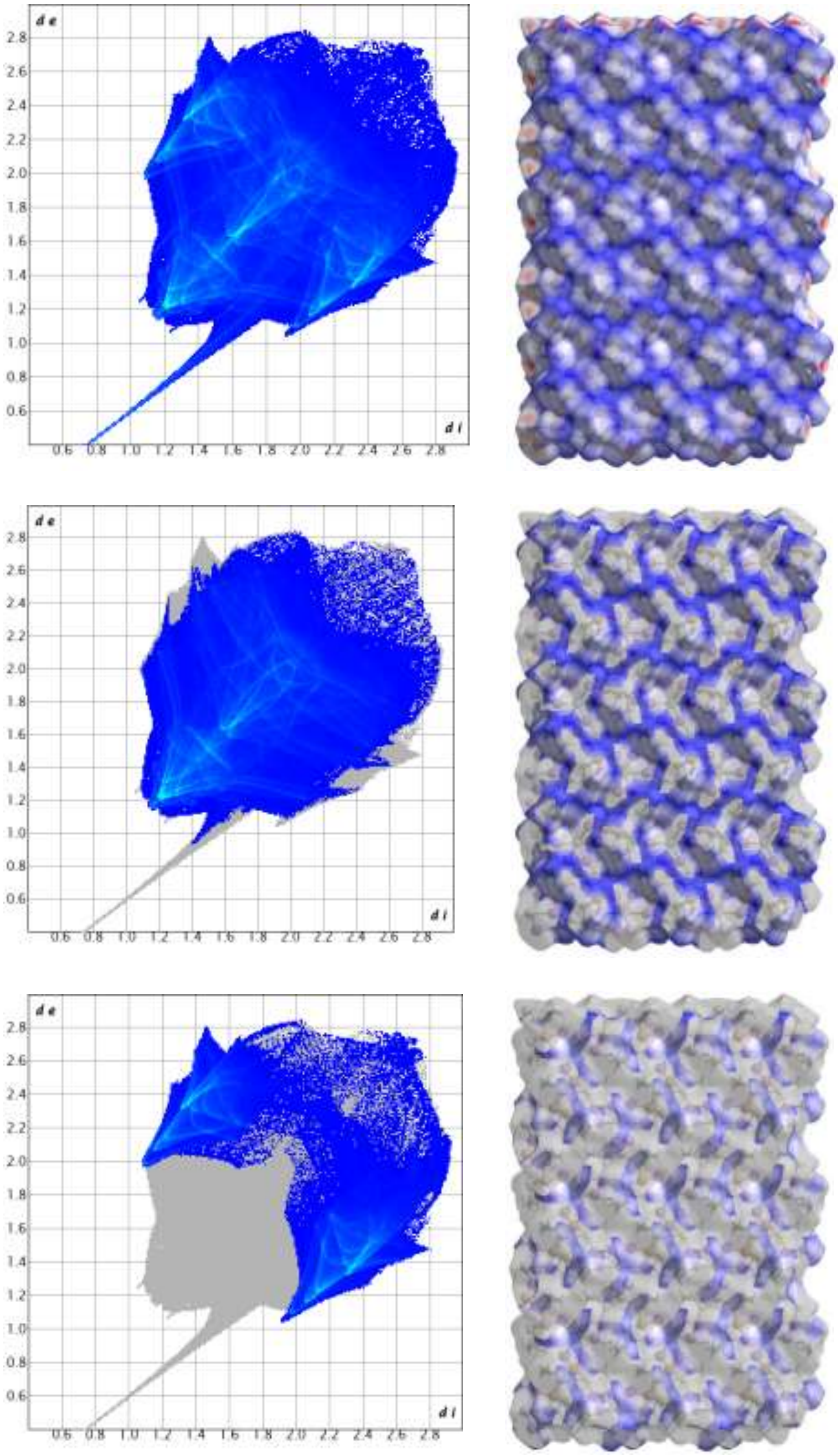

Figure S35. Hirshfeld fingerprint graphs and surfaces for **2**, from top: complete, H−H, and H−I.

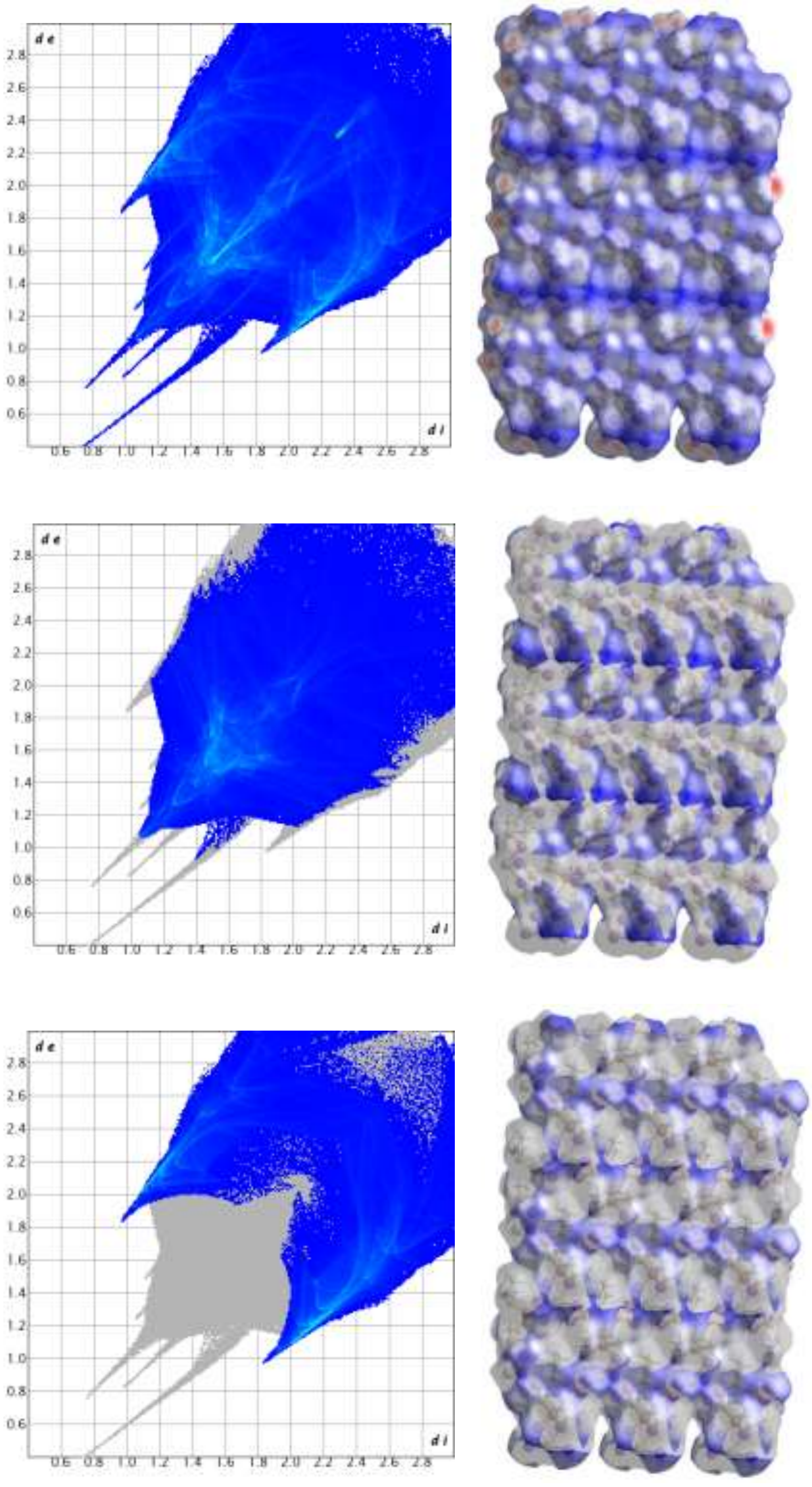

Figure S36. Hirshfeld fingerprint graphs and surfaces for **3A**, from top: complete, H−H, and H−I.

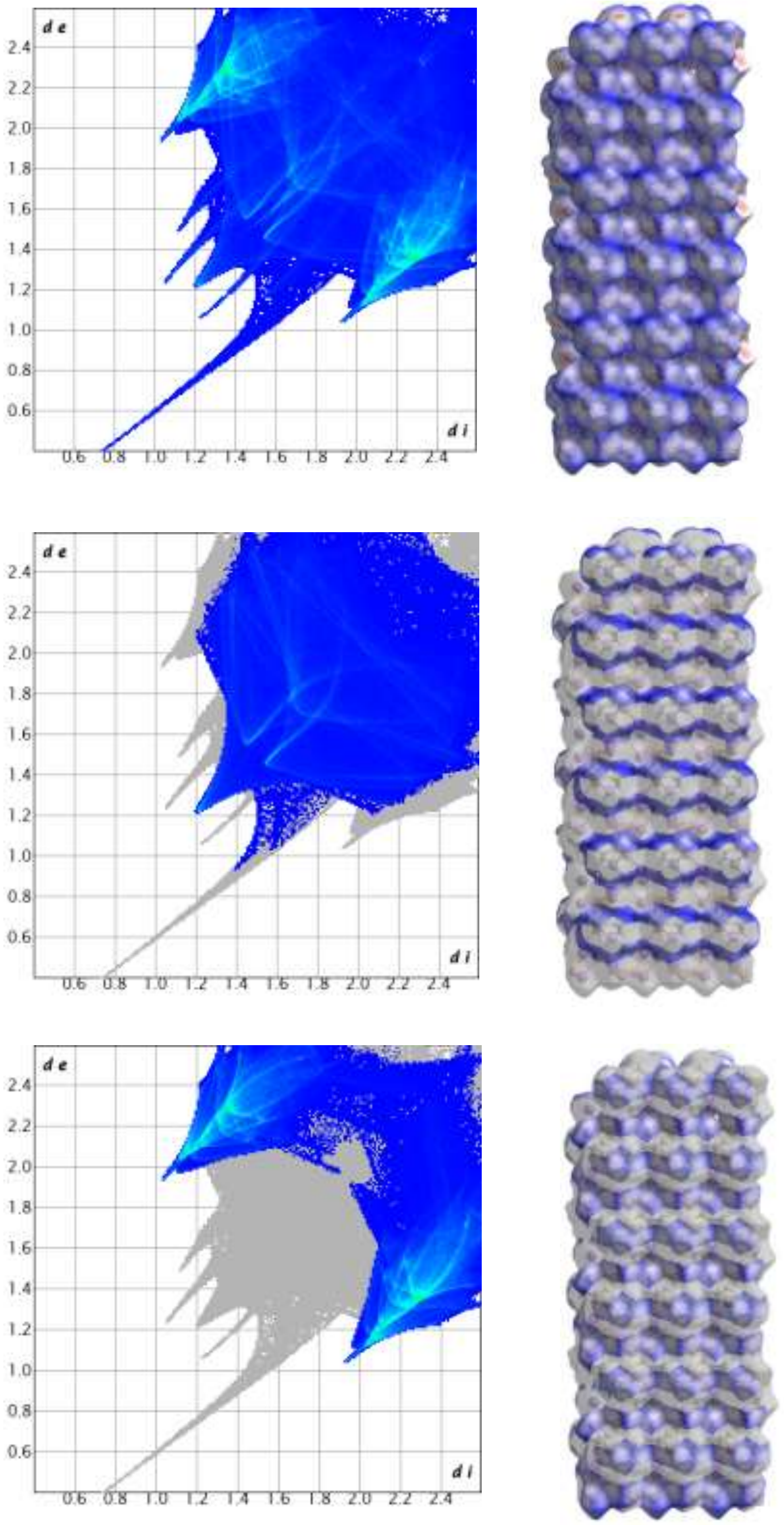

Figure S37. Hirshfeld fingerprint graphs and surfaces for **4**, from top: complete, H−H, and H−I.

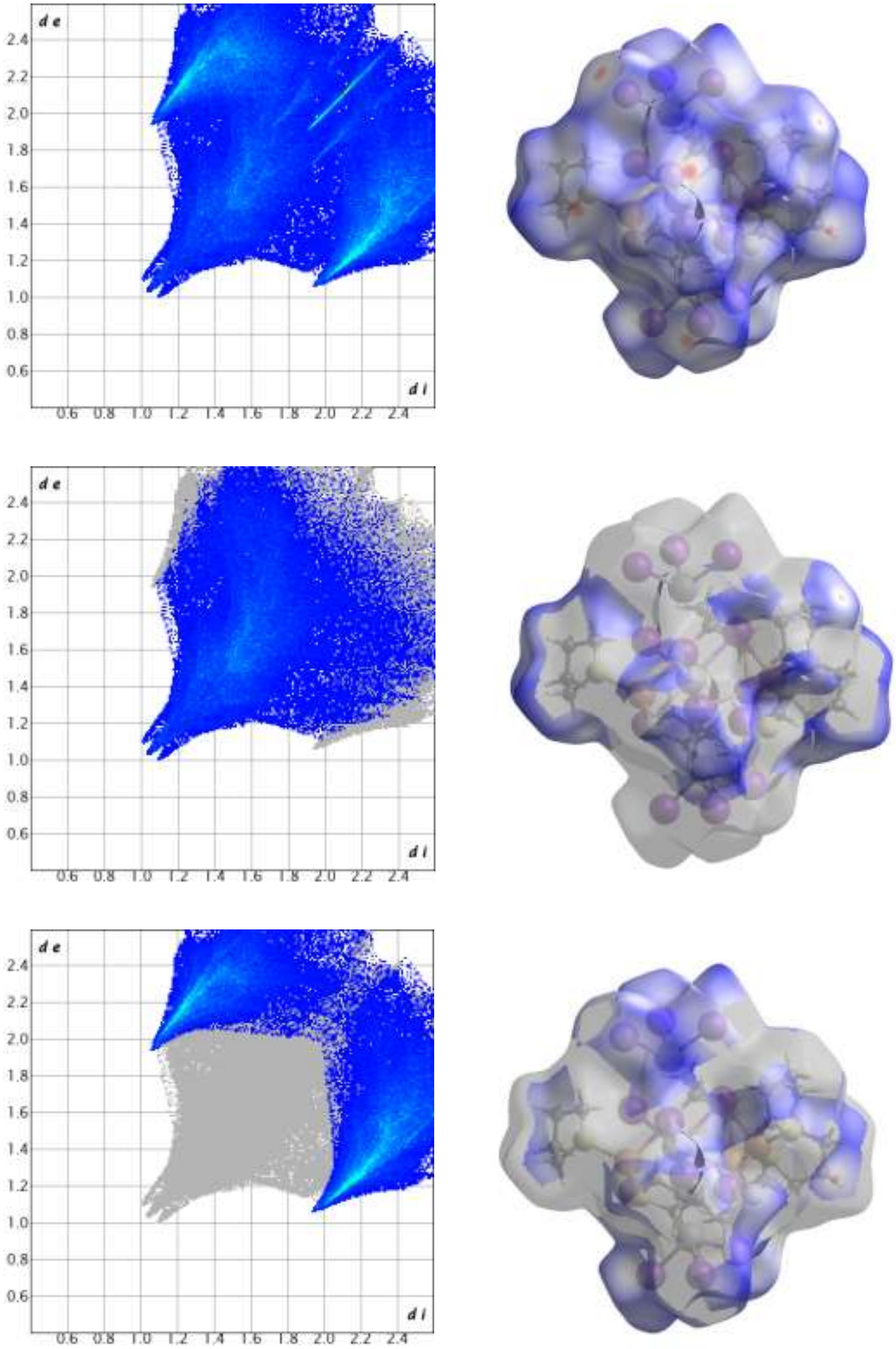

Figure S38. Hirshfeld fingerprint graphs and surfaces for **5**, from top: complete, H−H, and H−I.

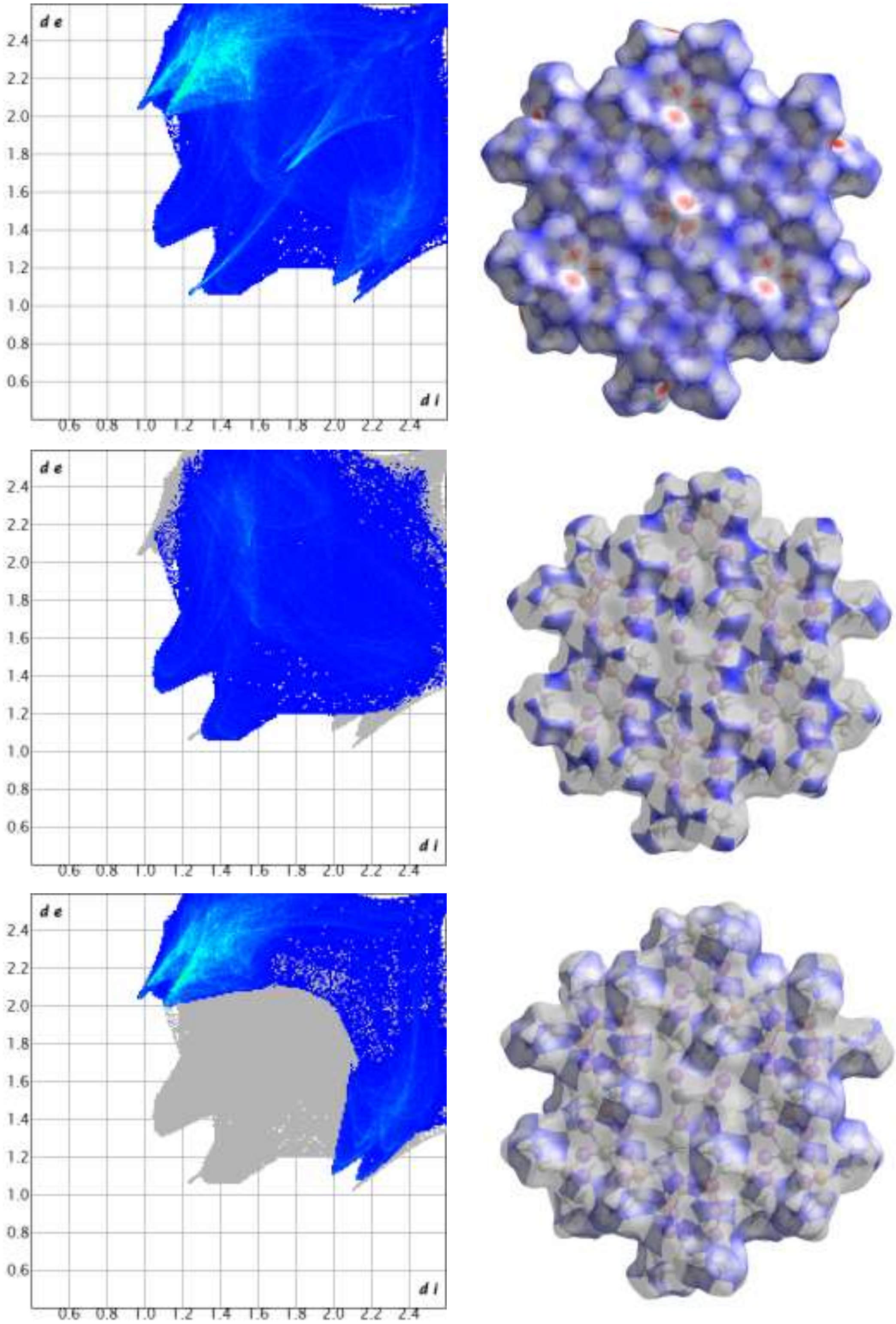

Figure S39. Truncated model of **1** used in DFT and QTAIM calculations. Hydrogens omitted for clarity only.

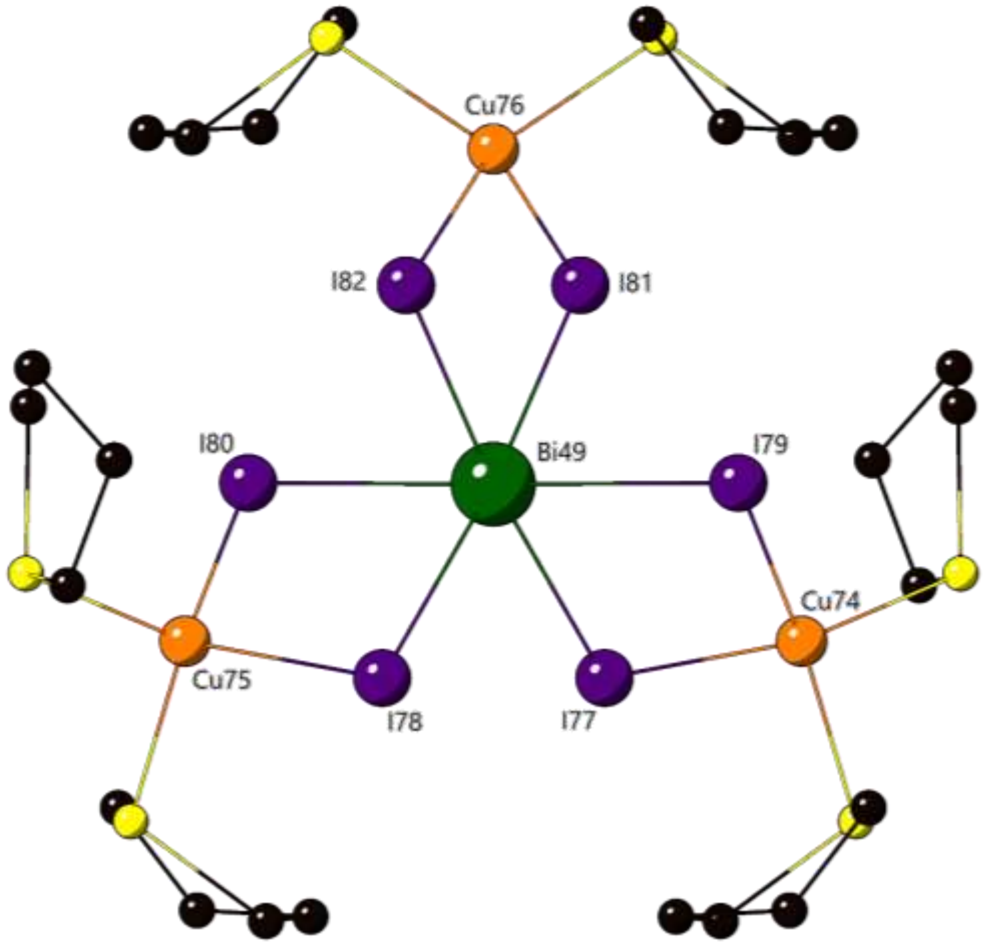

Figure S40. Truncated model of **2** used in DFT and QTAIM calculations. Hydrogens omitted for clarity only.

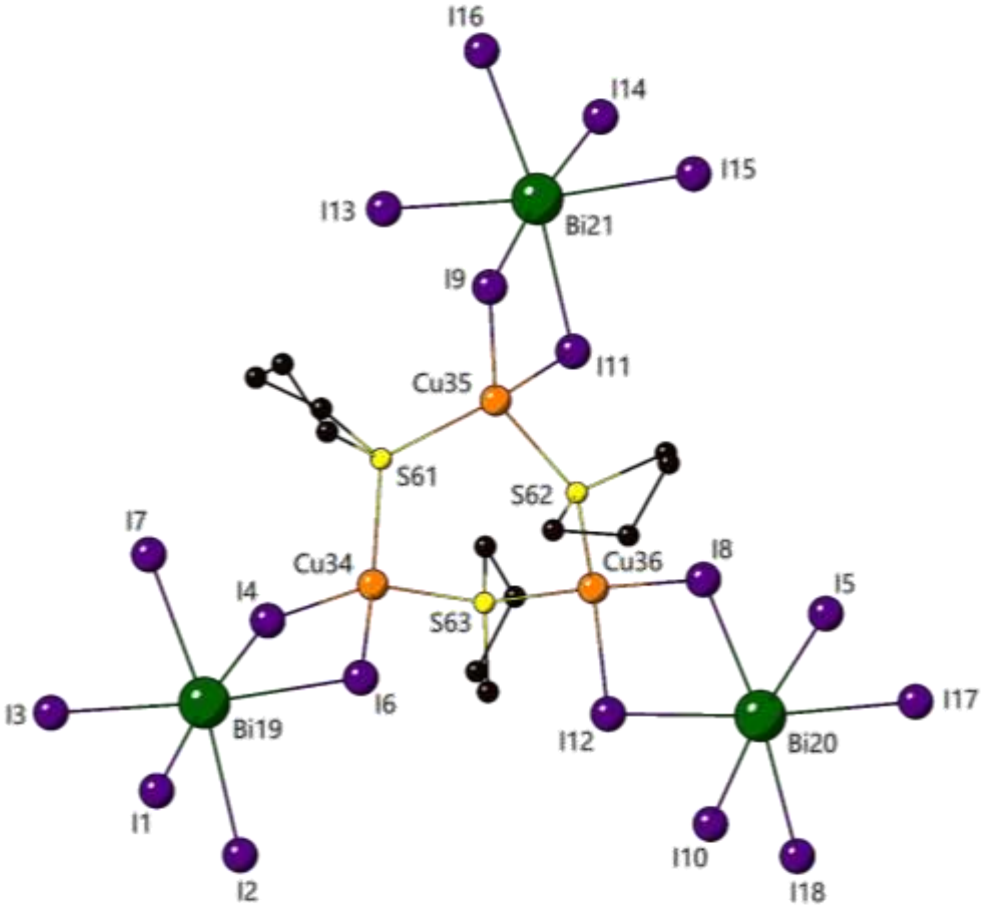

Figure S41. Truncated model of **3A** used in DFT and QTAIM calculations. Hydrogens omitted for clarity only.

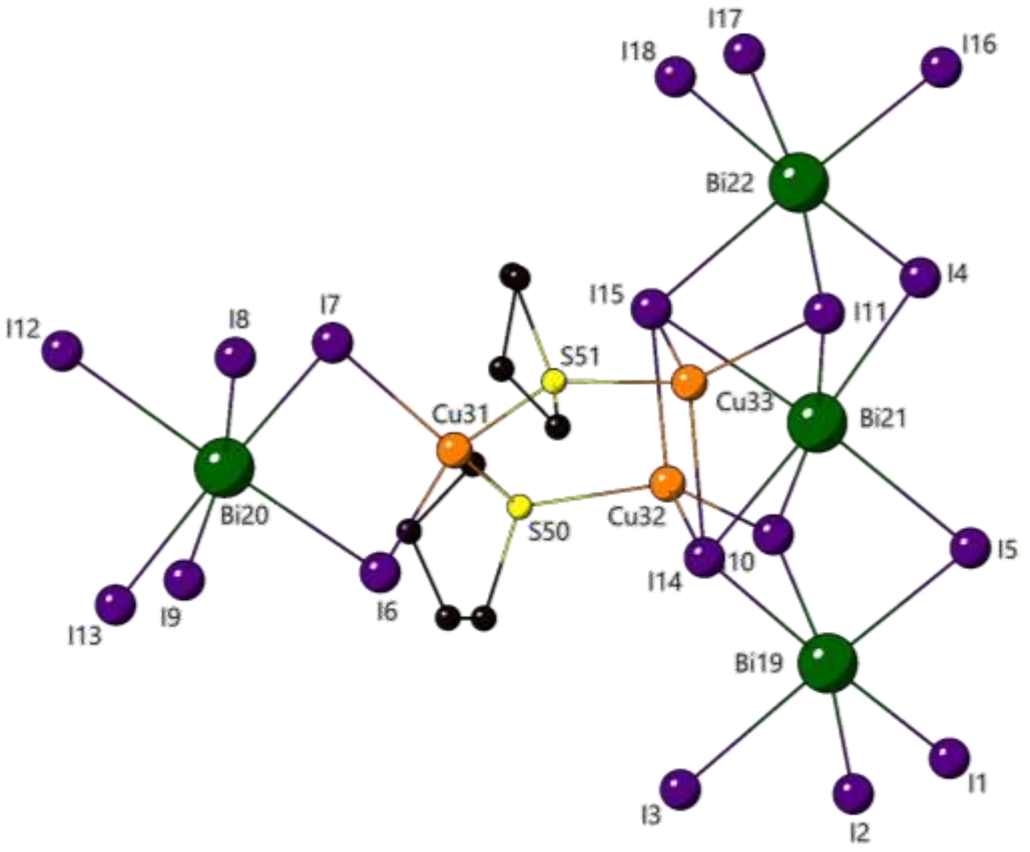

Figure S42. Truncated model of **4** used in DFT and QTAIM calculations. Hydrogens omitted for clarity only.

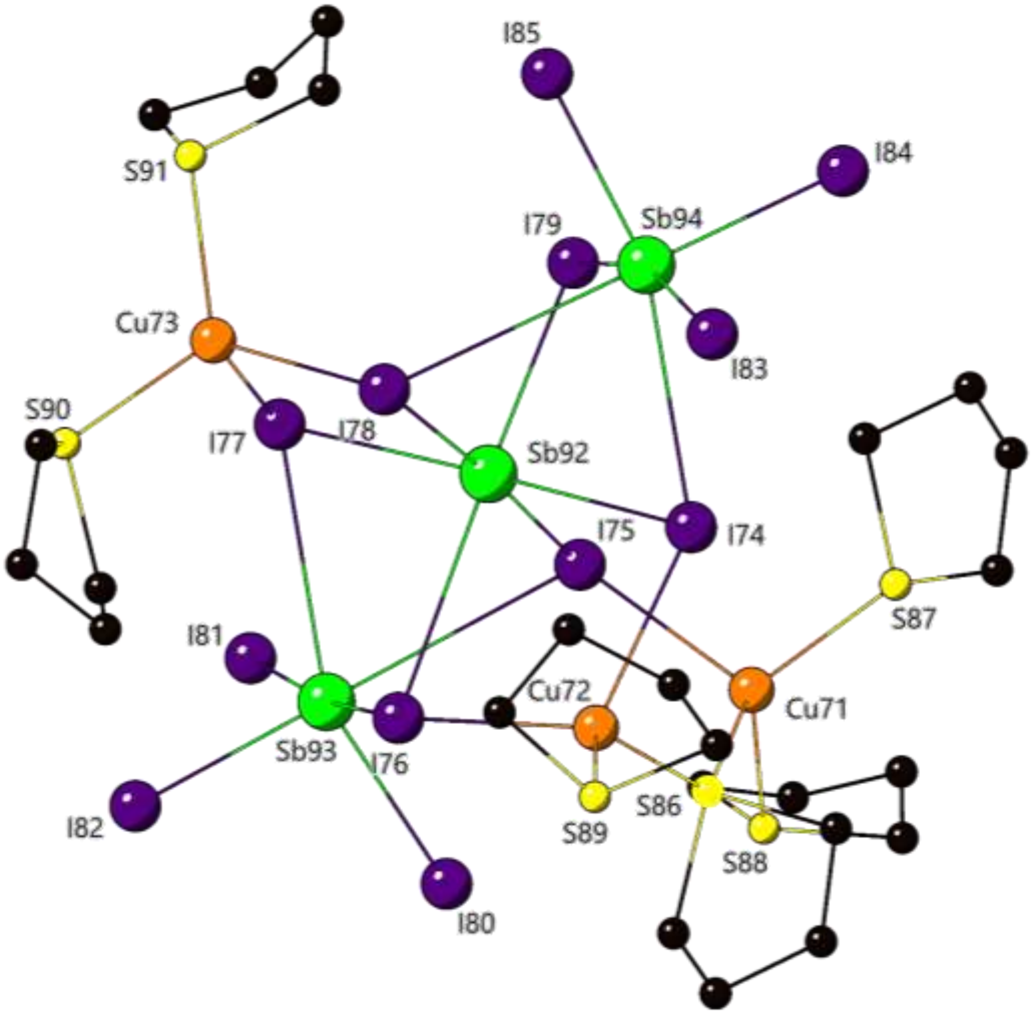

Figure S43. Truncated model of **5** used in DFT and QTAIM calculations. Hydrogens omitted for clarity only.

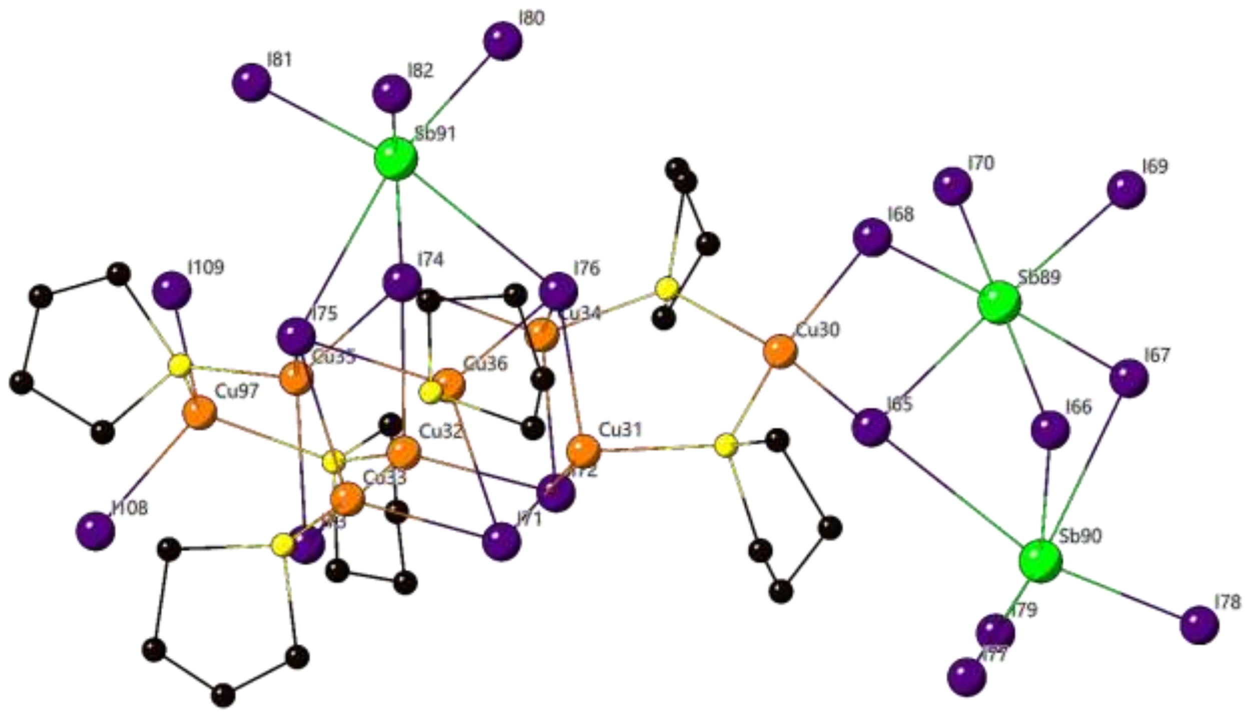

Figure **S44**. Tauc plots of reported compounds.

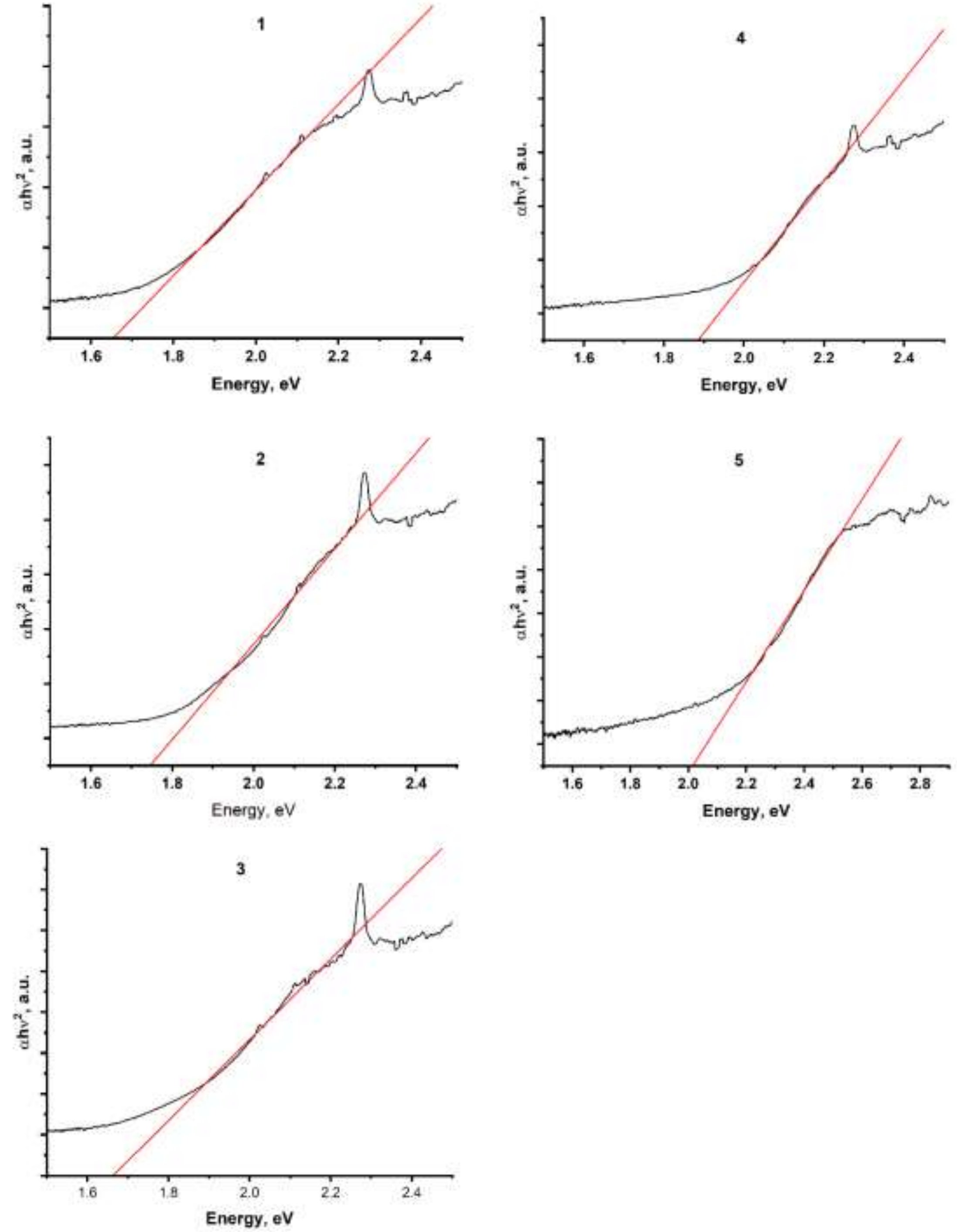

Table S1. Summary of QTAIM parameters of **1-5**.

	**Bond**	**Connection**	**Bond Analyzed**	**ρ**	**$\|Grad(\rho)\|$**	**∇^2**	**G_b**	**V_b**	**H_b**	**$-G_b/V_b$**	**$\|V\|/G$**	**H/ρ**	**G/ρ**
**1**	Bi-I(μ2)	Bi-I-Cu	All Bi-I	3.56E-02	4.00E-16	5.25E-02	1.98E-02	-2.65E-02	-6.66E-03	7.48E-01	1.34E+00	-1.87E-01	5.57E-01
	Cu-I(μ2)		All Cu-I	4.46E-02	8.01E-14	1.27E-01	3.73E-02	-4.28E-02	-5.50E-03	8.72E-01	1.15E+00	-1.23E-01	8.37E-01
**2**	Bi-I(μ2)	Bi-I-Cu	All-Bi-I	3.50E-02	8.06E-15	5.54E-02	2.00E-02	-2.61E-02	-6.14E-03	7.66E-01	1.31E+00	-1.75E-01	5.71E-01
	Cu-I(μ2)		All-Cu-I	4.73E-02	2.17E-13	1.32E-01	3.98E-02	-4.66E-02	-6.74E-03	8.56E-01	1.17E+00	-1.42E-01	8.42E-01
**3A**	Bi-I(μ2)	Bi-I-Cu	Bi20-I6,Bi20-I7	4.02E-02	4.19E-16	6.71E-02	2.48E-02	-3.27E-02	-7.98E-03	7.56E-01	1.32E+00	-1.98E-01	6.15E-01
	Bi-I(μ3)	Bi2-I-Cu	Bi19-I10	3.54E-02	7.64E-14	6.59E-02	2.19E-02	-2.74E-02	-5.47E-03	8.00E-01	1.25E+00	-1.55E-01	6.20E-01
	Bi-I(μ4)	Bi2-I-Cu2	Bi22-I15,Bi21-I15,Bi19-I14,Bi21-I14	2.98E-02	4.32E-17	5.28E-02	1.71E-02	-2.09E-02	-3.86E-03	8.19E-01	1.23E+00	-1.27E-01	5.74E-01
	Cu-I(μ2)		Cu31-I6,Cu31-I7	4.72E-02	1.17E-16	1.25E-01	3.85E-02	-4.58E-02	-7.24E-03	8.42E-01	1.19E+00	-1.54E-01	8.17E-01
	Cu-I(μ3)		Cu32-I10,Cu33-I11	5.43E-02	6.61E-15	1.40E-01	4.57E-02	-5.64E-02	-1.07E-02	8.10E-01	1.23E+00	-1.97E-01	8.42E-01
	Cu-I(eta4)		Cu32-I14, Cu32-I15,Cu33-I14,Cu33-I15	4.36E-02	1.22E-14	1.10E-01	3.38E-02	-4.02E-02	-6.34E-03	8.43E-01	1.19E+00	-1.45E-01	7.76E-01
**4**	Sb-I(eta1)	Sb-I	Sb93-I81,Sb93-I80,Sb93-I82,Sb94-I83,Sb94-I84,Sb94-I85	5.84E-02	3.78E-16	2.72E-02	2.98E-02	-5.27E-02	-2.30E-02	5.65E-01	1.77E+00	-3.93E-01	5.10E-01
	Sb-I(μ2)	Sb-I-Sb	Sb94-I79,Sb92-I79	3.51E-02	1.34E-13	3.21E-02	1.76E-02	-2.71E-02	-9.56E-03	7.56E-01	1.39E+00	-1.96E-01	5.04E-01
	Sb-I(μ3)	Sb2-I-Cu	Sb94-I78,Sb92-I78,Sb92-I77,Sb93-I77	2.76E-02	1.39E-14	3.26E-02	1.35E-02	-1.89E-02	-5.36E-03	8.15E-01	1.28E+00	-1.36E-01	4.94E-01
	Sb-I(μ3)	Sb2-I-Cu	Sb94-I74,Sb92-I74,Sb92-I75,Sb93-I75	2.73E-02	2.43E-16	3.59E-02	1.36E-02	-1.83E-02	-4.65E-03	8.09E-01	1.27E+00	-1.33E-01	5.02E-01
	Cu-I(μ3)	Cu-I-Sb	Cu73-I77,Cu73-I78	3.97E-02	2.82E-15	1.15E-01	3.24E-02	-3.62E-02	-3.72E-03	8.97E-01	1.11E+00	-9.35E-02	8.16E-01
	Cu-I(μ3)	Cu-I-Sb2	Cu71-I75	4.31E-02	8.29E-15	1.23E-01	3.57E-02	-4.07E-02	-4.94E-03	8.79E-01	1.14E+00	-1.15E-01	8.29E-01
	Cu-I(μ3)	Cu-I-Sb2	Cu72-I74,Cu72-I76	4.40E-02	1.20E-16	1.24E-01	3.66E-02	-4.20E-02	-5.49E-03	8.74E-01	1.15E+00	-1.21E-01	8.29E-01
**5**	Sb-I(μ1)	Sb-I	Sb91-I80,Sb91-I81,Sb91-I82	5.80E-02	1.35E-16	2.94E-02	2.98E-02	-5.23E-02	-2.25E-02	5.70E-01	1.75E+00	-3.88E-01	5.15E-01
	Sb-I(μ1)	Sb-I	Sb90-I77,Sb90-I78,Sb90-I79	5.76E-02	1.90E-13	3.28E-02	3.01E-02	-5.20E-02	-2.19E-02	5.79E-01	1.73E+00	-3.81E-01	5.23E-01
	Sb-I(μ1)	Sb-I	Sb89-I69,Sb89-I70	4.32E-02	8.04E-17	3.65E-02	2.14E-02	-3.36E-02	-1.22E-02	6.36E-01	1.57E+00	-2.83E-01	4.95E-01
	Sb-I(μ4)	Sb-I-Cu3	Sb91-I74,Sb91-I75,Sb91-I76	1.80E-02	9.53E-17	3.28E-02	9.00E-03	-9.80E-03	-8.08E-04	9.18E-01	1.09E+00	-4.50E-02	5.01E-01
	Sb-I(μ2)	Sb-I-Sb	Sb90-I66,Sb90-I67,Sb89-I66,Sb89-I67	2.59E-02	4.90E-15	3.23E-02	1.23E-02	-1.65E-02	-4.22E-03	7.91E-01	1.29E+00	-1.36E-01	4.72E-01
	Sb-I(μ2)	Sb-I-Cu	Sb89-I68	4.24E-02	1.02E-16	4.22E-02	2.18E-02	-3.31E-02	-1.13E-02	6.59E-01	1.52E+00	-2.66E-01	5.15E-01
	Sb-I(μ3)	Sb2-I-Cu	Sb89-I65,Sb90-I65	2.51E-02	2.10E-14	3.68E-02	1.27E-02	-1.62E-02	-3.51E-03	8.35E-01	1.22E+00	-1.12E-01	5.04E-01
	Cu-I(μ2)	Cu-I-Sb	Cu30-I68	4.70E-02	6.20E-16	1.29E-01	3.91E-02	-4.59E-02	-6.86E-03	8.51E-01	1.18E+00	-1.46E-01	8.31E-01
	Cu-I(μ3)	Cu-I-Sb2	Cu30-I65	4.67E-02	2.10E-15	1.29E-01	3.88E-02	-4.55E-02	-6.68E-03	8.53E-01	1.17E+00	-1.43E-01	8.31E-01
	Cu-I(μ4)	Cu3-I-Sb	Cu32-I74,Cu34-I76,Cu34-I74,Cu35-I74,Cu35-I75,Cu36-I76,Cu36-I75,Cu33-I75,Cu31-I76	4.29E-02	4.95E-14	1.20E-01	3.52E-02	-4.05E-02	-5.24E-03	8.75E-01	1.14E+00	-1.18E-01	8.17E-01

Figure 45. Rendering of the HOMO-3 for **4** showing significant Sb s and I p shell character.

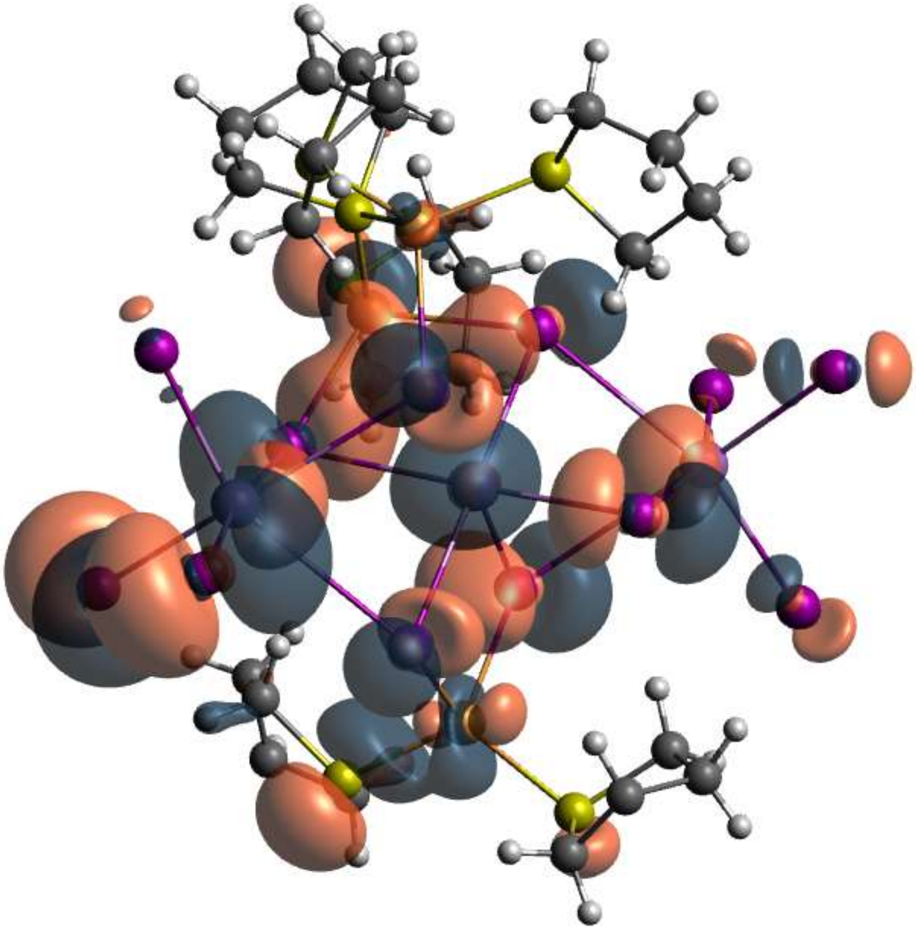

Supplement: Supplementary file 1 — ic4c01147_si_001.pdf [file ic4c01147_si_001.pdf]
